# How molecular architecture defines quantum yields

**DOI:** 10.1038/s41467-024-50366-1

**Published:** 2024-07-17

**Authors:** Fred Pashley-Johnson, Rangika Munaweera, Sheikh I. Hossain, Steven C. Gauci, Laura Delafresnaye, Hendrik Frisch, Megan L. O’Mara, Filip E. Du Prez, Christopher Barner-Kowollik

**Affiliations:** 1 School of Chemistry and Physics, Queensland University of Technology (QUT), 2 George Street, 4000 Brisbane, QLD Australia; 2 Centre for Materials Science, Queensland University of Technology (QUT), 2 George Street, 4000 Brisbane, QLD Australia; 3 Polymer Chemistry Research Group, Centre of Macromolecular Chemistry (CMaC), Department of Organic and Macromolecular Chemistry, Faculty of Sciences, Ghent University, Krijgslaan 281-S4, Ghent, 9000 Belgium; 4 Australian Institute for Bioengineering and Nanotechnology, The University of Queensland, Building 75, Cnr College Rd & Cooper Road, 4072 St Lucia, QLD Australia; 5 Institute of Nanotechnology (INT), Karlsruhe Institute of Technology (KIT), Hermann-von-Helmholtz-Platz 1, 76344 Eggenstein-Leopoldshafen, Germany

**Keywords:** Photochemistry, Polymers, Mechanical properties

## Abstract

Understanding the intricate relationship between molecular architecture and function underpins most challenges at the forefront of chemical innovation. Bond-forming reactions are particularly influenced by the topology of a chemical structure, both on small molecule scale and in larger macromolecular frameworks. Herein, we elucidate the impact that molecular architecture has on the photo-induced cyclisations of a series of monodisperse macromolecules with defined spacers between photodimerisable moieties, and examine the relationship between propensity for intramolecular cyclisation and intermolecular network formation. We demonstrate a goldilocks zone of maximum reactivity between the sterically hindered and entropically limited regimes with a quantum yield of intramolecular cyclisation that is nearly an order of magnitude higher than the lowest value. As a result of the molecular design of trifunctional macromolecules, their quantum yields can be deconvoluted into the formation of two different cyclic isomers, as rationalised with molecular dynamics simulations. Critically, we visualise our solution-based studies with light-based additive manufacturing. We formulate four photoresists for microprinting, revealing that the precise positioning of functional groups is critical for resist performance, with lower intramolecular quantum yields leading to higher-quality printing in most cases.

## Abstract (web summary)

Chemical topology is important in bond-forming reactions, but understanding the relationship between them is challenging. Here, the authors report a study considering photo-induced cyclisations for a series of monodisperse macromolecules with defined spacers and apply their findings to 3D printing.

## Introduction

Photochemical transformations are essential to both life^1^ and contemporary society^2^, and will continue to play an increasingly integral role as we proceed through the 21^st^ century. The significance of utilising light to control chemical reactions is further emphasised in the shift towards greener synthetic methods in industry^3^, and the demand for spatiotemporal control over reaction conditions^4^. Currently, the main limiting factors for the incorporation of photochemical processes into industry are the low photoproduct selectivity and yields of the reactions, the extensive cooling required due to the heat influx of the commonly employed large mercury lamps, and the limited scalability of reactor vessels due to the exponential nature of Beer-Lambert light attenuation^5–7^. Since the 1960s, some of these problems have been partially negated by the advent of light emitting diodes with long lifetimes, well-defined emission spectra and excellent energy efficiency^8^. However, these challenges can only be completely solved if the efficiency of the photochemical processes themselves is high.

The efficiency of photochemical processes is usually described in terms of quantum yield, defined as the number of photochemical events that occur per photon of light absorbed^9^. The quantum yield is used to assess photochemical processes such as the efficiency of fluorescence and phosphorescence^10^, photochemical polymerisations^11^, photolysis^12^, and photochemical bond-forming reactions^13^. Many factors have been shown to impact luminescence quantum yields including solvent^14^, temperature^15^, and excitation wavelength^16^. Our group has recently demonstrated—via photochemical action plots^17,18^—that the quantum yields of many photo-dissociations, -dimerisations^19,20^, and -polymerisations^21^ display a wavelength dependence that is often strongly disparate to the absorption profile of the starting material(s). This phenomenon is currently being explored in depth and often manifests as a red-shift, with the maximum photochemical activity occurring at wavelengths with far lower molar extinction coefficients.

One factor that has been shown to increase reaction yields and rates significantly in chemical reactions is the design of specific molecular environments. In catalysis, confinement of reagents into small spaces has proven effective for lowering energetic requirements^22^. Nanoreactors have also become common in settings such as photochemical upconversion^23^, polymer synthesis^24^, and photocatalysis^25^, often enabling low absolute reactant concentrations, whilst retaining their ability to react with each other by keeping the local concentrations high. For example, single chain nanoparticles have been shown to enhance catalysis through such confinement effects^26^. Although these intramolecular reactions are promising, they are still limited by the fact that additional reagents must be used, and that the precise local concentrations are not perfectly defined as a result of the disperse nature of the polymer and the changing local concentration during the folding process^27^. MacGillivray and colleagues have shown in several studies that photochemical reactions are able to be efficiently templated in the solid state, too, using structural motifs such as hydrogen bonding^28^ and metal coordination in crystals^29^. This templating improves reaction efficiency, enabling photochemical reactions that are not otherwise possible^30^, and facilitates the construction of complex molecular structures such as ladderenes^31^. More recently, Frisch and colleagues showed that peptides can be used as templates in the liquid state, inducing self-assemblies that showcase pH-gated control over [2 + 2] photocycloadditions in extremely dilute conditions^32^. The geometry required for [2 + 2] photocycloadditions to take place has also been used to stabilise specific macromolecular geometries, locking dynamic assemblies into position^33^. While these studies show that there is a relationship between architecture and efficiency of bond-forming reactions, it is yet to be systematically explored in solution.

**Fig. 1:**
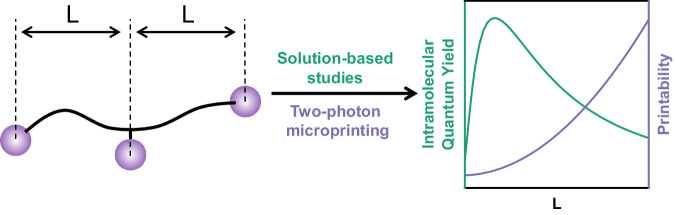
Schematic summary of the study presented herein. (left) Schematic representation of the trifunctional macromolecules synthesised in the current study showing L, the distance between terminal and central functional units. (right) Schematic representation of how a varying L is able to impact both the quantum yield of intramolecular cyclisation of the macromolecules and the printability when applied to two-photon microprinting.

Herein, we close this critical research gap by showing the precise impact that molecular architecture has on the efficacy of intramolecular photoreactions and how this subsequently determines intermolecular network formation along with the resulting material properties. By synthesising a library of four monodisperse macromolecules functionalised with three photo-dimerisable units that have variable spacing between the photoreactive groups (L, Fig. 1), we reveal a goldilocks zone of maximum reactivity between the entropically and sterically limited constraints (schematically represented in green in Fig. 1), as well as relating the quantum yield to specific reaction outcomes. Critically, two-photon microprinting has been used as a tool to visualise the impact that the variation in quantum yield has on the printability (schematically represented in purple in Fig. 1) and material properties.

## Results and discussion

### Synthesis of monodisperse, trifunctional macromolecules

**Fig. 2:**
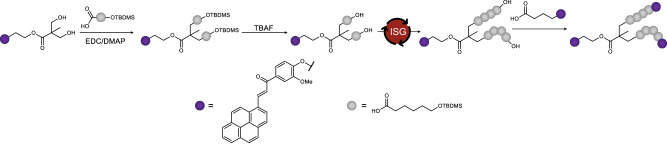
Reaction scheme for the synthesis of the molecules synthesised in this study. General reaction scheme for the synthesis of monodisperse, trifunctional macromolecules. Purple dots represent PyChal-functionalised units and grey dots represent a caprolactone units. ISG iterative sequential growth.

Firstly, we developed a synthetic method (summarised in Fig. 2) to enable the fabrication of monodisperse macromolecules with tuneable spacer length between three photoreactive units. The design of the trifunctional crosslinkers was motivated by our goal to directly visualise the impact that the efficacy of intramolecular cyclisation has on two-photon microprinting. To ensure a well-defined network structure, we selected a chromophore that is able to undergo a [2 + 2] photodimerisation upon irradiation with visible light—namely, pyrene-chalcone (PyChal)^19,34^. We selected an ε-caprolactone-based backbone due to its regular use as a backbone in light-driven 3D printing^35,36^, its large linker size enabling facile synthesis of structures with significant difference in spacing between reactive units, and the breadth of literature available showing the enhanced crystal formation and thermal properties when molecularly defined^37,38^.

Initially, we synthesised a difunctional alcohol bearing a PyChal moiety that acts as an initiator for the subsequent sequential addition steps. Next, an acid bearing a protected alcohol functionality is coupled to the initiator affording a doubly-protected diol with one extra linker unit. Here, we exploit the hydrophobicity of the chalcone initiator—and the resulting oligomers—to allow us to purify exclusively by aqueous extractions from the organic reaction mixture. As a result, the need for laborious and costly column chromatography in the sequence synthesis is completely negated. The doubly protected chain end of the growing macromolecule can be efficiently and rapidly removed with tetrabutylammonium fluoride to obtain a diol, which can then be further reacted to grow the chain. Detailed experimental procedures can be found in the supplementary information, section 2.4.

Once a suitable linker length had been synthesised, the chain end was functionalized by coupling with a carboxylic acid-functionalised PyChal, producing a trifunctional macromolecule that can be crosslinked upon irradiation with visible light. For the present study, we synthesised **T0,** **T1,** **T3**, and **T5**—where the number denotes the number of caprolactone units between each of the PyChal-bearing functionalities—(structures shown in section 2.5 of the supplementary information document).

### Efficiency of intramolecular cyclisation

To investigate how the molecular architecture influences the efficiency of the intramolecular cyclisation reaction, the quantum yield of the reaction was determined. Initially, we confirmed the formation of the [2 + 2] photocycloadduct upon irradiation of **T0-5** in an acetonitrile solution by monitoring the NMR resonances of the cyclobutane protons (Supplementary Fig. 52). Acetonitrile was selected as the solvent for our study for both consistency with our previous work on the PyChal chromophore^19^, and the lack of solute-solvent interactions that can occur, such as hydrogen bonding, that could influence the photochemistry. We started by irradiating low concentration (25 μM in acetonitrile) solutions of macromolecules **T0-5** using a monochromatic tuneable pulsed laser, and simultaneously recording the absorbance spectrum, enabling us to graph the conversion of **T0-5** into their cyclic counterparts vs. the number of photons. By fitting the linear section of this curve with Eq. (1)—where *ρ* represents the conversion to dimer, *Φ*_*c*_ is the intramolecular quantum yield, *N*_*p*_ is number of photons, *c* is concentration, *V* is volume, *A* is the extinction at 445 nm, and *N*_*A*_ is Avogadro’s number—the quantum yield of intramolecular cyclisation can be directly extracted from the gradient of the linear section of the graph (Supplementary Fig. 49) and plotted against the average spacing between reactive units.

1 $$\rho=\Delta {\varPhi }_{c}{N}_{p}\,{{;}} \, \Delta=\frac{2(1-{10}^{-A})}{c \cdot V \cdot {N}_{A}}$$

When the reactive units are positioned too closely together (**T0**), we observe that the quantum yield is low, caused by steric hindrance that is preventing the ready fulfilment of Schmidt’s topochemical postulate. According to this postulate, the parallel or antiparallel approach of the two dimerising units is required within 0.35–0.42 nm for dimerisation to take place^39^. Upon the inclusion of a single monomer unit between the central and terminal reactive units (for **T1**), the quantum yield of the intramolecular reaction increases by a factor of more than seven, attributed to the increased flexibility, which significantly increases the likelihood of fulfilling Schmidt’s postulate. By further increasing the average PyChal—PyChal distance, the efficiency of the cyclisation decreases as a result of the lower local concentration of PyChal in the larger macromolecules with increasing entropy, meaning that the probability of cyclisation slowly decreases until it is the same as the bulk (Fig. 1, right). Interestingly, this mirrors a trend observed for excimer fluorescence formation that has been described in several studies^40,41^, and later used quantitatively as a measure for the spatial arrangement of chromophore-bearing molecules^42,43^. However, the transition to bond-forming reactions shifts the maximum reactivity to a much longer distance, highlighting the importance of the geometry of the reacting chromophores in space, and the additional limitations that must be considered when designing systems with bond-forming systems.

**Fig. 3:**
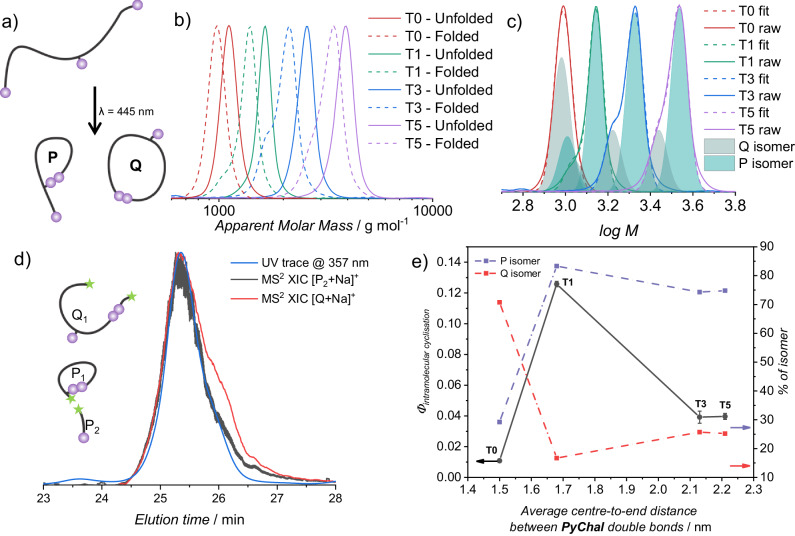
Data gathered that underpins the fundamental photochemistry of the molecules T0-5. **a** Two possible isomers formed by intramolecular dimerisation; (**b**) Size-exclusion chromatographs of the four macromolecules **T0-5** before (solid) and after (dashed) intramolecular folding upon irradiation of a 12.5 µM solution of the analyte in acetonitrile with a 10 W LED, λ_max_ = 445 nm; (**c**) Deconvolution of SEC traces by fitting with two monodisperse peaks to show the large and the small isomer that form upon irradiation; (**d**) SEC-ESI-(MS)^2^ data showing that the ‘*P*’ isomer has a larger hydrodynamic volume than the ‘Q’ isomer; (**e**) overlay of the percentages of the P and Q isomer. Left axis shows intramolecular cyclisation quantum yield (dotted grey lines), right axis shows percentage of P and Q isomers present (purple and red respectively). Intramolecular cyclisation quantum yield is calculated as highlighted in section 2.7 of the supplementary information, using the data presented in Supplementary Fig. 48. The raw data values can be found in Supplementary Table 2. Error bars show the mean value ± SD, *n* = 3.

The intramolecular cyclisation reaction can result in two structural isomers, either a structure resembling the letter *P* (chain end to centre dimerisation) or the letter Q (chain end to chain end dimerisation) (Fig. 3a).

We thus determined which of the two possible reaction outcomes was most favourable for each macromolecule. In a low concentration regime (12.5 μM), the intermolecular dimerisation reactions can be completely suppressed on short timescales, allowing us to investigate the isomer distribution via size exclusion chromatography (SEC)^44^. By triggering the intramolecular cyclisation under dilute conditions using a 10 W light emitting diode (LED, *λ*_*max*_ = 445 nm), we were able to observe a shift to lower apparent molecular weight for each of the macromolecules that is characteristic of intramolecular folding (Fig. 3b)^45^. Notably, the SEC traces of the folded macromolecules all feature a bimodal distribution, which is attributed to the difference in hydrodynamic volume between the P and Q conformation as shown in Fig. 3a. By fitting the peaks with two monodisperse distributions using the tool developed by Konkolewicz and colleagues (Fig. 3c)^46^, we were able to deconvolute the P and Q isomer based on their hydrodynamic volumes. Moreover, using a tandem SEC-ESI-(MS)^2^ system^47^, we were able to determine the identity of each isomer. Indeed, by probing the secondary-ion-extracted-ion-chromatographs for diagnostic fragments of each macromolecule, i.e. the tail fragment of the P isomer, we can determine at which time the P isomer elutes. The ratio of the tail fragment to molecular ion is higher at shorter retention times (Fig. 3d), indicating that the P isomer has a higher hydrodynamic volume than the Q isomer, consistent with the literature on controlled folding^48^. When overlaid with the aforementioned quantum yields in Fig. 3e, it is clear that more efficient bond formation occurs when there is a higher fraction of the large isomer P present in the isotopic mixture.

### Statistical sampling of molecular conformations

**Fig. 4:**
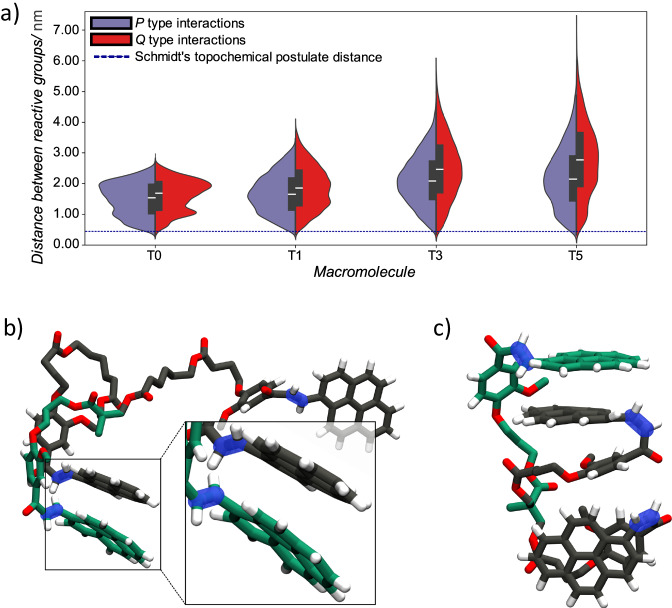
Results from molecular dynamics simulations supporting the photochemical conclusions. **a** Split violin plots showing the mean distances (white bars) with standard deviations (black bars) measured between photoactive double bonds in middle PyChal ring to terminal PyChal rings (interactions that promote P type isomerisation) and terminal PyChal ring to the other PyChal ring (interactions that promote Q type isomerisation) calculated across the 1500 ns simulations for **T0**, **T1**, **T3** and **T5**. The blue dashed line shows the maximum distance of 0.42 nm, between the photoactive double bonds for which photodimerisation can occur; (**b)** Close proximity of photoactive double bonds (highlighted in transparent blue) in the central (green) and terminal (black) PyChal arms results in parallel π-π stacking between the same two arms in **T1**; (**c)** Extended intramolecular PyChal π-π stacking in **T0** leads to Q type isomerisation. Oxygens are shown in red.

To better understand the impact of molecular architecture on the efficiency of intramolecular cyclisations, we conducted molecular dynamics (MD) simulations on **T0-5**. By conducting simulations of each molecule solvated explicitly in acetonitrile for 1500 ns, we are able to examine the range of conformations each molecule adopts in solution. We initially found that **T1,** **T3**, and **T5** have more independent flexibility in each arm than **T0**. The root mean square deviation (RMSD) provides a normalised distance of each atom in the macromolecule from its initial conformation over the combined 1500 ns simulation, giving a measure of the flexibility of each polymer. The RMSD increases from 0.80 ± 0.12 nm in **T1**, to 1.17 ± 0.22 nm in **T3** and 1.18 ± 0.21 nm in **T5**, indicating that polymer flexibility increases with increasing arm length as expected. However, for **T0**, the reduced RMSD of 0.69 ± 0.11 nm indicates that the absence of any aliphatic linker limits the flexibility. Through cluster analysis of the conformational distributions of **T0-5**, we saw that each macromolecule preferentially adopted conformations that promoted π-π stacking between the pyrene moiety and substituted benzene rings within a PyChal unit (Supplementary Fig. 50). In the 1500 ns of MD simulation, **T0,** **T1,** **T3** and **T5** adopted these conformations for 86.6% (1299 ns), 93.6% (1404 ns), 91.8% (1377 ns), and 95.6% (1434 ns), respectively, indicating that these π-π stacked conformations are energetically favourable. However, these conformations did not facilitate photoreactions since Schmidt’s topochemical postulate could not be fulfilled^39^. To identify a subset of conformations that fulfil Schmidt’s postulate from MD simulations, we examined the pairwise distance between the first carbons of the three photoreactive double bonds (Supplementary Fig. 51). In agreement with the experimental results for **T1-5**, we found that the average distance between the photoreactive groups in the terminal and central PyChal arms were closer than the two terminal PyChal arms (Fig. 4a), indicating they are more likely to sample interactions that promote cyclisation to the P isomer rather than the Q isomer.

However, the P isomer average distances ranged from 1.50 ± 0.52 nm for **T0**, to 1.68 ± 0.62 nm in **T1**, to 2.13 ± 0.80 nm in **T3** and 2.21 ± 0.96 nm in **T5**, indicating that macromolecular conformations that facilitate photodimerisation are statistically a rare event. We found that in all four macromolecules, parallel π-π stacking between PyChal units promotes a conformation that minimises the distance between the photoreactive double bonds, while antiparallel π-π stacking is associated with longer distances between the photoreactive double bonds (Fig. 4b, c).

Unlike **T1-5**, we found experimentally that **T0** has a propensity for Q-isomer formation despite the P-type interaction being closer than the Q-type interaction (Fig. 4a). By examining the structure of the energetically favourable states in the simulation period, we found a distinct conformation for **T0** in which a π-stacking network is formed through the three PyChal units that persisted for 1.4% of the total simulation time (Fig. 4c). This low enthalpy conformation of **T0** involves an edge-to-face π-π interaction between the terminal PyChal arms, and an antiparallel face-to-face π-stacking of the photoactive domains of the central and a terminal PyChal. The steric constraints of the more compact **T0** prevent the transition to a parallel face-to-face π-stacking arrangement that is required for the P-type photocycloaddition reaction between the central and terminal PyChals (Fig. 4c), meaning that P*-*isomer formation becomes improbable.

The proximity and orientation of the photoactive reactive groups are critical for [2 + 2] cycloadditions. Our simulations revealed that these are facilitated by the π-π stacking interactions between opposing PyChal groups, which promote an enthalpically favourable conformation whilst negatively contributing to entropy. The trade-off between enthalpy and entropy dictates the efficiency of the photocycloaddition reaction. **T0** has low flexibility, showing a tendency to undergo photocycloaddition, yielding mainly the Q isomer. However, the low enthalpy conformation is a rare event, decreasing the probability of dimerisation overall. In contrast, the increased conformational flexibility and entropy of **T1,** **T3** and **T5** increased the sampling of conformations leading to P isomer formation. Longer linkers also decrease the propensity to form a compact state with π-π stacking between PyChal arms. The PyChal arm lengths of **T1** identify the enthalpic/entropic goldilocks balance that is required for a P-type photocycloaddition, as clearly reflected by the higher quantum yields of **T1** (Fig. 3e).

### Quantum efficiency visualisation via two-photon microprinting

**Fig. 5:**
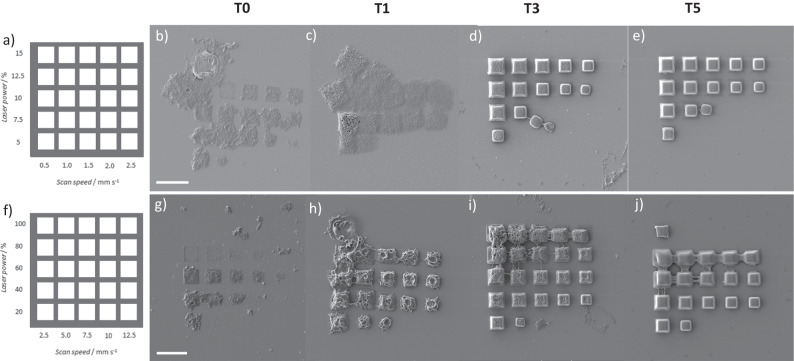
Two sets of micro-cube arrays with changing laser power on the Y-axis and changing scan speed on the X-axis. **a**–**f** Schematics showing the parameters used to print the two different arrays; (**b**–**e)** SEM images of micro-cube arrays printed with each macromolecule using lower scan speeds and laser powers; (**g**–**j)** SEM images of micro-cube arrays printed with each macromolecule using higher scan speeds and laser powers; scale bars = 20 µm.

In order to investigate the effects of varying the quantum yield based on molecular architecture in the realm of additive manufacturing, we employed two-photon microprinting. Four resists were formulated by dissolving **T0-5** in a mixture of propylene carbonate: acetophenone (3:2 volume:volume ratio, *C* = 93.9 µmol L^-1^), a solvent system selected for its high boiling point and good solubilising properties for large aromatic hydrocarbons. These four resists were subsequently printed using a Nanoscribe two-photon printer and imaged via scanning electron microscopy (SEM)^49^. To access the performance of each of the four resists, two arrays of 25 micro-cubes with 10 µm side length were printed onto a glass substrate with laser power and scan speeds varying between 5–100% and 0.5–12.5 mm s^-1^ respectively (Fig. 5). When the exposure is low, i.e., lower laser power and scan speeds (Fig. 5a–e), it is clear that the printing quality increases as the PyChal—PyChal distance increases, with **T0** featuring the worst print quality, through to **T5** being the best performing resist. The trend is mirrored when the exposure is high, i.e., higher laser power and scan speed arrays (Fig. 5f–h). One explanation for the decrease in printing quality for smaller PyChal—PyChal distance is that the quantum yield of intramolecular cyclisation increases, removing potential sites for intermolecular crosslinks, and thus jeopardising network formation. The effect is best exemplified by the drastic difference in print quality between **T1** (Fig. 5c) and **T3** (Fig. 5d), where the increased intramolecular quantum yield for **T1** manifests as poor print quality. The exception to the trend is **T0** (Fig. 5b, g), whose quantum yield is low, but print quality is poor, due to the enthalpically favoured π-stacked structure (Fig. 4c) that sterically shields the reactive double bonds from intermolecular attack.

It is important to note that the tendency for micro-explosions is far higher for the macromolecules with smaller PyChal—PyChal distances, manifesting itself as holes in the structures and as lumps for smaller explosions (seen with higher laser powers for **T3**, Fig. 5i). At higher laser powers and lower scan speeds, the micro-cubes fuse together due to the thermal swelling that takes place during the printing process (evident in Fig. 5j). Consequently, the next print begins inside the over-sized structure before it relaxes to its deswollen volume, resulting in the bridges between structures.

**Fig. 6:**
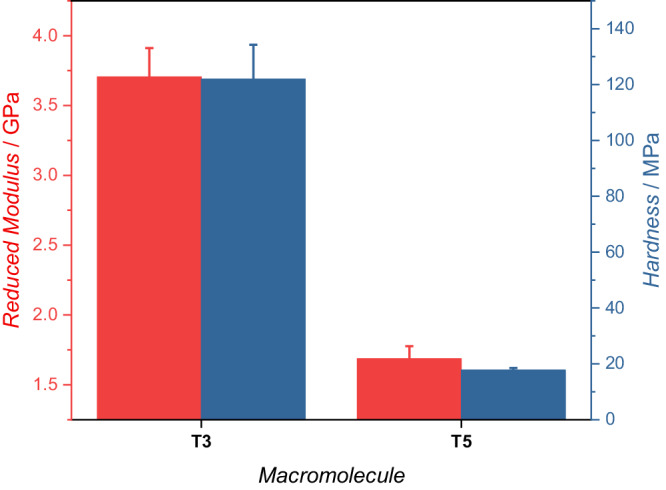
Material properties of the printed structures. The reduced modulus (red) and hardness (blue) of 25 × 25 x 20 μm blocks of **T3** (left) and **T5** (right) determined by displacement-controlled nanoindentation to a depth of 1000 μm. Error bars show the standard deviation of 7 measurements.

To quantify the difference in material properties between the printed **T3** and **T5** structures, two sets of identical blocks, 25 × 25 x 20 μm, printed using identical printing parameters (laser-power = 15, scan speed = 1.5 mm s^-1^), were probed using displacement-controlled nanoindentation. These measurements revealed a pronounced difference in both the modulus and the hardness of the structures (Fig. 6), which is attributed to their difference in network densities. **T3** has a much higher reduced modulus and hardness than **T5** due to the shorter linkers, creating a more tightly-packed network structure^50^.

**Fig. 7:**
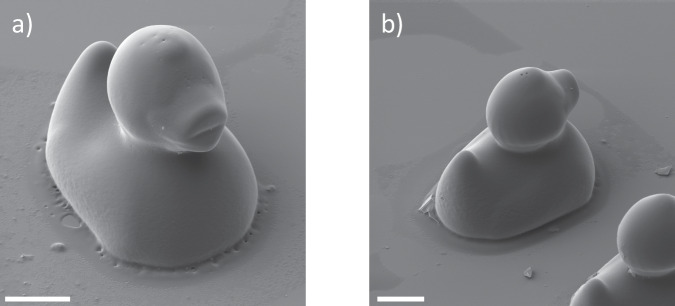
3D-printed rubber duck structures. SEM images of rubber duck structures printed from the **T5**-containing resist with a laser power of 80% and a scan speed of 7 mm s^−1^ (**a**) from the front and (**b**) from the back. Scale bars = 20 μm.

Finally, we demonstrated that despite the extremely low concentration compared to other pre-polymer based two-photon microprinting resists^51,52^, we were able to print 3D-structures with good feature resolution, and at a relatively large scale. Figure 7 shows two rubber duck structures with a height of 100 μm, fabricated from the **T5**-containing resist. With the rapid printing speed achievable with these resists, compared to other photo-cycloaddition-based systems^51^, such structures are able to be printed on short timescales (8 min and 15 s for each structure). The pronounced overhanging features can be well resolved (a feature that is also demonstrated by the boxing rings shown in Supplementary Fig. 53) and demonstrate that these [2 + 2] photocycloaddition-based resins have promise in two-photon microprinting going forward^53^.

We demonstrate—through the design of a bespoke library of macromolecules—that molecular architecture is critical to their photoreactivity, specifically in their application to two-photon microprinting. By decreasing the spacing between functional groups within a molecular framework, the entropic limitations on the efficacy of their reactions can be overcome and the intramolecular cyclisation quantum yield can be significantly increased. This is valid until the steric limitations outweigh the entropic ones, resulting in Schmidt’s topochemical postulate not being fulfilled—a phenomenon that has been demonstrated both experimentally and theoretically herein. We reveal a goldilocks zone of maximum reactivity between these two constraints, a feature that has not previously been demonstrated via an in-depth study of the molecular architecture.

The observed photochemical reactivity was subsequently translated into light-based microprinting where we demonstrated the importance of molecular architecture on the printing process and resulting material properties. We show that the feasibility of a macromolecular structure for application in a photoresist strongly depends on the precise positioning of functional groups, with higher quantum yield of intramolecular cyclisation leading to poor print quality, and lower quantum yields enabling printing of large structures, highlighting the link between the intramolecular quantum yields and the resultant prints. Finally, we assessed the material properties of the printed structures, and found that despite higher print quality with longer linkers, the hardness of the material is compromised, indicating an important balancing act between material properties and print quality that will have to be considered when developing photoresists. These findings will prove critical in the development of new photoresists for advanced manufacturing, especially as more and more researchers turn to single-component photoresists with sophisticated crosslinking methodologies.

As we demonstrate the presence of a regime of maximum reactivity between sterically and entropically limited molecular design, we also hope that fields such as photocatalysis and medicinal chemistry will consider their molecular architecture carefully to achieve optimal performance.

## Methods

All experimental methods and analytical data are available in the supplementary information document.

### Supplementary information

Supplementary Information

Peer Review File

## Data Availability

The authors declare that the data supporting the findings of this study are available within the article and its Supplementary Information files. Raw data files are available from the corresponding authors upon request.

## Code availability

The essential molecular dynamics simulation data including input atomic coordinates, force field files, mdp file, and output coordinates and trajectories utilised in this study are accessible at https://github.com/OMaraLab/Trifunctional_macromolecules_isomerization.

## References

[1] Rohatgi-Mukherjee, K. *Fundamentals Of Photochemistry* Vol. 347 (New Age International, 1978).

[2] Garcia-Garibay MA (2012). Advances at the frontiers of photochemical sciences. J. Am. Chem. Soc..

[3] Oelgemöller M, Jung C, Mattay J (2007). Green photochemistry: production of fine chemicals with sunlight. Pure Appl. Chem..

[4] Aubert S, Bezagu M, Spivey AC, Arseniyadis S (2019). Spatial and temporal control of chemical processes. Nat. Rev. Chem..

[5] Loubière K, Oelgemöller M, Aillet T, Dechy-Cabaret O, Prat L (2016). Continuous-flow photochemistry: a need for chemical engineering. Chem. Eng. Process..

[6] Sambiagio C, Noël T (2020). Flow photochemistry: shine some light on those tubes!. Trends Chem..

[7] Cohen B (2023). Emerging reaction technologies in pharmaceutical development: challenges and opportunities in electrochemistry, photochemistry, and biocatalysis. Chem. Eng. Res. Des..

[8] Noël, T., Escriba Gelonch, M. & Huvaere, K. in *Photochemical Processes in Continuous-Flow Reactors* (ed Timothy Noël) 284 (Eindhoven University of Technology, 2017).

[9] Braslavsky SE (2007). Glossary of terms used in photochemistry 3rd edition (IUPAC Recommendations 2006). Pure Appl. Chem..

[10] Levitus M (2020). Tutorial: measurement of fluorescence spectra and determination of relative fluorescence quantum yields of transparent samples. Methods Appl. Fluoresc..

[11] Neumann MG, Schmitt CC, Ferreira GC, Correa IC (2006). The initiating radical yields and the efficiency of polymerization for various dental photoinitiators excited by different light curing units. Dent. Mater..

[12] Gans B (2011). Photolysis of methane revisited at 121.6 nm and at 118.2 nm: quantum yields of the primary products, measured by mass spectrometry. Phys. Chem. Chem. Phys..

[13] Chow, Y. L., Buono-Core, G. E., Zhang, Y.-H. & Liu, X.-Y. Photocycloaddition of cyanonaphthalenes with acetylacetone: enhancement of quantum yields by sulphuric acid. *J. Chem. Soc. Perkin Trans. 2* 2041–2045, 10.1002/chin.199213105 (1991).

[14] Steen HB (1974). Wavelength dependence of the quantum yield of fluorescence and photoionization of indoles. J. Chem. Phys..

[15] Kubin RF, Fletcher AN (1982). Fluorescence quantum yields of some rhodamine dyes. J. Lumin..

[16] Köhler, G. & Getoff, N. Wavelength dependence of the fluorescence quantum yield of some substituted phenols. *J. Chem. Soc. Faraday Trans. 1* **72**, 2101–2107 (1976).

[17] Irshadeen IM (2021). Action plots in action: in-depth insights into photochemical reactivity. J. Am. Chem. Soc..

[18] Walden SL, Carroll JA, Unterreiner AN, Barner-Kowollik C (2024). Photochemical action plots reveal the fundamental mismatch between absorptivity and photochemical reactivity. Adv. Sci..

[19] Irshadeen IM (2021). Green light LED activated ligation of a scalable, versatile chalcone chromophore. Polym. Chem..

[20] Carroll, J. A., Pashley-Johnson, F., Frisch, H. & Barner-Kowollik, C. Photochemical action plots reveal red-shifted wavelength-dependent photoproduct dstributions. *Chem. Eur. J*. **30**, e202304174 (2024). 10.1002/chem.202304174 38267371

[21] Ma C (2022). Aggregation-induced emission poly(meth)acrylates for photopatterning via wavelength-dependent visible-light-regulated controlled radical polymerization in batch and flow conditions. Macromolecules.

[22] Shifa TA, Vomiero A (2019). Confined catalysis: progress and prospects in energy conversion. Adv. Energy Mater..

[23] Zhou Q (2023). Spatially controlled UV light generation at depth using upconversion micelles. Adv. Mater..

[24] Monteiro MJ (2010). Nanoreactors for polymerizations and organic reactions. Macromolecules.

[25] Wu C, Xing Z, Yang S, Li Z, Zhou W (2023). Nanoreactors for photocatalysis. Coord. Chem. Rev..

[26] Mundsinger K, Izuagbe A, Tuten BT, Roesky PW, Barner-Kowollik C (2024). Single chain nanoparticles in catalysis. Angew. Chem. Int. Ed..

[27] Frisch H (2018). Photochemistry in confined environments for single-chain nanoparticle design. J. Am. Chem. Soc..

[28] MacGillivray LR, Reid JL, Ripmeester JA (2000). Supramolecular control of reactivity in the solid state using linear molecular templates. J. Am. Chem. Soc..

[29] Chu Q, Swenson DC, MacGillivray LR (2005). A single-crystal-to-single-crystal transformation mediated by argentophilic forces converts a finite metal complex into an infinite coordination network. Angew. Chem. Int. Ed..

[30] MacGillivray LR (2008). Supramolecular control of reactivity in the solid state: from templates to ladderanes to metal−oganic frameworks. Acc. Chem. Res..

[31] Gao X, Friščić T, MacGillivray LR (2004). Supramolecular construction of molecular ladders in the solid state. Angew. Chem. Int. Ed..

[32] Richardson BJ (2023). Peptide self-assembly controlled photoligation of polymers. J. Am. Chem. Soc..

[33] Ren L (2023). Thermoresponsive helical dendronized poly(phenylacetylene)s: remarkable stabilization of their helicity via photo-dimerization of the dendritic pendants. J. Am. Chem. Soc..

[34] Van De Walle M, De Bruycker K, Blinco JP, Barner-Kowollik C (2020). Two colour photoflow chemistry for macromolecular design. Angew. Chem. Int. Ed..

[35] Arif ZU (2022). Recent advances in 3D-printed polylactide and polycaprolactone-based biomaterials for tissue engineering applications. Int. J. Biol. Macromol..

[36] Thijssen Q (2023). Volumetric Printing of Thiol-Ene Photo-Cross-Linkable Poly(ε -caprolactone): A Tunable Material Platform Serving Biomedical Applications. Adv. Mater..

[37] Takizawa K, Tang C, Hawker CJ (2008). Molecularly defined caprolactone oligomers and polymers: synthesis and characterization. J. Am. Chem. Soc..

[38] Duan SH (2021). A versatile synthetic platform for discrete oligo- and polyesters based on optimized protective groups iterative exponential growth. Macromolecules.

[39] Schmidt GMJ (1971). Photodimerization in the solid state. Pure Appl. Chem..

[40] Zachariasse KA, Maçanita AL, Kühnle W (1999). Chain length dependence of intramolecular excimer formation with 1,n-bis(1-pyrenylcarboxy)alkanes for n = 1−16, 22, and 32. J. Phys. Chem. B.

[41] Duhamel J (2014). Global analysis of fluorescence decays to probe the internal dynamics of fluorescently labeled macromolecules. Langmuir.

[42] McNelles SA, Thoma JL, Adronov A, Duhamel J (2018). Quantitative characterization of the molecular dimensions of flexible dendritic macromolecules in solution by pyrene excimer fluorescence. Macromolecules.

[43] Bieri O (1999). The speed limit for protein folding measured by triplet-triplet energy transfer. Proc. Natl Acad. Sci. USA.

[44] Frisch H, Tuten BT, Barner-Kowollik C (2020). Macromolecular superstructures: a future beyond single chain nanoparticles. Isr. J. Chem..

[45] Izuagbe AE, Truong VX, Tuten BT, Roesky PW, Barner-Kowollik C (2022). Visible light switchable single-chain nanoparticles. Macromolecules.

[46] De Alwis Watuthanthrige N (2020). Wavelength-controlled synthesis and degradation of thermoplastic elastomers based on intrinsically photoresponsive phenyl vinyl ketone. Macromolecules.

[47] Gruendling T, Guilhaus M, Barner-Kowollik C (2008). Quantitative LC-MS of polymers: determining accurate molecular weight distributions by combined size exclusion chromatography and electrospray mass spectrometry with maximum entropy data processing. Anal. Chem..

[48] Schmidt BV, Fechler N, Falkenhagen J, Lutz JF (2011). Controlled folding of synthetic polymer chains through the formation of positionable covalent bridges. Nat. Chem..

[49] Buckmann T (2012). Tailored 3D mechanical metamaterials made by dip-in direct-laser-writing optical lithography. Adv. Mater..

[50] Qu J, Kadic M, Naber A, Wegener M (2017). Micro-structured two-component 3D metamaterials with negative thermal-expansion coefficient from positive constituents. Sci. Rep..

[51] Gauci, S. C. et al. 3D Printed microstructures erasable by darkness. *Adv. Funct. Mater*. **33**, 2206303 (2022).

[52] Catt SO, Hackner M, Spatz JP, Blasco E (2023). Macromolecular engineering: from precise macromolecular inks to 3D printed microstructures. Small.

[53] Gauci SC (2024). Photochemically activated 3D printing inks: current status, challenges, and opportunities. Adv. Mater..

