# Supplementary material for: How molecular architecture defines quantum yields
Source: Nat Commun. 2024 Jul 17;15:6033. doi: 10.1038/s41467-024-50366-1 (PMC11255304; doi:10.1038/s41467-024-50366-1)
Supplement: Supplementary file 1 — Supplementary Information [file 41467_2024_50366_MOESM1_ESM.pdf]

How Molecular Architecture Defines Quantum Yields

Fred Pashley-Johnson, Rangika Munaweera, Sheikh I. Hossain, Steven C. Gauci, Laura Delafresnaye, Hendrik Frisch, Megan L. O'Mara*, Filip E. Du Prez*, and Christopher Barner-Kowollik*

1. Table of Contents

2. Supplementary Methods 2
2.1 Instrumentation 2
2.1.1 High performance liquid chromatography – mass spectrometry (LCMS) 2
2.1.2 THF size exclusion chromatography (SEC) 2
2.1.3 Bruker 600 MHz nuclear magnetic resonance (NMR) 2
2.1.4 Flash chromatography 2
2.1.5 UV/Vis spectroscopy 3
2.1.6 LED characterisation 3
2.1.7 Size-exclusion chromatography coupled with high resolution mass spectrometry (SEC-MS)[1] 3
2.1.8 Direct laser writing (DLW) 3
2.1.9 Scanning electron microscopy 3
2.1.10 Nanoindentation 3
2.1.11 10 W, 445 nm LED Emission Spectrum 4
2.2 Materials 4
2.3 Synthesis of small molecules 4
2.3.1 Synthesis of 6-hydroxyhexanoic acid (1) 4
2.3.2 Synthesis of 6-*tert*-butyldimethylsilyletherhexanoic acid (2) 5
2.3.3 Synthesis of 1-(4-(3-hydroxypropoxy)-3-methoxyphenyl)ethan-1-one (3) 6
2.3.4 Synthesis of (3-hydroxypropoxy)pyrene-chalcone (4) 7
2.3.5 Synthesis of acetonide-2,2-*bis*(methoxy) propionic acid (5) 9
2.3.6 Synthesis of dihydroxypyrene chalcone (6) 10
2.3.7 Synthesis of ethyl 4-(4-acetyl-2-methoxyphenoxy)butanoate (7) 16
2.3.8 Synthesis of (butyric acid)pyrene chalcone (8) 17
2.4 Iterative sequential growth procedures 17
2.4.1 General procedure for addition steps 17
2.4.2 General procedure for deprotection steps 18
2.5 Chemical structures of T0-5 18
2.6 Synthesis and characterisation of final molecules 19
2.6.1 General procedure for chain-end functionalisation with PyChal 19
2.6.2 NMR characterisation of each macromolecule 19
2.6.3 HRMS Characterisation of each macromolecule 38
2.6.4 Tabulated HRMS data for each molecule 40
2.7 Quantum yield determination 40
2.7.1 Experimental setup for quantum yield determination 40
2.7.2 Derivation of formula for quantum yield determination 41

2.7.3 Extinction spectra of T0-5 ... 42
2.7.4 Quantum yield fits ... 42
2.8 Molecular dynamics simulations ... 43
3. Supplementary Discussion ... 47
3.1 Confirmation of cycloaddition ... 47
4. Supplementary Figures ... 48
5. Supplementary References ... 48

2. Supplementary Methods

2.1 Instrumentation

2.1.1 High performance liquid chromatography – mass spectrometry (LCMS)

LC-MS measurements were performed on an UltiMate 3000 UHPLC System (Dionex, Sunnyvale, CA, USA) consisting of a pump (LPG 3400SZ), autosampler (WPS 3000TSL) and a temperature controlled column compartment (TCC 3000). Separation was performed on a C18 HPLC column (Phenomenex Luna 5µm, 100 Å, 250 × 2.0 mm) operating at 40 °C. Water (containing 5 mmol L^{-1} ammonium acetate) and acetonitrile were used as eluents. A gradient of acetonitrile: H_2O, 5:95 to 100:0 (v/v) in 7 min at a flow rate of 0.40 mL·min^{-1} was applied. The flow was split in a 9:1 ratio, where 90% of the eluent was directed through a DAD UV-detector (VWD 3400, Dionex) and 10% was infused into the electrospray source. Spectra were recorded on an LTQ Orbitrap Elite mass spectrometer (Thermo Fisher Scientific, San Jose, CA, USA) equipped with a HESI II probe. The instrument was calibrated in the *m/z* range 74-1822 using premixed calibration solutions (Thermo Scientific). A constant spray voltage of 3.5 kV, a dimensionless sheath gas, and a dimensionless auxiliary gas flow rate of 5 and 2 were applied, respectively. The capillary temperature was set to 300 °C, the S-lens RF level was set to 68, and the aux gas heater temperature was set to 100 °C.

2.1.2 THF size exclusion chromatography (SEC)

The SEC measurements were conducted on a PSS SECurity system consisting of a PSS SECurity Degasser, PSS SECurity TCC6000 Column Oven (35 °C), PSS SDV Column Set (8x150 mm 5 µm Precolumn, 8x300 mm 5 µm Analytical Columns, 100000 Å, 1000 Å and 100 Å) and an Agilent 1260 Infinity Isocratic Pump, Agilent 1260 Infinity Standard Autosampler, Agilent 1260 Infinity Diode Array and Multiple Wavelength Detector (A: 254 nm, B: 360 nm), Agilent 1260 Infinity Refractive Index Detector (35 °C). HPLC grade THF, stabilized with BHT, is used as eluent at a flow rate of 1 mL·min-1. Narrow disperse linear poly(styrene) (M_n 266 g·mol^{-1} to $2.52x10^6$ g·mol^{-1}) and poly(methyl methacrylate) (M_n 202 g·mol^{-1} to $2.2x10^6$ g·mol^{-1}) standards (PSS ReadyCal) were used as calibrants. All samples were passed over 0.22 µm PTFE membrane filters. Molecular weight and dispersity analysis was performed in PSS WinGPC UniChrom software (version 8.2).

2.1.3 Bruker 600 MHz nuclear magnetic resonance (NMR)

^1H and ^{13}C were recorded on a Bruker System 600 Ascend LH, equipped with a BBO-Probe (5 mm) with z-gradient (^1H: 600.13 MHz, ^{13}C 150.90 MHz). Resonances are reported in parts per million (ppm) relative to tetramethylsilane (TMS). The δ-scale was calibrated to the respective solvent signal of $CHCl_3$ or DMSO for ^1H spectra and for 13C spectra on the middle signal of the $CDCl_3$ triplet or the DMSO quintet. The annotation of the signals is based on HSQC-, COSY- and DEPT-experiments.

2.1.4 Flash chromatography

Flash chromatography was performed on an Interchim XS420+ flash chromatography system consisting of a SP-in-line filter 20-µm, an UV-VIS detector (200-800 nm) and a SofTA Model 400 ELSD (55 °C dift tube temperature, 25 °C spray chamber temperature, filter 5, EDR gain mode) connected via a flow splitter (Interchim Split ELSD F04590). The separations were performed using an Interchim dry load column (dryload on celite 565) and an Interchim Puriflash Silica HP 30 µm column.

2.1.5 UV/Vis spectroscopy

UV-Vis spectra were recorded on a Shimadzu UV-2700 spectrophotometer equipped with a CPS-100 electronic temperature control cell positioner. Samples were prepared in acetonitrile with a concentration of 2.18 µM and measured in Hellma Analytics quartz high precision cell cuvettes at room temperature.

2.1.6 LED characterisation

LED emission spectra were recorded using an Ocean Insight Flame-T-UV-Vis spectrometer, with an active range of 200-850 nm and an integration time of 10 ms. LED output energies were recorded using a Thorlabs S401C thermopile sensor, with an active area of 100 mm2 and a wavelength range of 190 nm – 20 µm, connected to a Thorlabs PM400 energy meter console. The emitted power from each LED was measured for 60 seconds at a fixed distance from the sensor, after which the mean and standard deviation of the emission could be determined. LEDs were cooled during measurement to minimise any thermal effects on the emission power or sensor performance.

2.1.7 Size-exclusion chromatography coupled with high resolution mass spectrometry (SEC-MS)[1]

Spectra were recorded on a Q Exactive Plus (Orbitrap) mass spectrometer (Thermo Fisher Scientific, San Jose, CA, USA) equipped with an HESI II probe. The instrument was calibrated in the m/z range 74-1822 using premixed calibration solutions (Thermo Scientific) and for the high mass mode in the m/z range of 600-8000 using ammonium hexafluorophosphate solution. A constant spray voltage of 3.5 kV, a dimensionless sheath gas and a dimensionless auxiliary gas flow rate of 10 and 0 were applied, respectively. The capillary temperature was set to 320 °C, the S-lens RF level was set to 150, and the aux gas heater temperature was set to 125 °C. The Q Exactive was coupled to an UltiMate 3000 UHPLC System (Dionex, Sunnyvale, CA, USA) consisting of a pump (LPG 3400SD), autosampler (WPS 3000TSL), and a temperature controlled column department (TCC 3000). Separation was performed on three mixed bead size exclusion chromatography columns (PSS, SDV micro columns 3µm 1000Å 4.6 x 250mm) with a precolumn (SDV micro precolumn 3µm 4.6x30mm) operating at 30 °C. THF at a flow rate of 0.30 $mL{\cdot}min^{-1}$ was used as eluent. The mass spectrometer was coupled to the column in parallel to an UV detector (VWD 3400, Dionex), and a RI-detector (RefractoMax520, ERC, Japan) in a setup described earlier.[1] 0.27 $mL{\cdot}min^{-1}$ of the eluent were directed through the UV and RI-detector and 30 $\mu L{\cdot}min^{-1}$ were infused into the electrospray source after post-column addition of a 50 µM solution of sodium iodide in methanol at 20 $\mu L{\cdot}min^{-1}$ by a micro-flow HPLC syringe pump (Teledyne ISCO, Model 100DM). A 100 µL aliquot of a polymer solution with a concentration of 2 $mg{\cdot}mL^{-1}$ was injected into the SEC system.

For experiments using tandem MS, normalised collision energy of 40 was used to fragment the selected mass of the target macromolecule's sodium adduct

2.1.8 Direct laser writing (DLW)

DLW was performed with a NanoScribe Photonic Professional GT with a Ti-Sapphire light source (760 nm) producing 100 fs, 80 MHz pulses. All structures were printed in galvo scan mode using a 63x oil immersion objective (NA 1.4)

2.1.9 Scanning electron microscopy

SEM images were captured using a Tescan MIRA3 scanning electron microscope operating at 3 kV with a beam intensity of 8. Samples were coated with 4 nm platinum prior to imaging.

2.1.10 Nanoindentation

Nanoindentation measurements were performed using a Hysitron TI950 Nanoindentor. A tip with the Berkovich geometry was used to perform all measurements. All measurements were carried out under depth control.

2.1.11 10 W, 445 nm LED Emission Spectrum

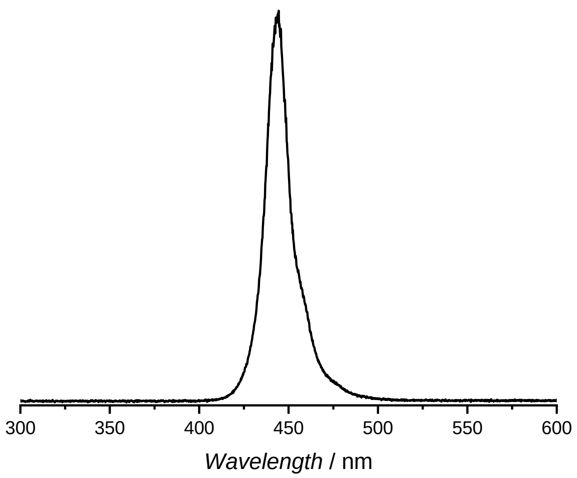

Supplementary Fig. 1. Emission profile for the 445 nm 10 W LED

2.2 Materials

Acetovanillone (98%, also called 4'-hydroxy-3'-methoxyacetophenone), ε-caprolactone (97%), 3-bromo-1-propanol (97%), imidazole (≥99.5%), 1-pyrenecarboxaldehyde (99%), 1-ethyl-3-(3-dimethylaminopropyl)carbodiimide hydrochloride, 4-(dimethylamino)pyridine (>99%), Dowex® 50WX8 hydrogen form 200-400 mesh, 2,2-Bis(hydroxymethyl)propionic acid (98%), 2,2-dimethoxypropane (98%) and tetrabutylammonium fluoride (1M solution in THF) were purchased from Sigma-Aldrich. *Tert*-butyldimethylsilyl chloride (98%), ethyl 4-bromobutyrate (98%) were purchased from Combi-Blocks. *p*-Toluenesulfonic acid monohydrate was purchased from Merck

All chemicals were used as received unless stated otherwise. Water was purified by an SP-1 Milli-Q purification system, and dichloromethane was purified by a solvent purification system.

2.3 Synthesis of small molecules

2.3.1 Synthesis of 6-hydroxyhexanoic acid (1)

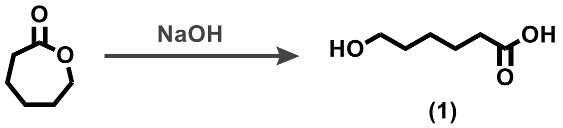

(1)

6-Hydroxyhexanoic acid was synthesised as described in the literature.[2,3] Sodium hydroxide (4.33 g, 108 mmol, 1.2 eq.) was dissolved in 200 mL Milli-Q water. ε-caprolactone (10 mL, 90 mmol, 1.0 eq.) was added and stirred overnight. Subsequently, concentrated hydrochloric acid (32% in water, 13 mL) was added to the solution to acidify to pH = 1. The aqueous solution was extracted with ethyl acetate (3 x 150 mL) – the combined organic phases were dried over anhydrous sodium sulphate and the solvent was removed under reduced pressure to give 8.6 g (72% yield) of a clear colourless oil that crystallised into white crystals upon refrigeration. The crude product was used without further purification.

^{1}H NMR: (600 MHz, $CDCl_3$) δ 6.83 (s, 1H), 3.63 (t, J = 6.6 Hz, 2H), 2.34 (t, J = 7.4 Hz, H), 1.68 – 1.53 (m, 4H), 1.44 – 1.35 (m, 2H).

^{13}C NMR: (151 MHz, $CDCl_3$) δ 178.78, 62.54, 34.03, 32.13, 25.27, 24.50.

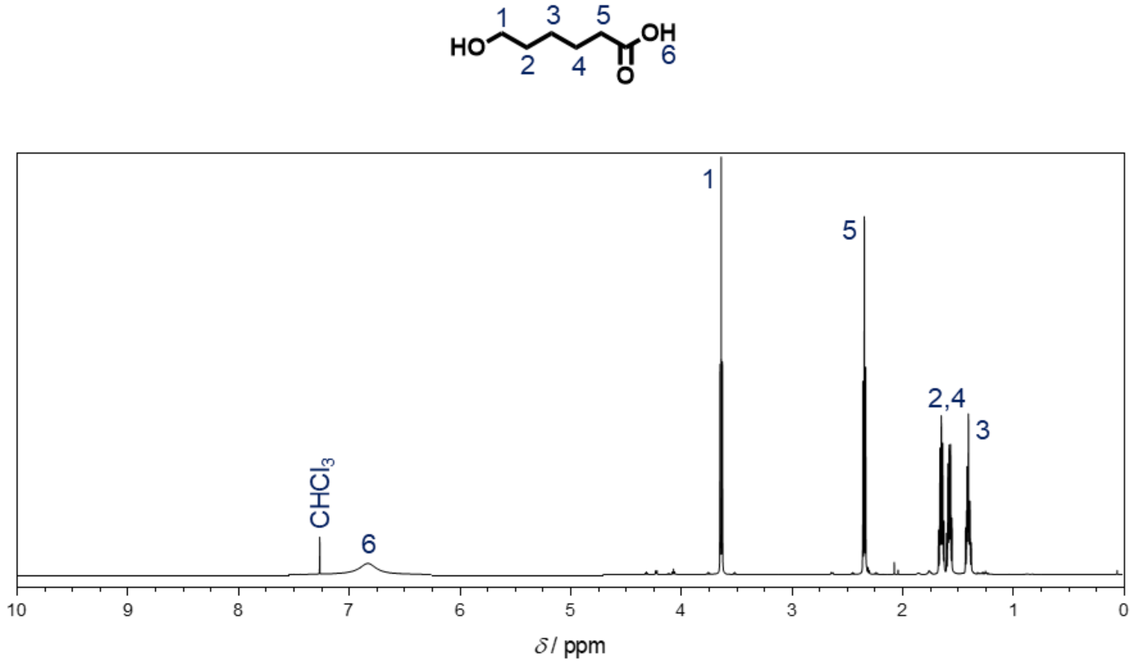

Supplementary Fig. 2. ^{1}H NMR spectrum of **(1)** in $CDCl_3$

2.3.2 Synthesis of 6-*tert*-butyldimethylsilyletherhexanoic acid (2)

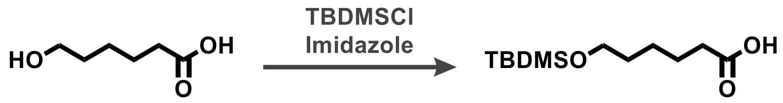

(1) **(2)**

6-*Tert*-butyldimethylsilyletherhexanoic acid was synthesised as described in the literature.[2,3] 6-Hydroxyhexanoic acid, **1**, (3.0 g, 22.7 mmol, 1.0 eq.), and imidazole (3.9 g, 57.3 mmol, 2.5 eq.) were dissolved in 10 mL anhydrous *N,N*-dimethylformamide at room temperature. The solution was cooled to 0°C, and *tert*-butyldimethylsilyl chloride (3.8 g, 25.0 mmol, 1.1 eq) was added as a solid. The solution was stirred for 5 minutes before being heated to 50°C and stirred overnight. The solution was poured into 150 mL ice/brine mixture and the aqueous phase was extracted with diethyl ether (3 x 100 mL). The organic fractions were collected, dried over sodium sulphate, and the crude product was directly adsorbed onto celite. It was purified by automated flash chromatography using a 120 g silica column eluting 10 → 40% ethyl acetate in cyclohexane. 2.6 g (48% yield) of a clear colourless oil (**2**) were isolated.

^{1}H NMR: (600 MHz, $CDCl_3$) δ 3.60 (t, *J* = 6.5 Hz, 2H), 2.34 (t, *J* = 7.5 Hz, 2H), 1.65 (tdd, *J* = 9.3, 6.6, 5.3 Hz, 2H), 1.53 (ddt, *J* = 9.4, 8.2, 6.4 Hz, 2H), 1.42 – 1.34 (m, 2H), 0.88 (s, 9H), 0.04 (s, 6H).

ESI-MS: calculated for $[C_{12}H_{26}O_3Si – H]^-$ = 245.1567; found = 245.1577

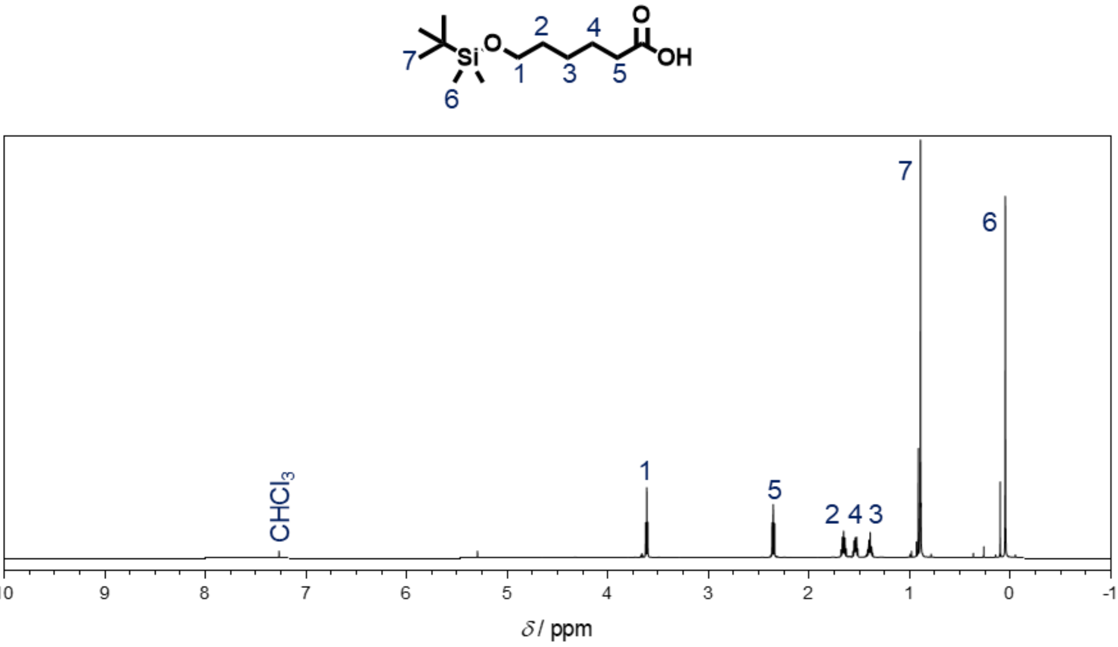

Supplementary Fig. 3. ^{1}H NMR spectrum of **(2)** in $CDCl_3$

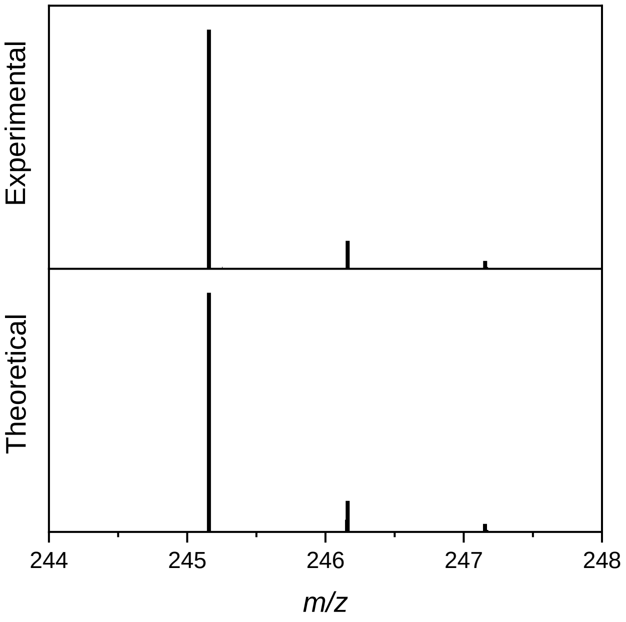

Supplementary Fig. 4. HRMS of **(2)** with experimental *m/z* (top) and calculated *m/z* for $[C_{12}H_{26}O_3Si - H]^-$ (bottom)

2.3.3 Synthesis of 1-(4-(3-hydroxypropoxy)-3-methoxyphenyl)ethan-1-one (3)

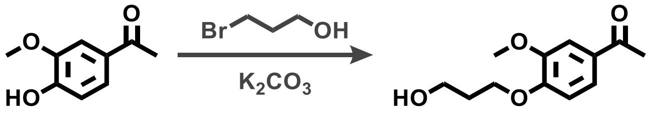

(3)

1-(4-(3-Hydroxypropoxy)-3-methoxyphenyl)ethan-1-one was synthesised as described in the literature.[4] Acetovanillone (13.5 g, 81 mmol, 1 eq.) and potassium carbonate (24.61 g, 178 mmol, 2.2 eq.) were stirred under argon in 50 mL of dry DMF for 15 minutes. 3-bromo-1-propanol (10 mL, 97 mmol, 1.2 eq) was added dropwise and the reaction was stirred at 50°C under argon overnight, at which point TLC indicated the complete consumption of acetovanillone. The reaction was poured into 800 mL of ice-brine mixture and extracted with ethyl acetate (3 x 200 mL). The organic phase was collected and dried over Na_2SO_4, before the solvent was removed in vacuo, to give a pale oil that crystalised upon further drying and refrigeration in quantitative yield.

^{1}H NMR: (400 MHz, DMSO) δ 7.61 (dd, J = 8.4, 2.1 Hz, 1H), 7.43 (d, J = 2.0 Hz, 1H), 7.06 (d, J = 8.4 Hz, 1H), 4.56 (t, J = 5.1 Hz, 1H), 4.11 (t, J = 6.4 Hz, 2H), 3.81 (s, 3H), 3.56 (td, J = 6.2, 5.1 Hz, 2H), 2.52 (s, 3H), 1.88 (p, J = 6.3 Hz, 2H).

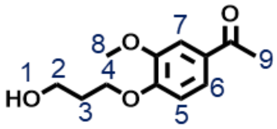

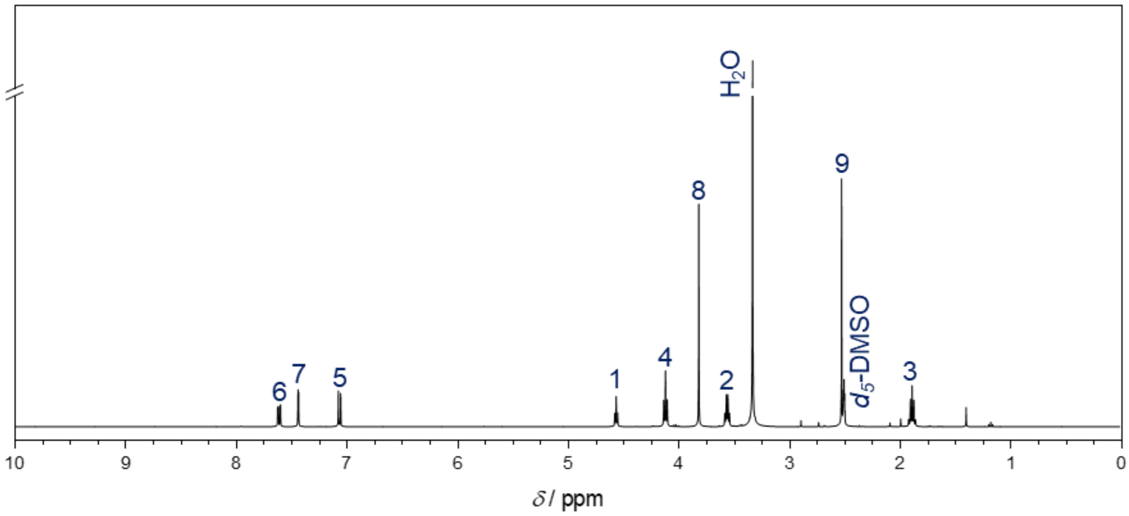

Supplementary Fig. 5. ^{1}H NMR spectrum of **(3)** in d_6-DMSO

2.3.4 Synthesis of (3-hydroxypropoxy)pyrene-chalcone (4)

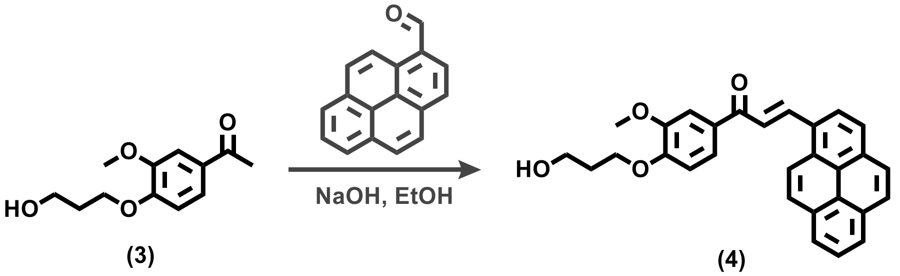

(3) **(4)**

(3-Hydroxypropoxy)pyrene-chalcone was synthesised as described in the literature.[4] **3** (5.5 g, 24 mmol, 1.0 eq.) and 1-pyrenecarboxaldehyde (6.2 g, 27 mmol, 1.1 eq.) were suspended in 65 mL ethanol. Aqueous sodium hydroxide solution (3M, 20 mL, 2.5 eq.) was added, and the solution stirred rapidly in the dark overnight. The slurry was poured into 1 L water, then 20 mL of concentrated hydrochloric acid was added and stirred for 5 minutes. The slurry was filtered and the crude product recovered as a yellow solid. The product was purified by silica gel chromatography eluting 5 → 20% acetone in toluene 7.5g of yellow solid were recovered (70% yield).

^{1}H NMR: (600 MHz, DMSO) δ 8.89 – 8.80 (m, 2H), 8.60 (d, J = 9.4 Hz, 1H), 8.39 – 8.31 (m, 4H), 8.29 – 8.22 (m, 3H), 8.12 (t, J = 7.6 Hz, 1H), 8.01 (dd, J = 8.5, 2.1 Hz, 1H), 7.72 (d, J = 2.0 Hz, 1H), 7.15 (d, J = 8.5 Hz, 1H), 4.61 (t, J = 5.1 Hz, 1H), 4.17 (t, J = 6.4 Hz, 2H), 3.91 (s, 3H), 3.60 (q, J = 6.0 Hz, 2H), 1.93 (p, J = 6.3 Hz, 2H).

ESI-MS: calculated for $[C_{29}H_{24}O_4 + H]^+$ = 437.1747; found = 437.1746

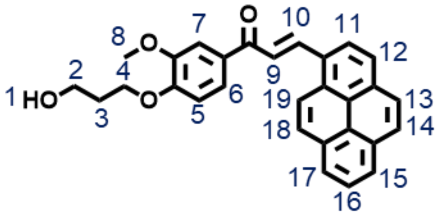

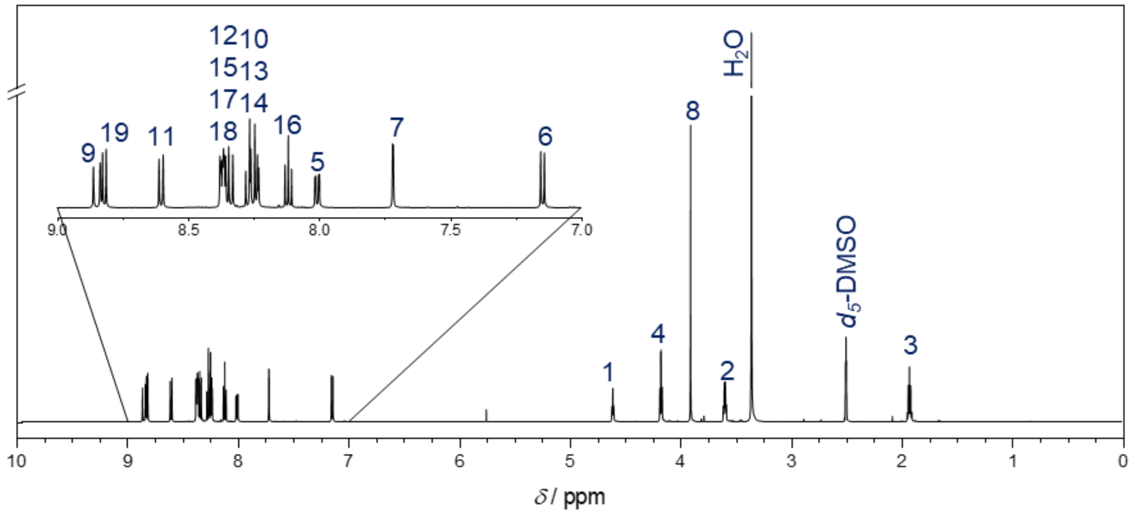

Supplementary Fig. 6. ^{1}H NMR spectrum of **(4)** in d_6-DMSO

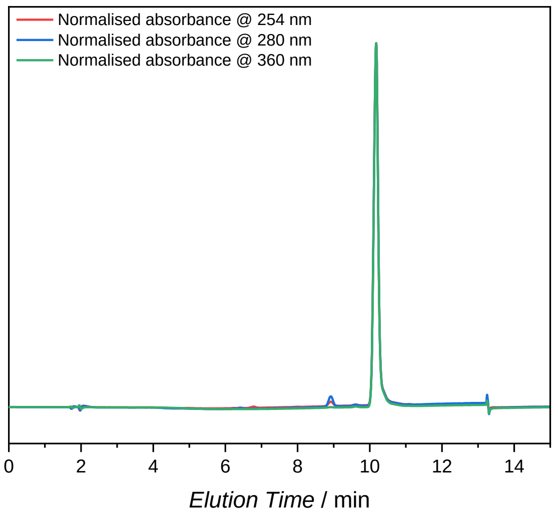

Supplementary Fig. 7. HPLC chromatographs for **(4)** showing the response from three different UV detectors

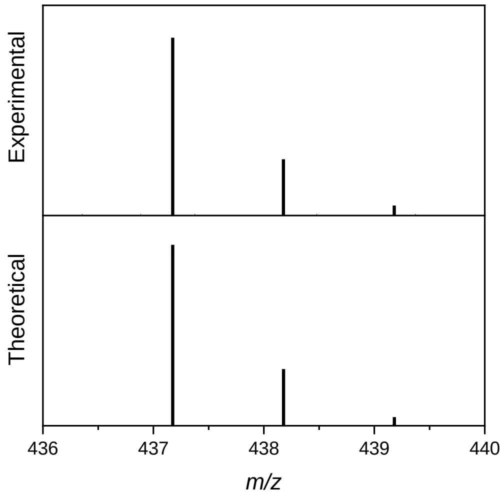

Supplementary Fig. 8. HRMS of **(4)** with experimental *m/z* (top) and calculated *m/z* for $[C_{29}H_{24}O_4 + H]^+$ (bottom)

2.3.5 Synthesis of acetonide-2,2-*bis*(methoxy) propionic acid (5)

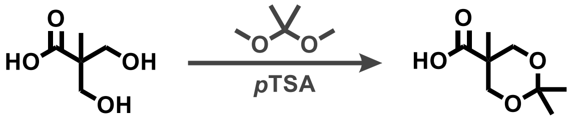

(5)

Acetonide-2,2-bis(methoxy) propionic acid was synthesised as described in the literature.[5] 2,2-bis(hydroxymethyl)propionic acid (10 g, 75 mmol, 1 eq.), acetone dimethyl acetal (13.8 mL, 112 mmol, 1.5 eq.), and *para*-toluenesulphonic acid (0.7 g, 4 mmol, 0.05 eq.) were dissolved in acetone (50 mL) and stirred for 2 h under ambient conditions. Then, triethylamine (0.6 mL, 4.4 mmol, 0.06 eq.) was added. The volatile organics were removed under reduced pressure, leaving a white solid as the crude product. The crude product was subsequently dissolved in dichloromethane (100 mL) and washed with water (2 x 20 mL), the organic fraction was dried over magnesium sulphate, and after solvent evaporation, 9.73 g (75% yield) of a white crystalline solid were recovered.

^{1}H NMR: (600 MHz, $CDCl_3$) δ 4.19 (dd, *J* = 11.9, 1.1 Hz, 2H), 3.69 – 3.64 (m, 2H), 1.42 (d, *J* = 19.3 Hz, 6H), 1.21 (s, 3H).

ESI-MS: calculated for $[C_8H_{14}O_4 + H]^+$ = 175.0965; found = 175.0963

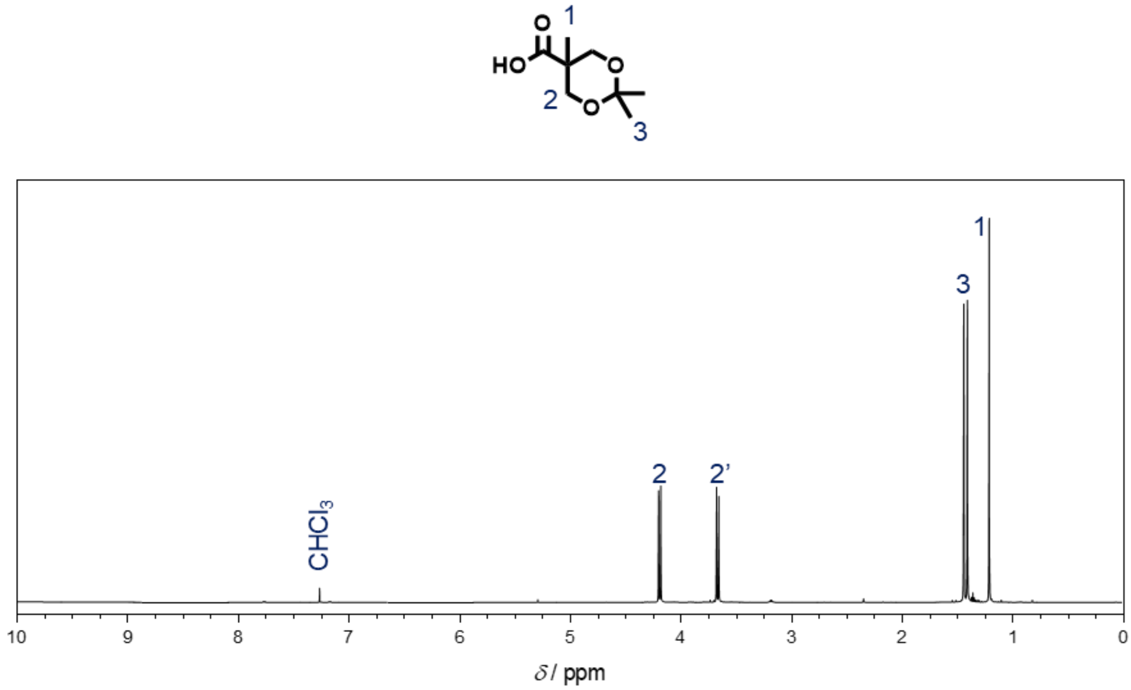

Supplementary Fig. 9. ^{1}H NMR spectrum of **(5)** in $CDCl_3$

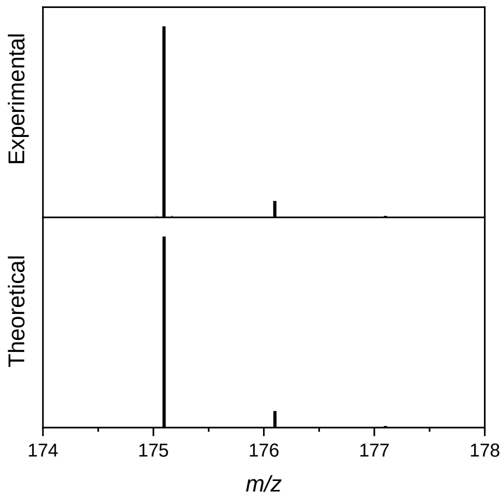

Supplementary Fig. 10. HRMS of **(5)** with experimental *m/z* (top) and calculated *m/z* for $[C_8H_{14}O_4 + H]^+$ (bottom)

2.3.6 Synthesis of dihydroxypyrene chalcone (6)

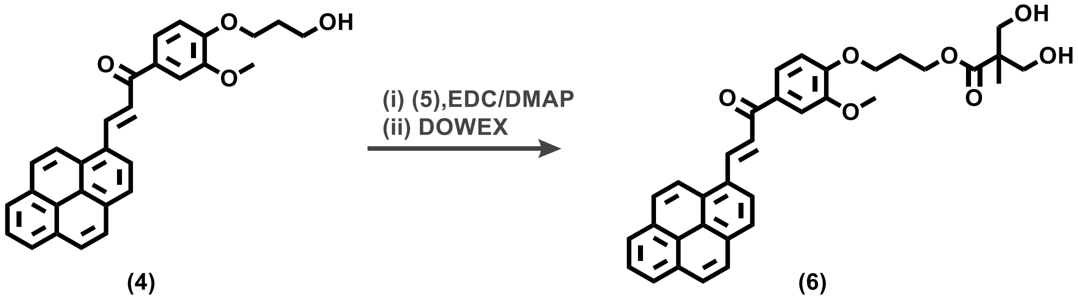

(4) **(6)**

(4) (500 mg, 1 eq.), **(5)** (798 mg, 4 eq.), 1-ethyl-3-(3-dimethylaminopropyl)carbodiimide (1.76 g, 8 eq.), and 4-dimethylaminopyridine (139 mg, 1 eq.) were dissolved in 40 mL of dichloromethane under argon and stirred overnight. When TLC indicated that full consumption of the starting material had been achieved, the organic reaction mixture was

extracted with 1 M hydrochloric acid (2 x 20 mL) and brine (1 x 20 mL). The organic phase was dried over sodium sulphate and the dichloromethane removed to give a yellow solid that was subsequently suspended in a slurry of DOWEX resin (500 mg) in methanol (50 mL) and was stirred for 4 h at 50 °C. The slurry was filtered and washed with dichloromethane until all yellow solid had passed through the filter. The crude solution was extracted with $NaHCO_3$ (2 x 20 mL), and brine (1 x 20 mL). The organic phase was dried over sodium sulphate and the solvent removed under reduced pressure. Finally, **(6)** was isolated by column chromatography eluting 1 → 5 % methanol in dichloromethane to yield 512 mg (81% yield) as a yellow solid.

^{1}H NMR: (400 MHz, DMSO) δ 8.83 (d, *J* = 15.3 Hz, 1H), 8.77 (d, *J* = 8.2 Hz, 1H), 8.55 (d, *J* = 9.4 Hz, 1H), 8.36 – 8.15 (m, 7H), 8.08 (t, *J* = 7.6 Hz, 1H), 7.97 (dd, *J* = 8.5, 2.0 Hz, 1H), 7.71 (d, *J* = 2.0 Hz, 1H), 7.11 (d, *J* = 8.5 Hz, 1H), 4.72 (t, *J* = 5.5 Hz, 2H), 4.19 (dt, *J* = 17.1, 6.3 Hz, 4H), 3.90 (s, 3H), 3.52 (ddd, *J* = 39.6, 10.4, 5.5 Hz, 4H), 2.09 (p, *J* = 6.3 Hz, 2H), 1.10 (s, 3H).

^{13}C NMR (151 MHz, DMSO) δ 187.64, 175.23, 152.95, 149.42, 139.24, 132.75, 131.30, 131.15, 130.64, 129.98, 129.18, 129.00, 128.87, 127.82, 127.03, 126.60, 126.35, 125.67, 125.41, 124.56, 124.53, 124.25, 123.93, 122.72, 112.25, 111.57, 65.65, 64.49, 61.03, 56.15, 50.82, 28.61, 17.41.

ESI-MS: calculated for $[C_{34}H_{32}O_7 + H]^+$ = 553.2221; found = 553.2224

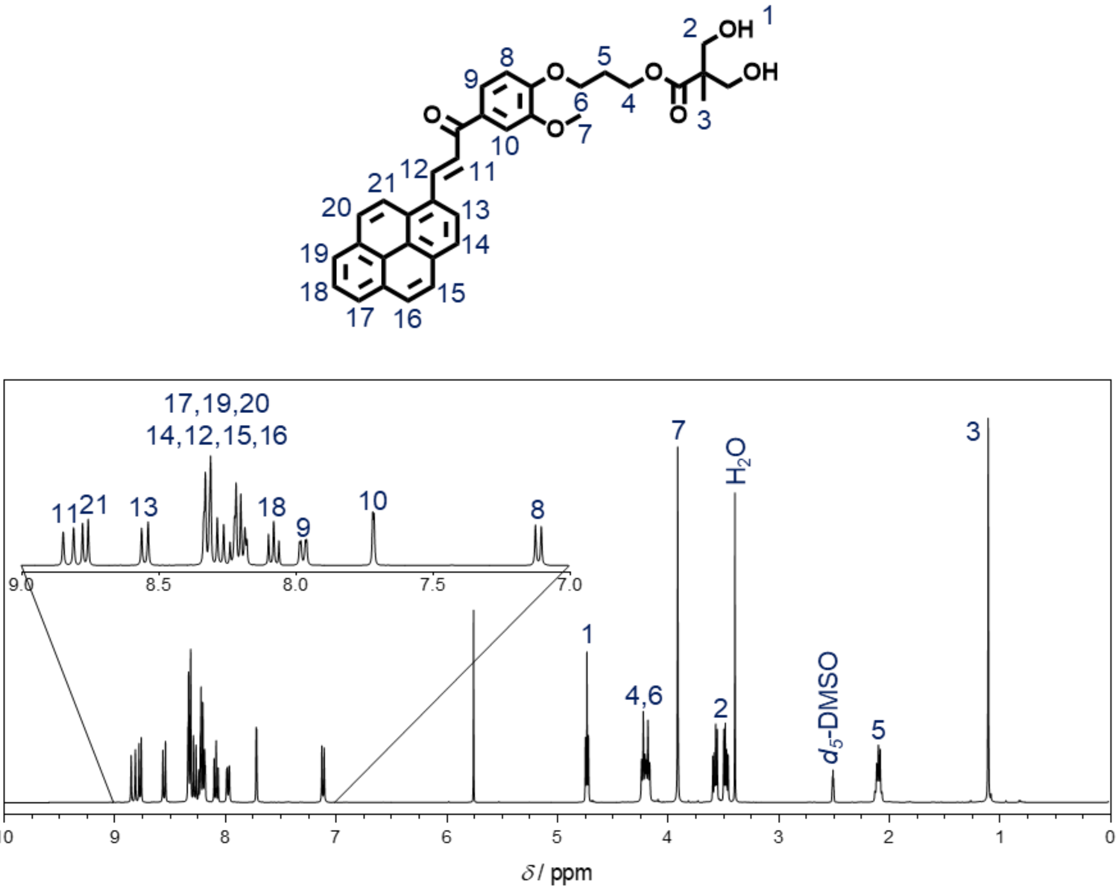

Supplementary Fig. 11. ^{1}H NMR spectrum of **(6)** in d_6-DMSO

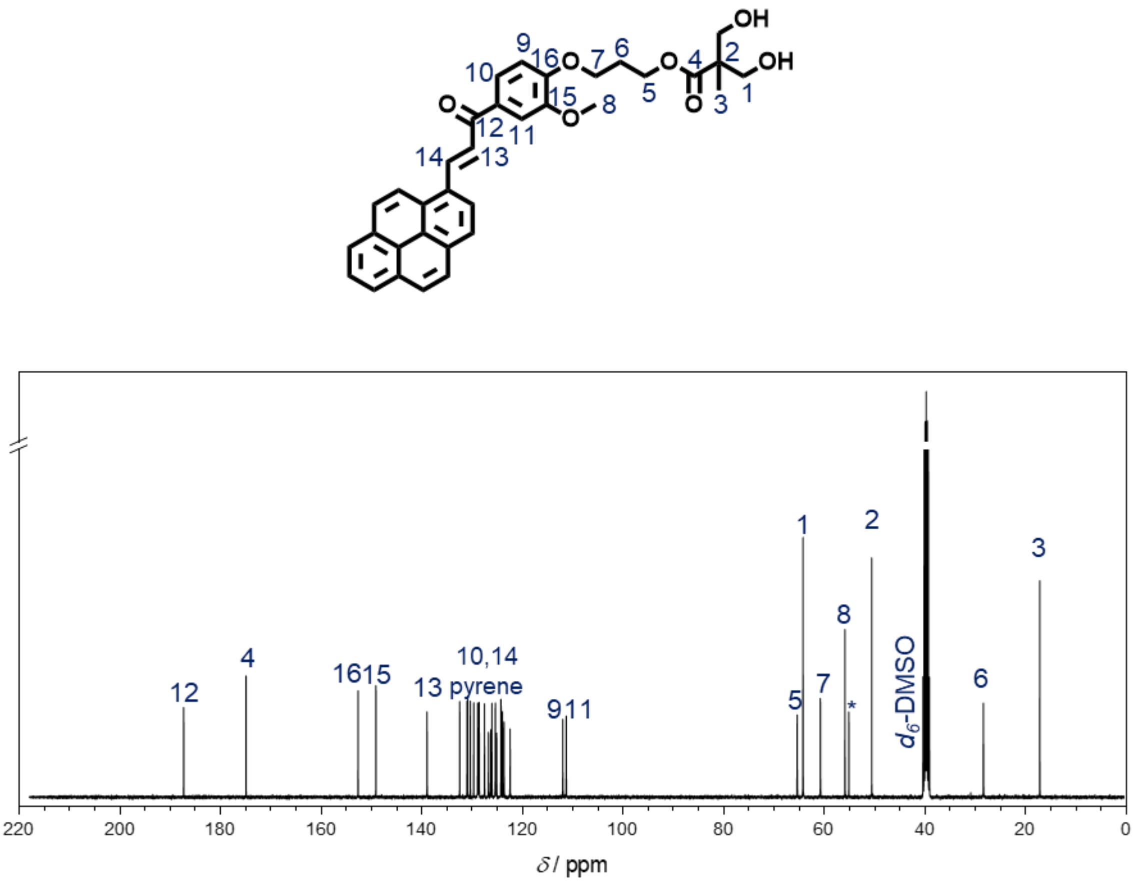

Supplementary Fig. 12. ^{13}C NMR spectrum of **(6)** in *d*$_6$-DMSO, * denotes a resonance from residual dichloromethane.

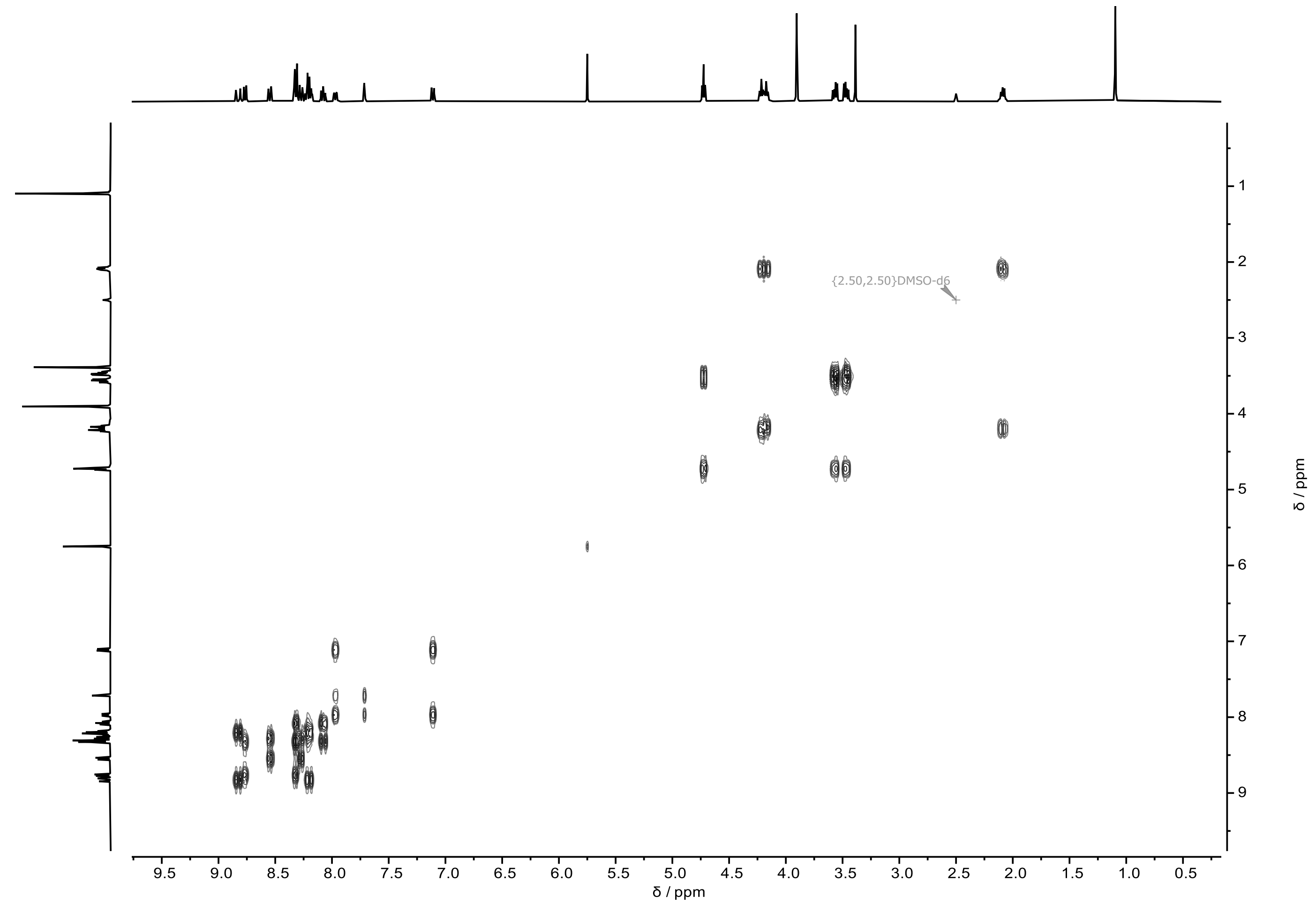

Supplementary Fig. 13. COSY NMR spectrum of **(6)** in *d*$_6$-DMSO

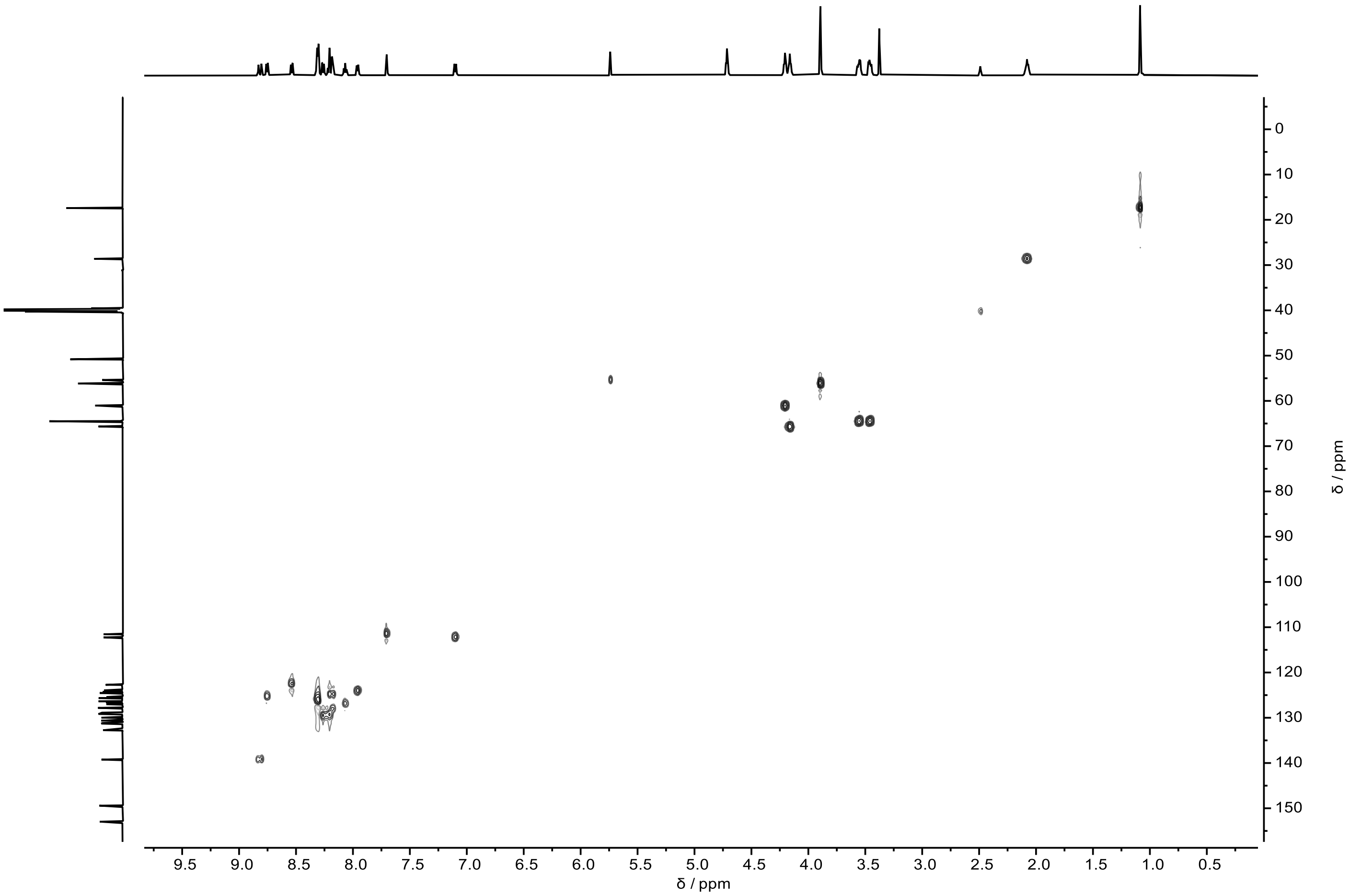

Supplementary Fig. 14. HSQC NMR spectrum of **(6)** in d_6-DMSO.

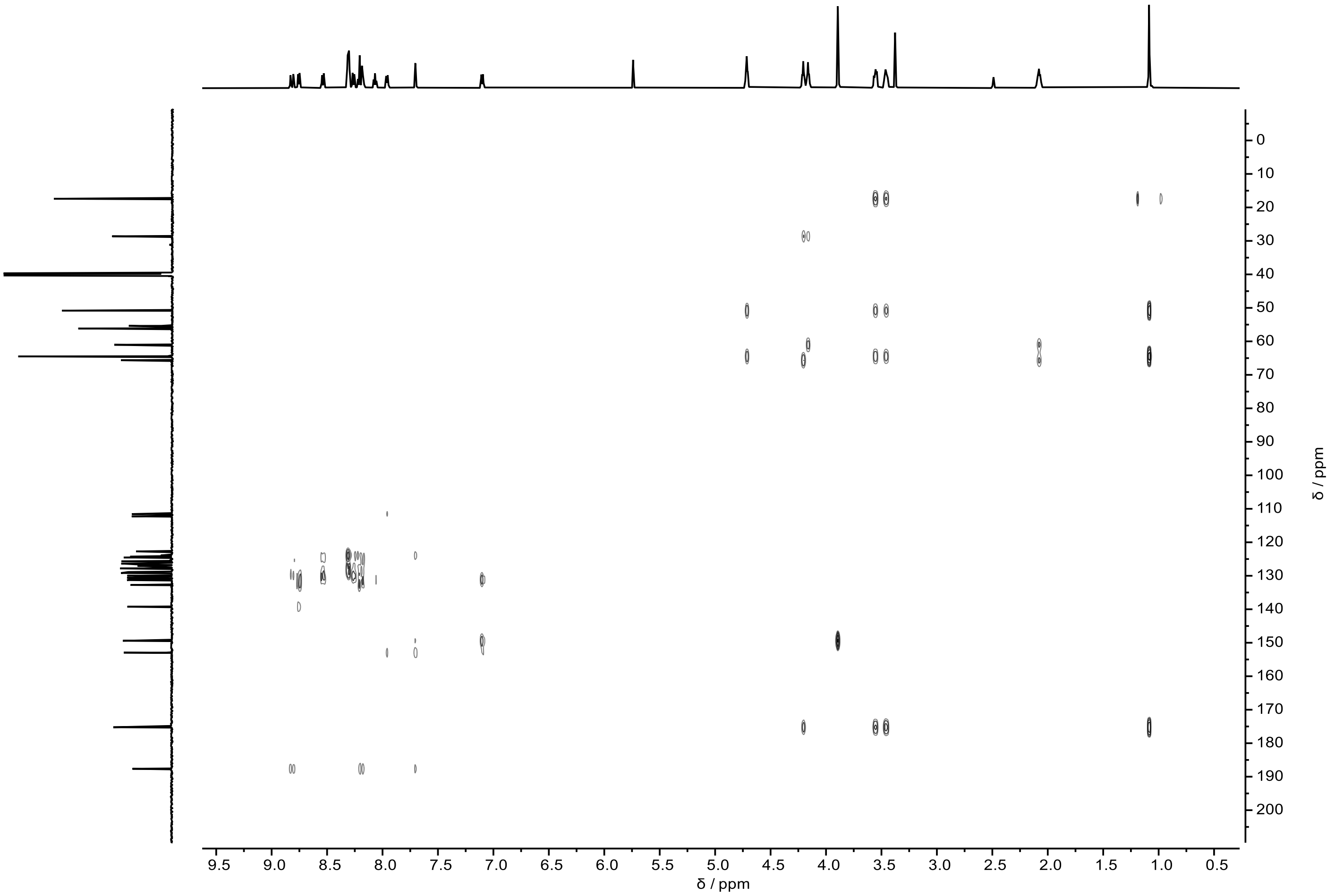

Supplementary Fig. 15. HMBC NMR spectrum of **(6)** in d_6-DMSO.

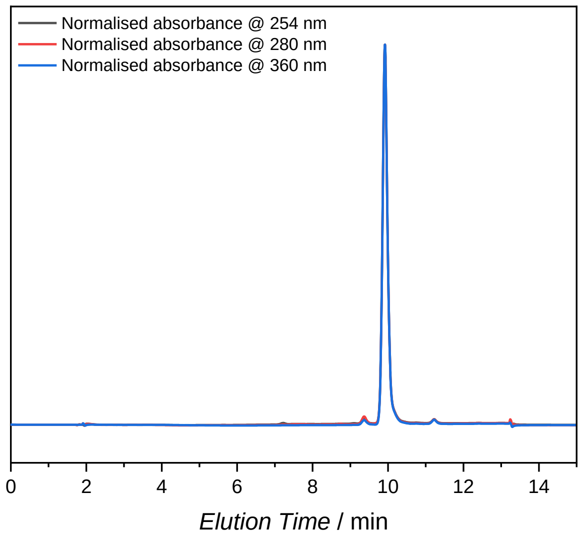

Supplementary Fig. 16. HPLC chromatographs for **(6)** showing the response from three different UV detectors

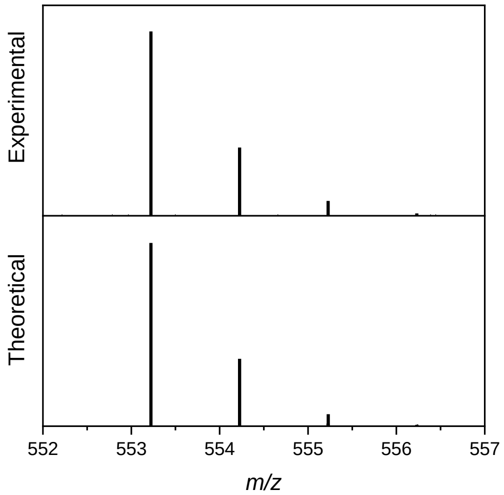

Supplementary Fig. 17. HRMS of **(6)** with experimental *m/z* (top) and calculated *m/z* for $[C_{34}H_{32}O_7 + H]^+$ (bottom)

2.3.7 Synthesis of ethyl 4-(4-acetyl-2-methoxyphenoxy)butanoate (7)

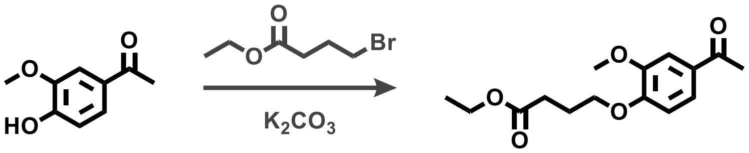

(7)

Ethyl 4-(4-acetyl-2-methoxyphenoxy)butanoate was synthesised by an adaptation of literature procedures.[4] Acetovanillone (5 g, 30 mmol, 1.0 eq.), ethyl 4-bromobutyrate (5.17 mL, 36 mmol, 1.2 eq.), and potassium carbonate (10.4 g, 75 mmol, 2.5 eq.) were combined in anhydrous N,N-dimethylformamide (50 mL) under argon. The suspension was stirred rapidly at 50°C for 24 h. The crude reaction mixture was cooled to room temperature, and adsorbed directly onto celite that was subsequently purified by column chromatography in a 10% solution of ethyl acetate in cyclohexane. 7.76 g (92 % yield) of a white crystalline solid was obtained.

^{1}H NMR (600 MHz, DMSO) δ 7.58 (dd, *J* = 8.4, 2.1 Hz, 1H), 7.44 (d, *J* = 2.1 Hz, 1H), 7.03 (d, *J* = 8.4 Hz, 1H), 4.09 – 4.03 (m, 4H), 3.82 (s, 3H), 2.51 (s, 3H), 2.46 (t, *J* = 7.3 Hz, 2H), 2.04 – 1.96 (m, 2H), 1.17 (t, *J* = 7.1 Hz, 3H).

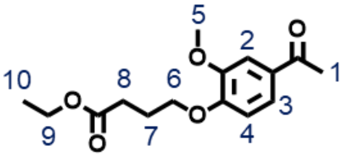

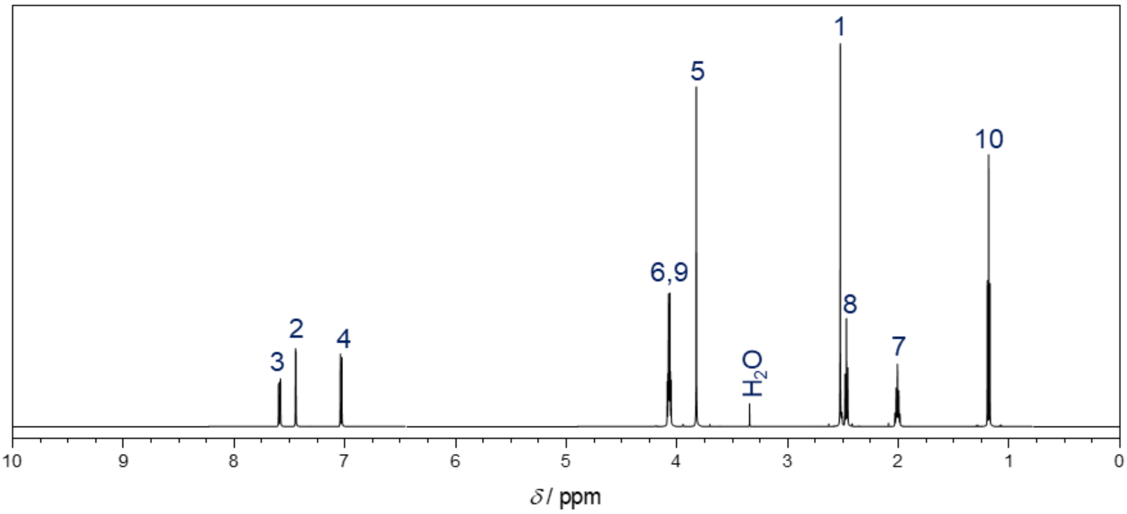

Supplementary Fig. 18. ^{1}H NMR spectrum of **(7)** in d_6-DMSO

2.3.8 Synthesis of (butyric acid)pyrene chalcone (8)

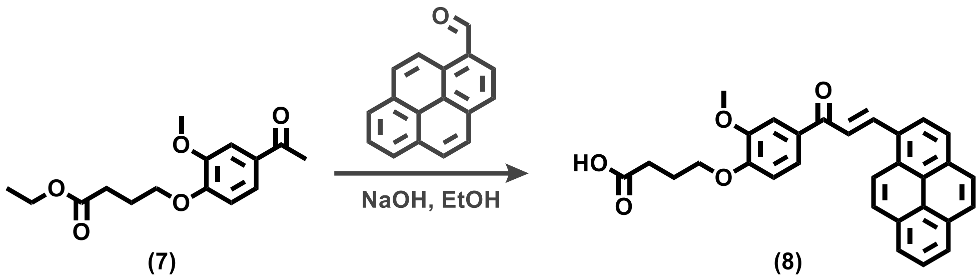

(butyric acid)pyrene chalcone was synthesised by an adaptation of literature procedures.[4] Ethyl 4-(4-acetyl-2-methoxyphenoxy)butanoate (4.47 g, 15.95 mmol, 1.0 eq.) and 1-pyrenecarboxaldehyde (4.04 g, 17.54 mmol, 1.1 eq.) were suspended by sonication in a mixture of 3 M sodium hydroxide (13.29 mL, 39.87 mmol, 2.5 eq.) and ethanol (45 mL).The suspension was stirred rapidly in the dark, under argon for 48 h at room temperature. The slurry was subsequently poured into milli-Q water (900 mL) to which concentrated hydrochloric acid (7.5 mL) was added and stirred for 30 minutes. The solid was isolated by filtration, and the cake was dried under reduced pressure at 40°C for a week. The crude product was purified by recrystallization from toluene, using drops of methanol as an antisolvent. 5.26 g of a yellow powder was obtained (71 % yield).

^{1}H NMR (600 MHz, DMSO) δ 8.85 (d, *J* = 15.2 Hz, 1H), 8.82 (d, *J* = 8.2 Hz, 1H), 8.61 (d, *J* = 9.4 Hz, 1H), 8.39 – 8.34 (m, 4H), 8.29 – 8.23 (m, 3H), 8.12 (t, *J* = 7.6 Hz, 1H), 8.00 (dd, *J* = 8.5, 2.1 Hz, 1H), 7.72 (d, *J* = 2.0 Hz, 1H), 7.15 (d, *J* = 8.5 Hz, 1H), 4.12 (t, *J* = 6.5 Hz, 2H), 3.91 (s, 3H), 2.42 (t, *J* = 7.3 Hz, 2H), 2.00 (p, *J* = 6.9 Hz, 2H).

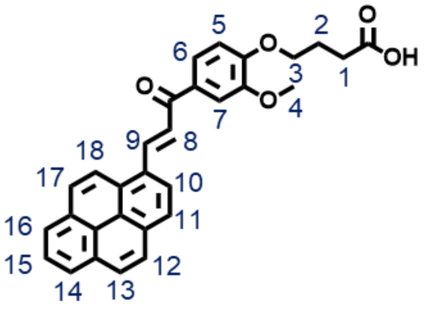

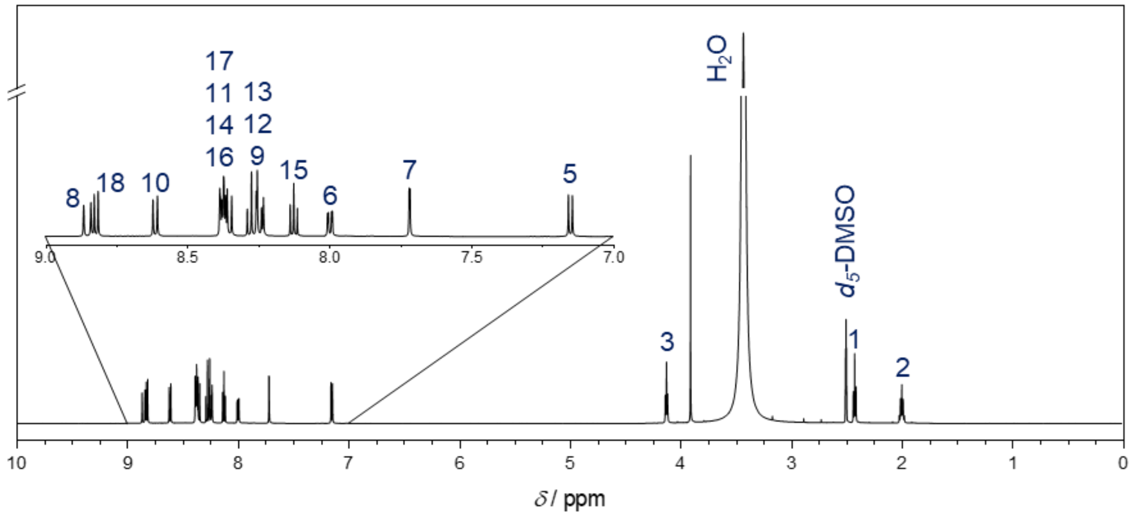

Supplementary Fig. 19. ^{1}H NMR spectrum of **(8)** in d_6-DMSO

2.4 Iterative sequential growth procedures

2.4.1 General procedure for addition steps

The diol (either **(6)** for the first step, or the deprotected oligomer for subsequent steps, 1 eq.), **(2)** (4 eq.), 1-ethyl-3-(3-dimethylaminopropyl)carbodiimide (8 eq.) and 4-dimethylaminopyridine (1 eq.) are dissolved in the minimum amount of dichloromethane under argon and stirred for 4 h, or until TLC indicates complete consumption of the starting material. The dichloromethane solution is subsequently extracted with equal volumes of 1 M hydrochloric acid twice and then

brine. The organic layer is dried over sodium sulphate and the solvent removed under reduced pressure. The crude mixture is used in the next deprotection step without further purification.

2.4.2 General procedure for deprotection steps

The chain-end protected oligomer (1 eq.) is dissolved in a 1 M tetrabutylammonium fluoride solution in THF (10 eq.) under argon. The solution is stirred for 2 h, or until TLC shows complete consumption of the starting material. Then, it is dissolved in a 3:1 volume:volume mixture of diethyl ether:dichloromethane and extracted with an equal volume of saturated ammonium chloride solution twice, followed by saturated sodium bicarbonate solution twice, and finally a single wash with brine. The organic phase is dried over sodium sulphate and the solvent removed under reduced pressure. The crude product is used in the next addition step without further purification or purified by silica gel chromatography eluting 5 → 20 % acetone in toluene before chain-end functionalisation with **(8)**.

2.5 Chemical structures of T0-5

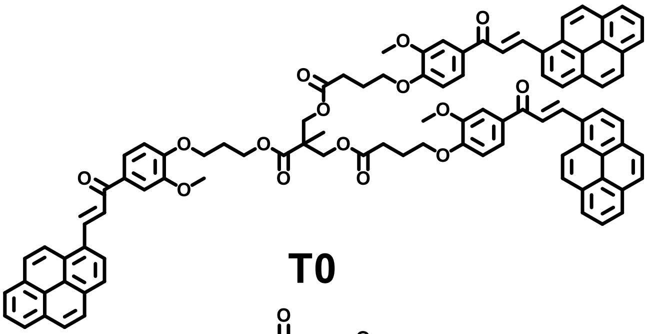

T0

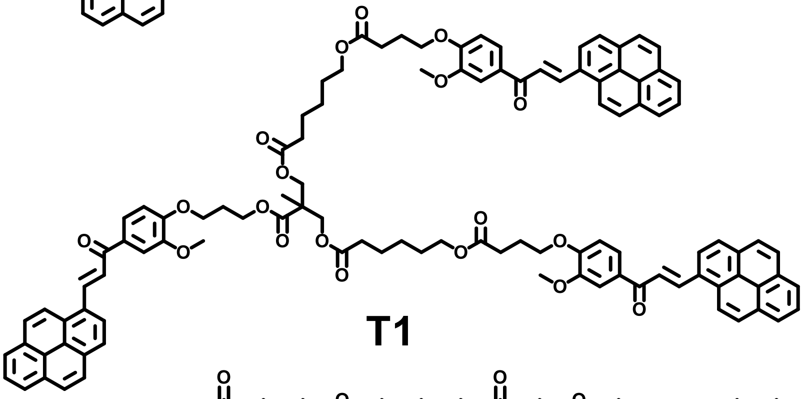

T1

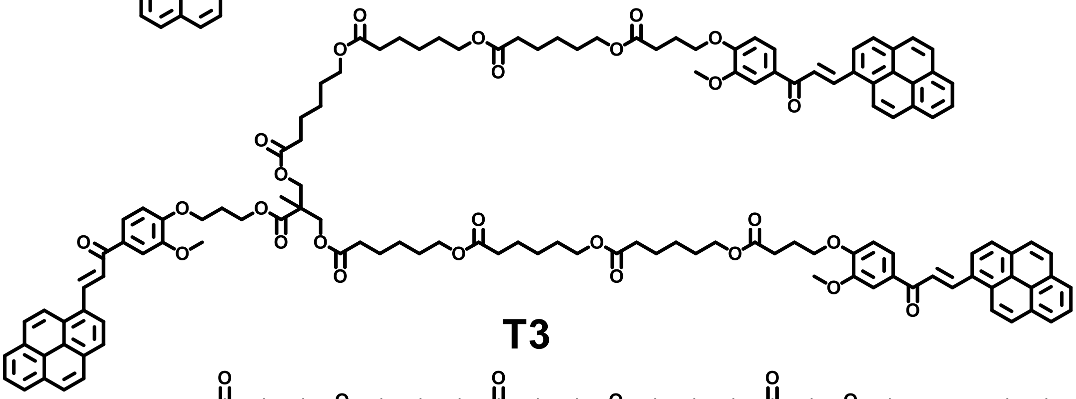

T3

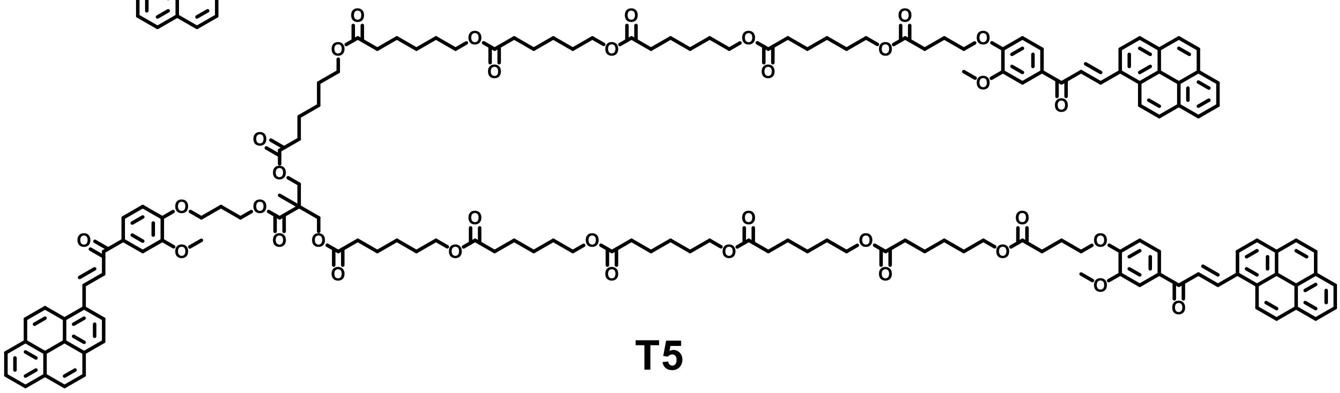

T5

2.6 Synthesis and characterisation of final molecules

2.6.1 General procedure for chain-end functionalisation with PyChal

The chain-end deprotected oligomer of required length (1.0 eq.), **8** (3.0 eq.), EDC (6 eq.), and DMAP (0.5 eq.) were all dissolved in the minimum amount of dry DMF under argon and stirred overnight. When TLC indicated complete consumption of the starting material, and the mono-substituted product, the reaction mixture was adsorbed onto celite and purified by silica gel chromatography eluting 2 → 10 % acetone in toluene. All products were yellow solids.

2.6.2 NMR characterisation of each macromolecule

2.6.2.1 T0

^{1}H NMR (600 MHz, $CDCl_3$) δ 8.85 (dd, *J* = 15.3, 7.8 Hz, 3H), 8.40 (dd, *J* = 11.5, 9.3 Hz, 3H), 8.26 (dd, *J* = 8.1, 4.5 Hz, 3H), 8.11 (d, *J* = 7.6 Hz, 3H), 8.08 – 7.98 (m, 12H), 7.97 – 7.86 (m, 6H), 7.73 – 7.65 (m, 9H), 6.90 (t, *J* = 8.4 Hz, 3H), 4.37 (t, *J* = 6.1 Hz, 2H), 4.30 (q, *J* = 11.1 Hz, 4H), 4.14 (t, *J* = 6.2 Hz, 2H), 4.08 (t, *J* = 6.2 Hz, 4H), 3.97 (s, 3H), 3.95 (s, 6H), 2.55 (t, *J* = 7.2 Hz, 4H), 2.22 (p, *J* = 6.2 Hz, 2H), 2.16 (p, *J* = 6.7 Hz, 4H), 1.28 (s, 3H).

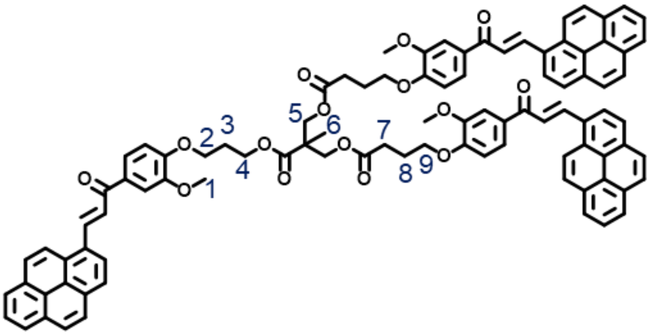

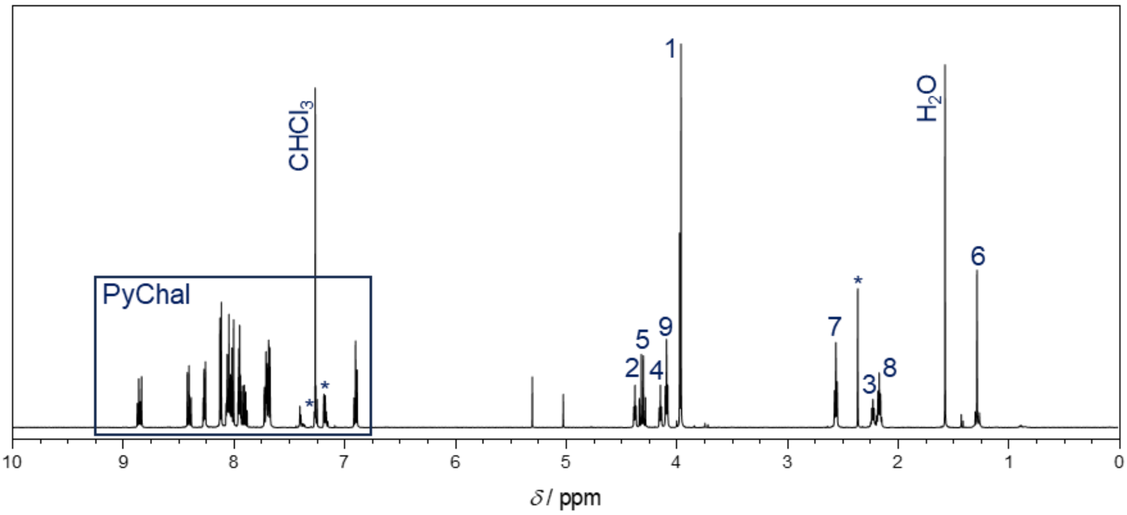

Supplementary Fig. 20 ^{1}H NMR spectrum of **T0** in $CDCl_3$, * denotes resonances from residual toluene.

^{13}C NMR (151 MHz, $CDCl_3$) δ 188.30, 188.24, 172.86, 172.63, 152.68, 149.69, 149.66, 140.72, 140.63, 132.84, 131.89, 131.70, 131.33, 130.75, 130.69, 130.28, 129.18, 128.90, 128.81, 128.64, 128.62, 128.37, 127.35, 127.30, 126.30, 126.03, 125.88, 125.44, 125.03, 124.96, 124.62, 124.15, 123.46, 123.33, 123.17, 123.11, 122.65, 122.57, 111.48, 111.36, 67.76, 65.64, 65.48, 62.18, 56.22, 56.19, 46.56, 30.57, 28.65, 24.44, 18.06.

Supplementary Fig. 21

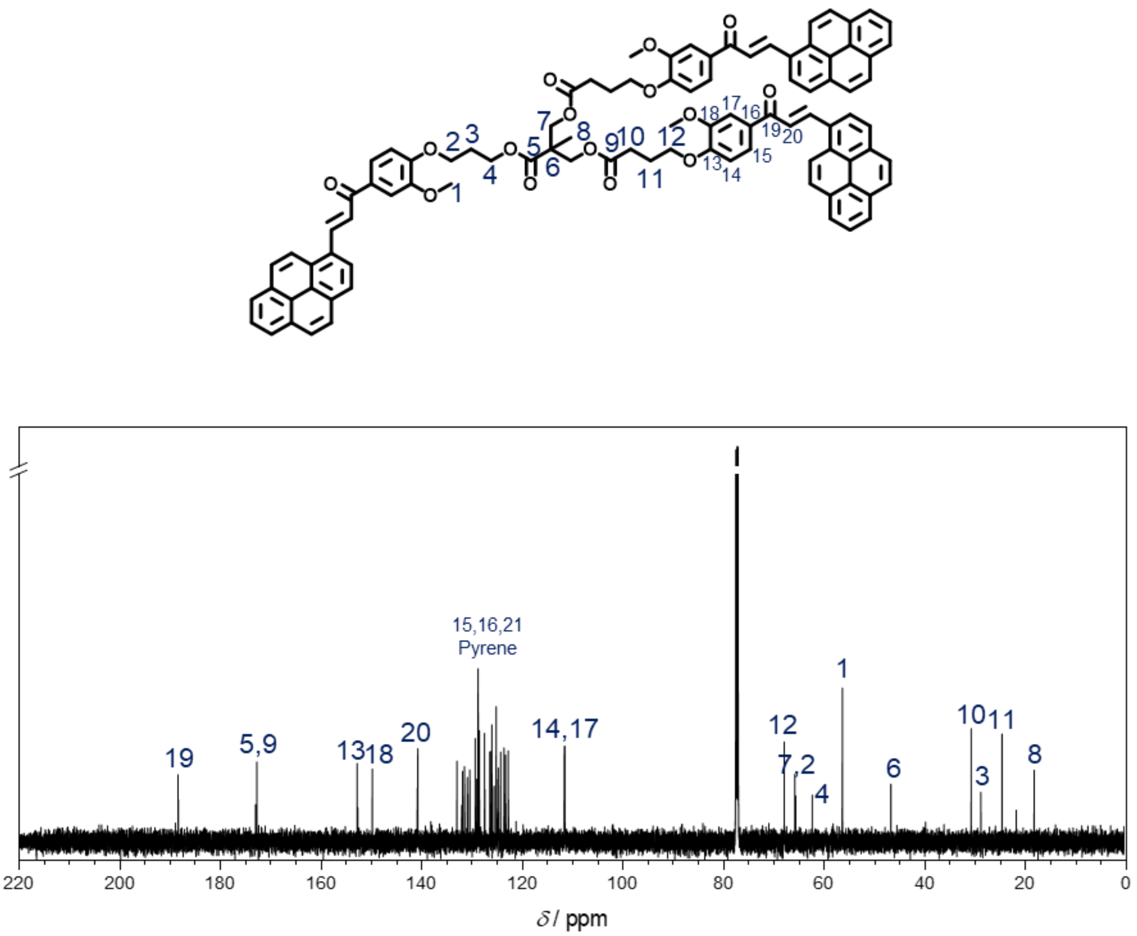

Supplementary Fig. 22. ^{13}C NMR of **T0** in $CDCl_3$.

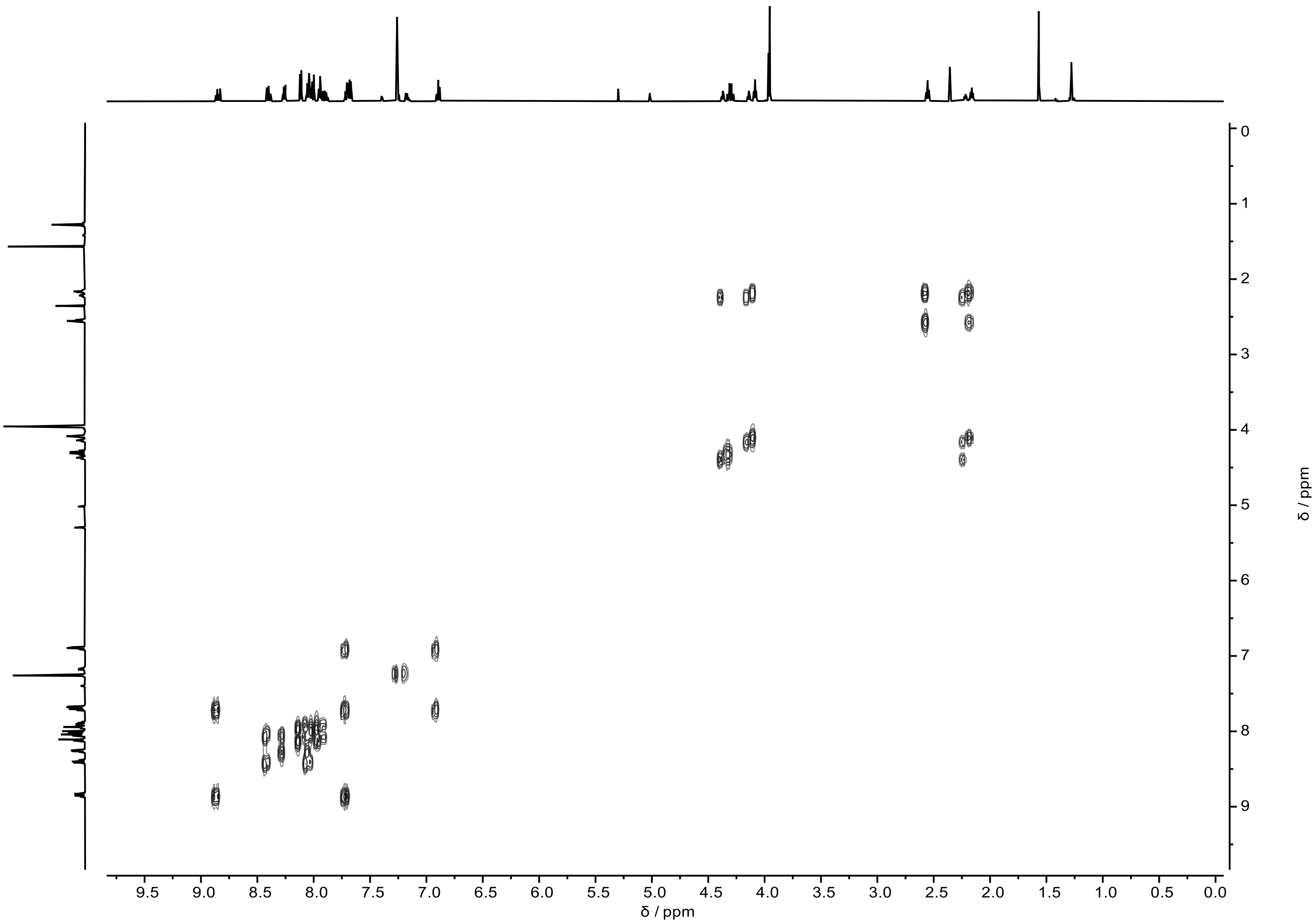

Supplementary Fig. 23. COSY NMR spectrum of **T0** in $CDCl_3$

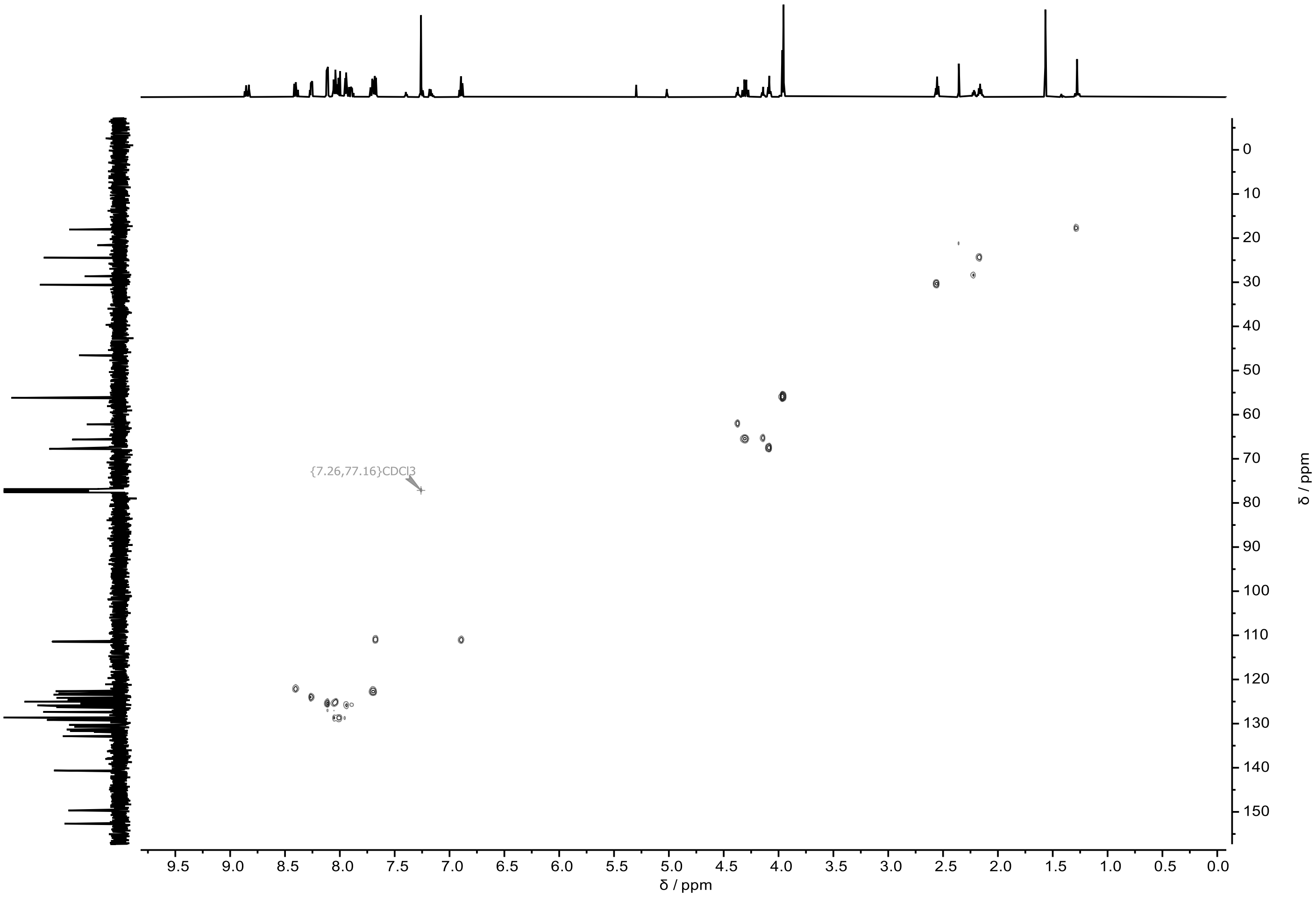

Supplementary Fig. 24. HSQC NMR spectrum of **T0** in $CDCl_3$.

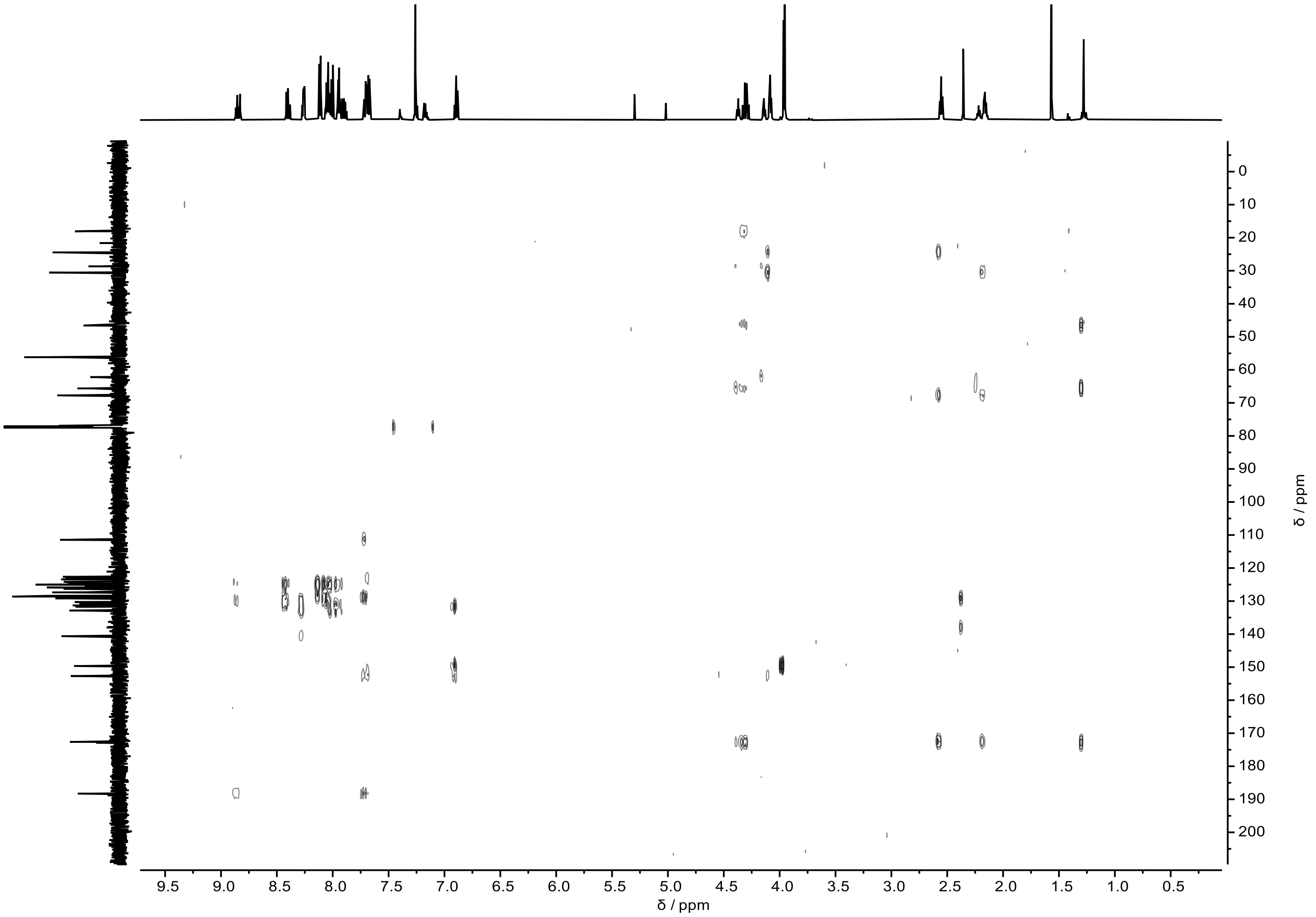

Supplementary Fig. 25. HMBC NMR spectrum of **T0** in $CDCl_3$.

2.6.2.2 T1

^{1}H NMR (600 MHz, $CDCl_3$) δ 8.92 (dd, *J* = 15.3, 6.5 Hz, 3H), 8.50 (dd, *J* = 9.3, 4.9 Hz, 3H), 8.36 (dd, *J* = 8.1, 3.0 Hz, 3H), 8.21 – 7.94 (m, 21H), 7.84 – 7.67 (m, 9H), 6.91 (dd, *J* = 10.4, 8.4 Hz, 3H), 4.36 (t, *J* = 6.2 Hz, 2H), 4.28 – 4.19 (m, 4H), 4.15 (t, *J* = 6.3 Hz, 2H), 4.12 (t, *J* = 6.3 Hz, 4H), 4.07 (t, *J* = 6.6 Hz, 4H), 3.96 (d, *J* = 4.7 Hz, 9H), 2.54 (t, *J* = 7.3 Hz, 4H), 2.28 (t, *J* = 7.5 Hz, 4H), 2.26 – 2.13 (m, 6H), 1.62 – 1.57 (m, 8H), 1.38 – 1.31 (m, 4H), 1.25 (s, 3H).

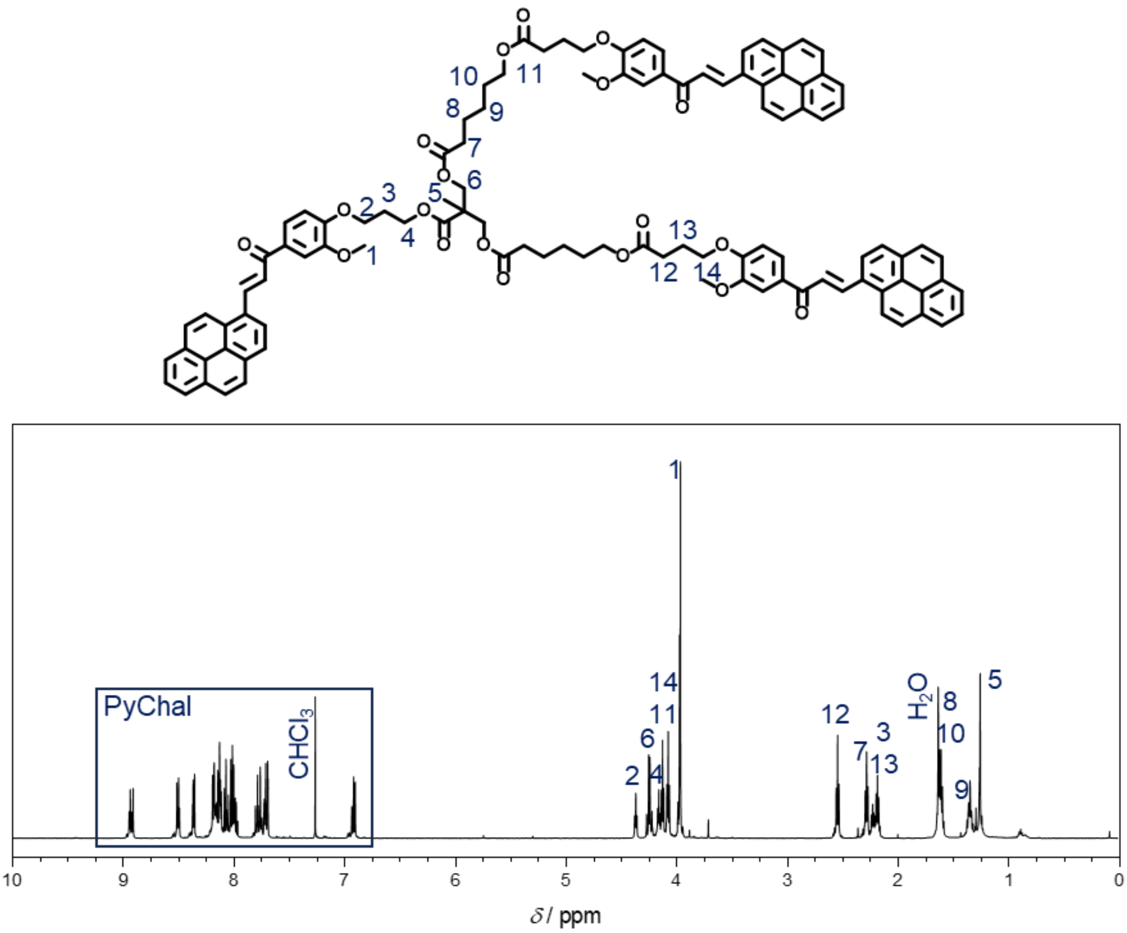

Supplementary Fig.26. ^{1}H NMR spectrum of **T1** in $CDCl_3$.

^{13}C NMR (151 MHz, $CDCl_3$) δ 188.41, 188.31, 173.17, 172.99, 172.80, 152.76, 152.59, 149.71, 149.67, 140.82, 140.69, 132.91, 132.88, 131.89, 131.62, 131.40, 131.37, 130.83, 130.80, 130.35, 130.33, 129.08, 128.99, 128.74, 128.71, 127.42, 127.39, 126.39, 126.12, 126.09, 125.95, 125.10, 125.05, 125.04, 124.70, 124.67, 124.28, 124.26, 123.80, 123.63, 123.17, 123.09, 122.76, 122.70, 111.52, 111.46, 111.39, 67.95, 65.51, 65.40, 64.42, 62.15, 56.22, 46.54, 33.98, 30.69, 28.61, 28.42, 25.59, 24.58, 24.47, 17.95.

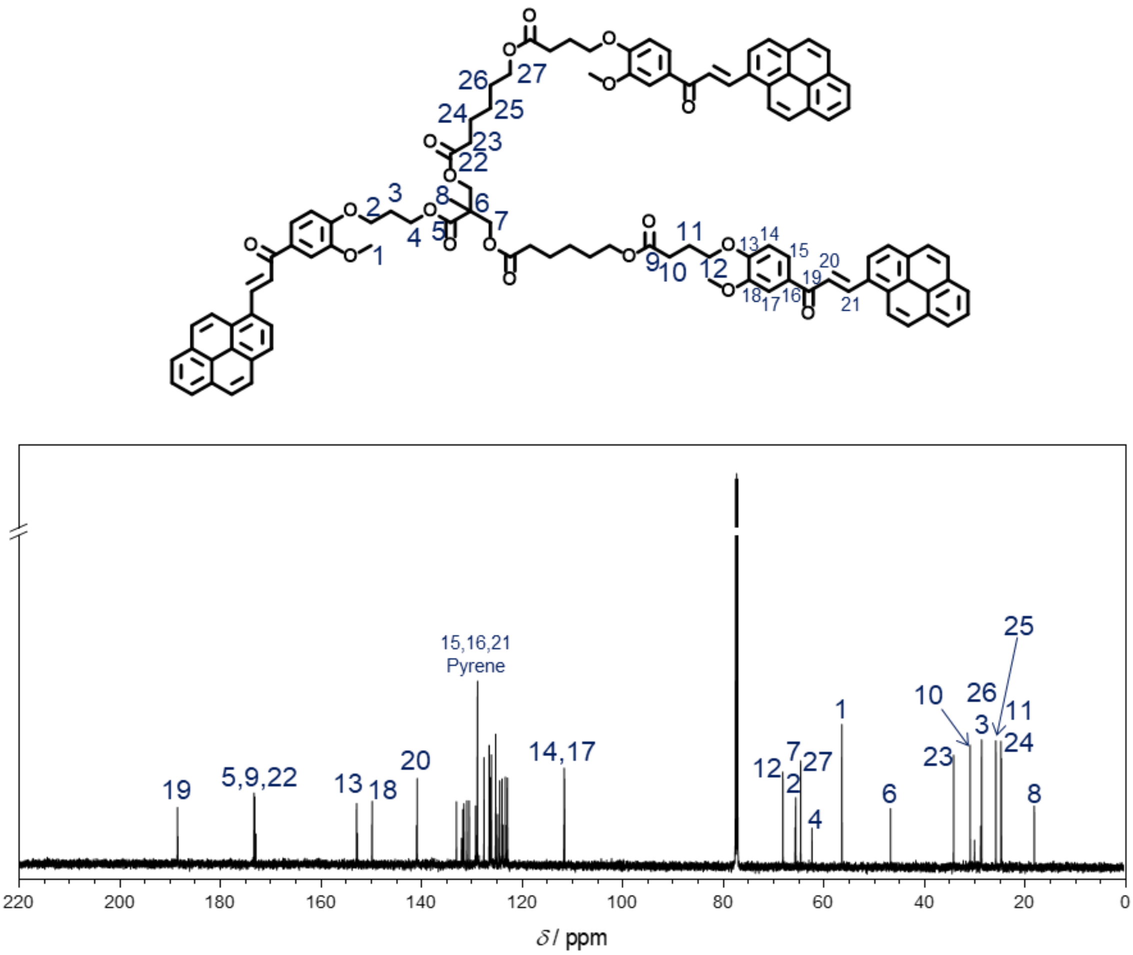

Supplementary Fig.27. ^{13}C NMR of **T1** in $CDCl_3$.

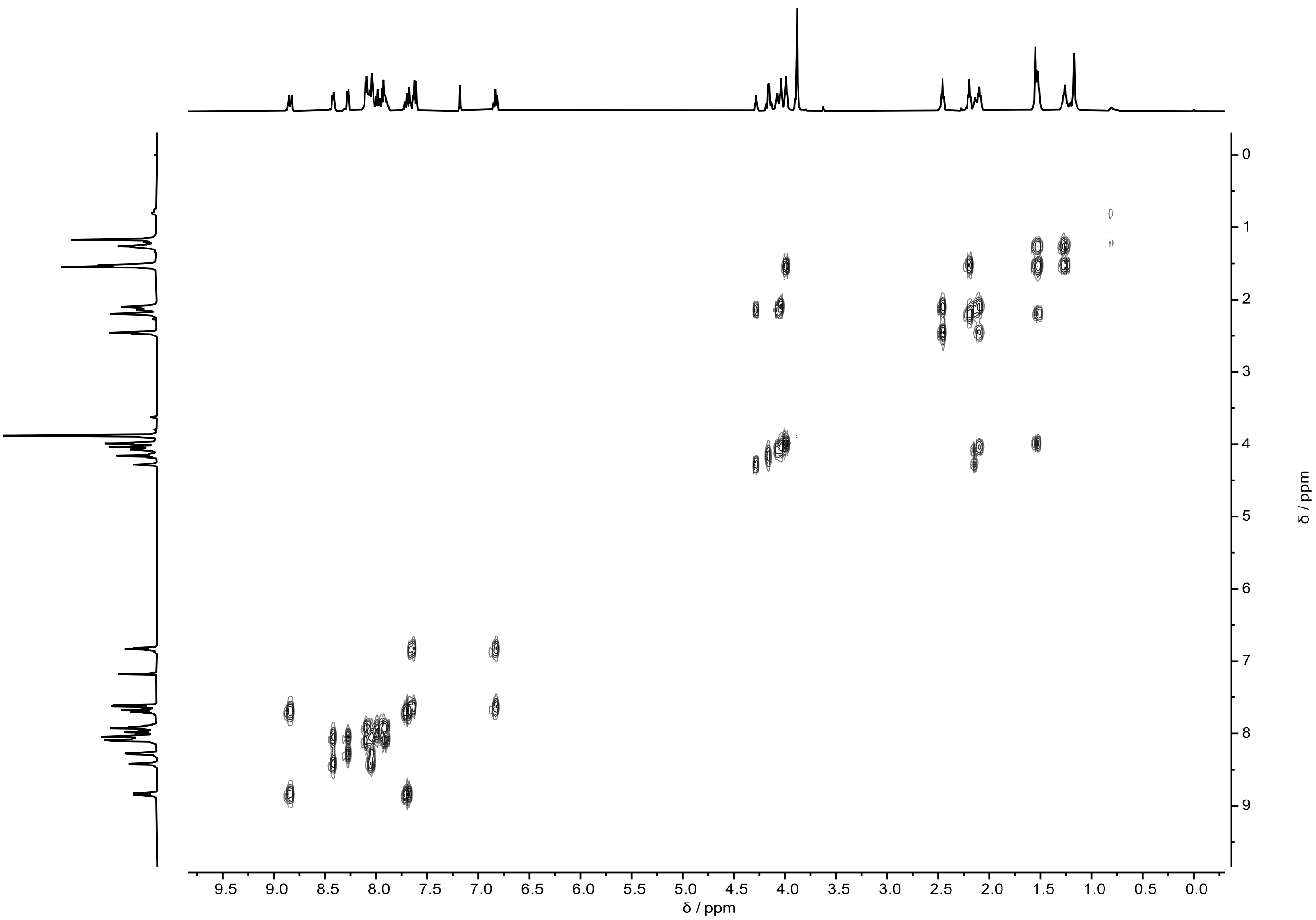

Supplementary Fig.28. COSY NMR of **T1** in $CDCl_3$.

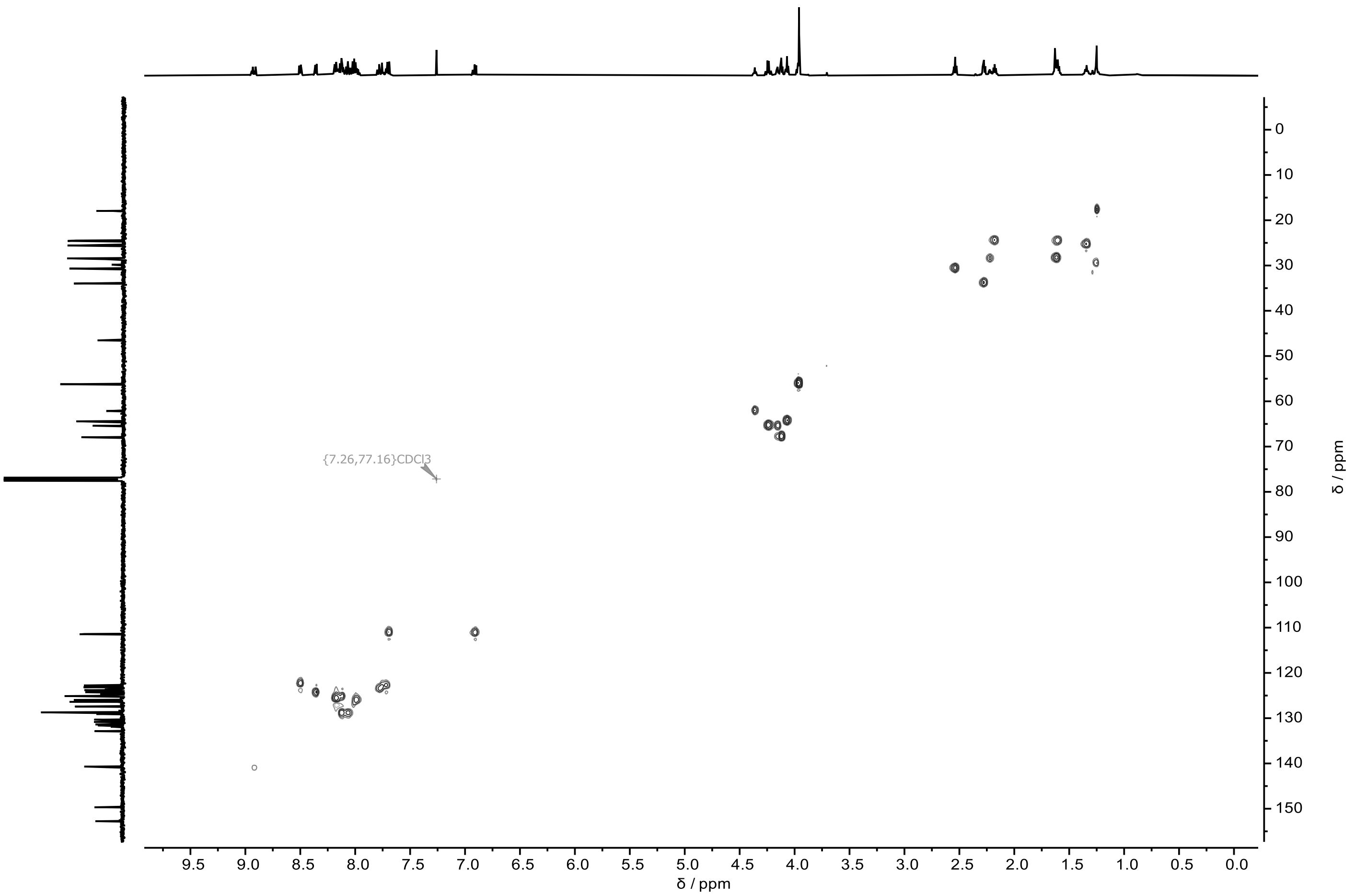

Supplementary Fig.29. HSQC NMR spectrum of **T1** in $CDCl_3$.

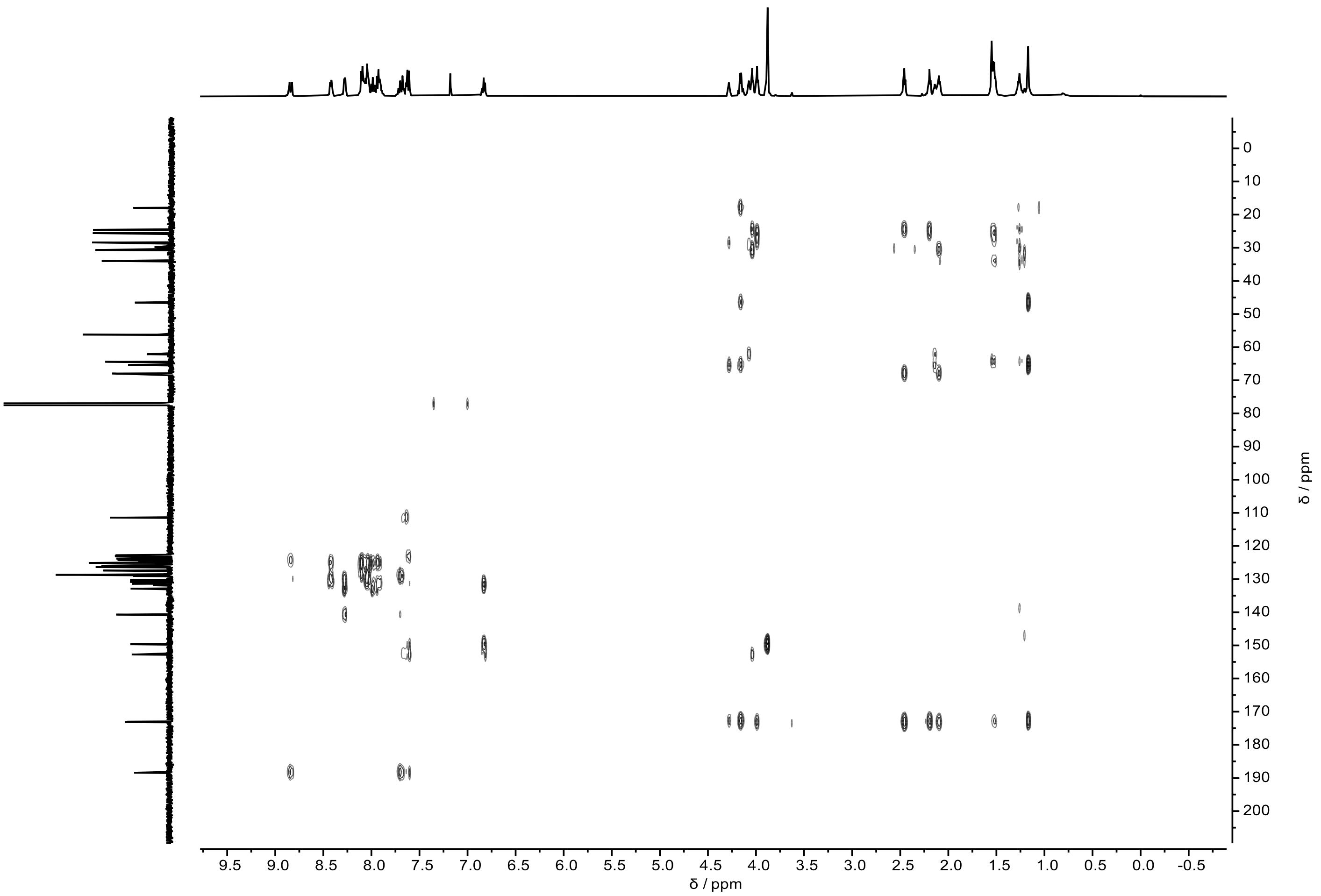

Supplementary Fig. 30. HMBC NMR spectrum of **T1** in $CDCl_3$.

2.6.2.3 T3

^{1}H NMR (600 MHz, $CDCl_3$) δ 8.95 (dd, *J* = 15.3, 4.6 Hz, 3H), 8.54 (dd, *J* = 7.6, 3.3 Hz, 3H), 8.44 – 8.37 (m, 3H), 8.27 – 7.96 (m, 21H), 7.85 – 7.73 (m, 6H), 7.71 (t, *J* = 2.7 Hz, 3H), 6.95 (dd, *J* = 8.4, 5.3 Hz, 3H), 4.37 (t, *J* = 6.2 Hz, 2H), 4.27 – 4.20 (m, 4H), 4.20 – 4.13 (m, 6H), 4.12 – 4.00 (m, 12H), 3.98 (d, *J* = 2.5 Hz, 9H), 2.56 (t, *J* = 7.1 Hz, 4H), 2.33 – 2.17 (m, 18H), 1.62 (ddq, *J* = 29.2, 15.3, 6.8 Hz, 24H), 1.40 – 1.29 (m, 12H), 1.25 (d, *J* = 4.3 Hz, 3H).

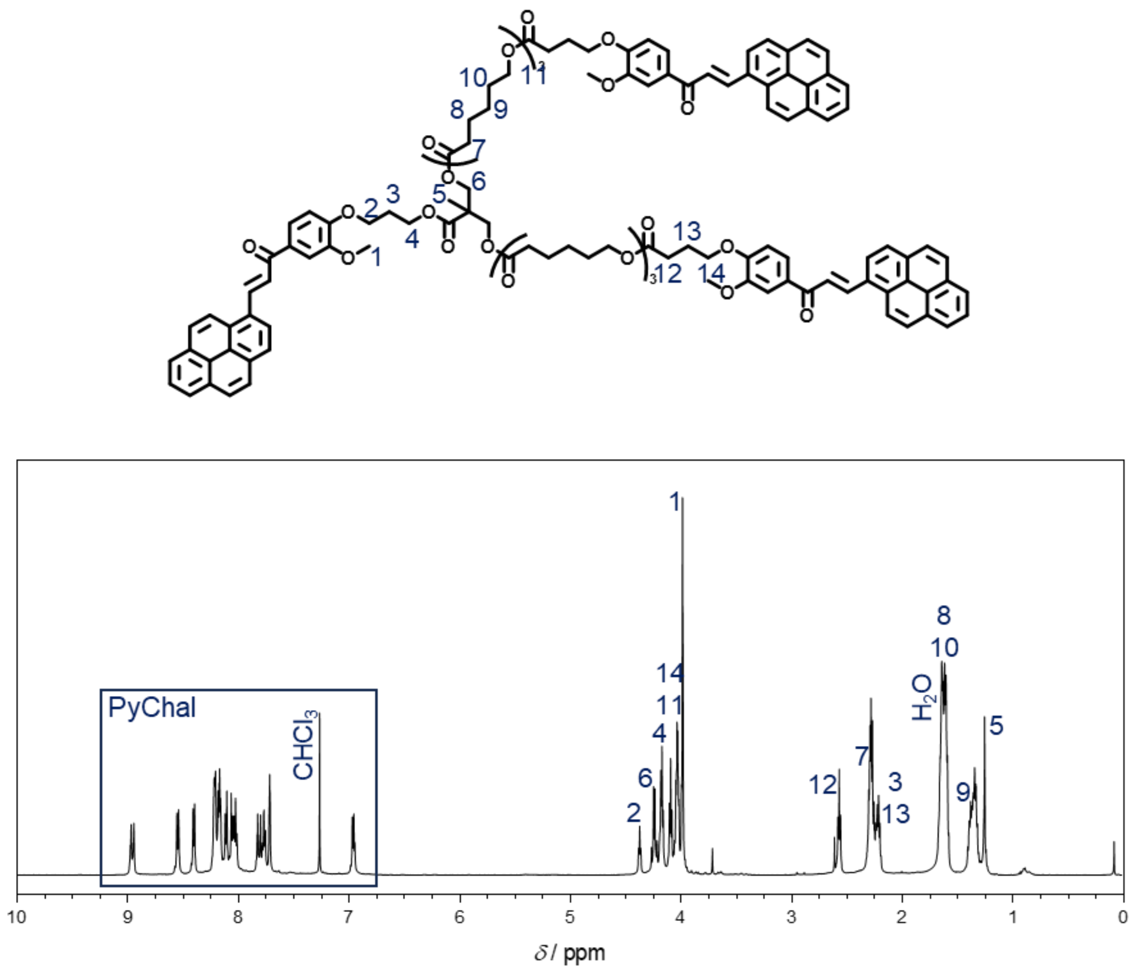

Supplementary Fig. 31. ^{1}H NMR spectrum of **T3** in $CDCl_3$.

^{13}C NMR (151 MHz, $CDCl_3$) δ 188.47, 188.37, 173.65, 173.63, 173.21, 173.00, 172.78, 152.80, 152.60, 149.74, 149.72, 140.88, 140.76, 132.95, 132.91, 131.91, 131.66, 131.44, 130.87, 130.85, 130.37, 129.16, 129.07, 128.79, 128.76, 128.75, 127.46, 127.44, 126.44, 126.43, 126.16, 126.13, 126.01, 125.99, 125.15, 125.11, 124.75, 124.73, 124.34, 124.31, 123.92, 123.75, 123.19, 123.11, 122.81, 122.77, 111.55, 111.51, 111.47, 111.44, 67.99, 65.54, 65.39, 64.51, 64.26, 64.18, 62.15, 56.26, 56.22, 46.54, 34.23, 34.20, 33.99, 30.72, 28.62, 28.46, 28.45, 25.66, 25.63, 25.58, 24.70, 24.67, 24.60, 24.58, 24.49, 17.93.

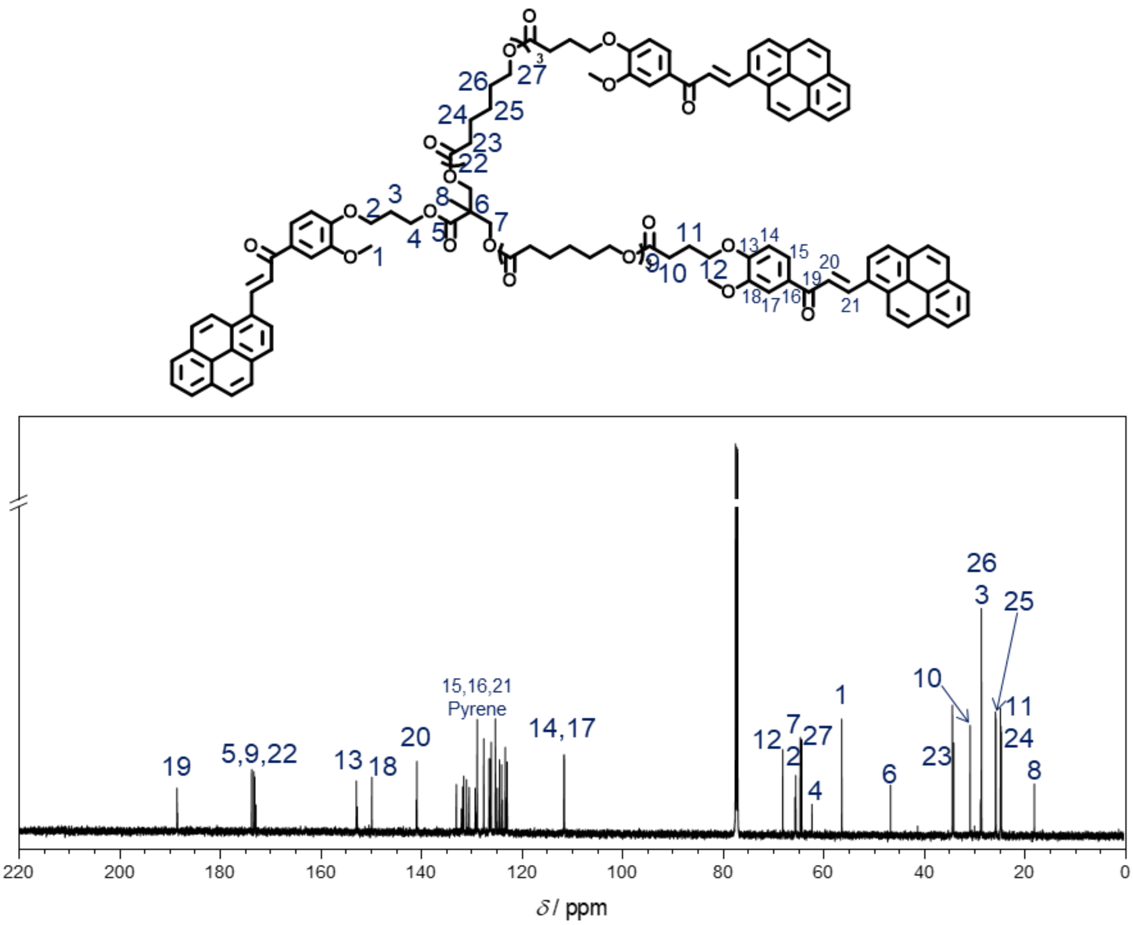

Supplementary Fig. 32. ^{13}C NMR spectrum of **T3** in $CDCl_3$.

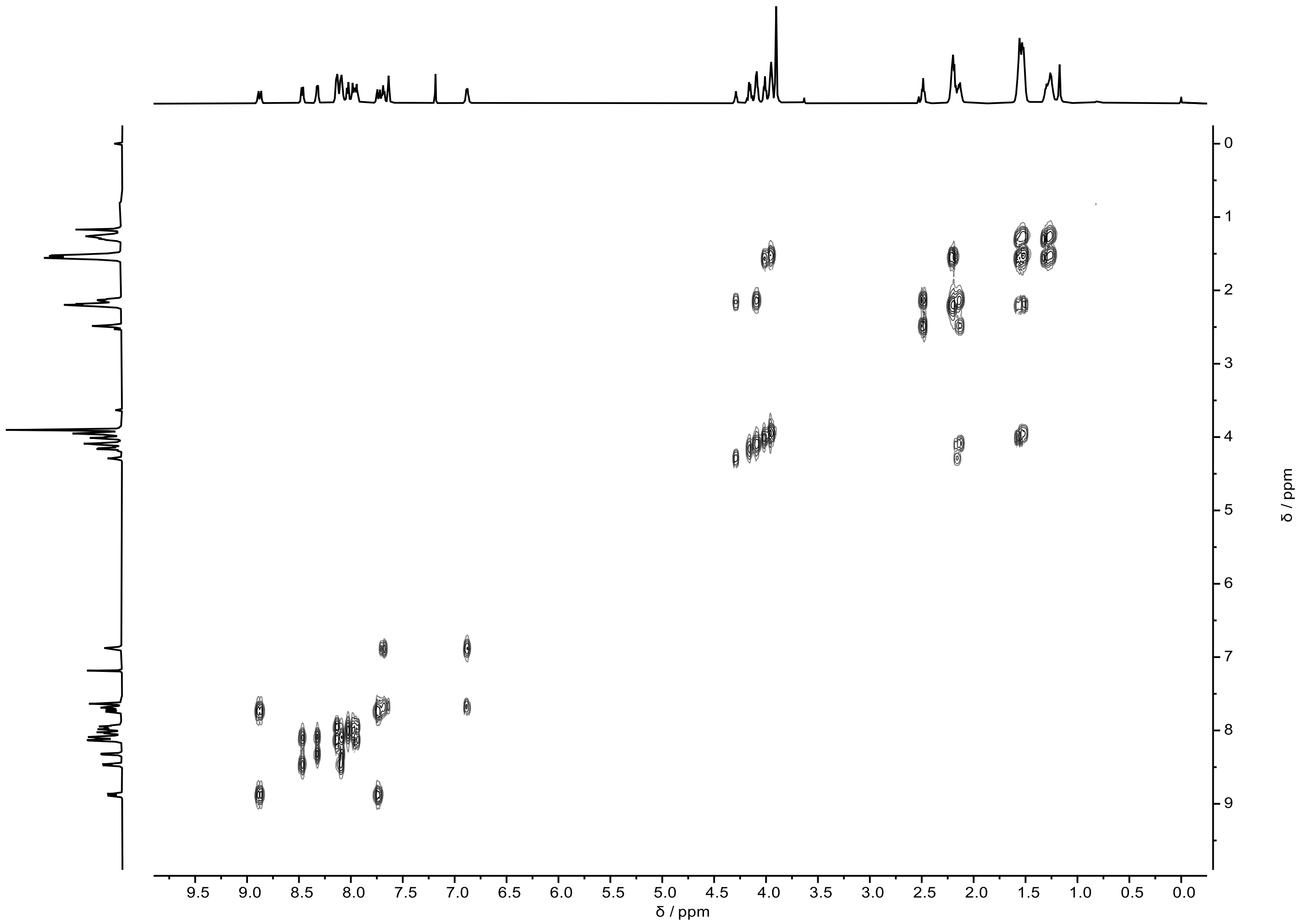

Supplementary Fig. 33. COSY NMR spectrum of **T3** in $CDCl_3$.

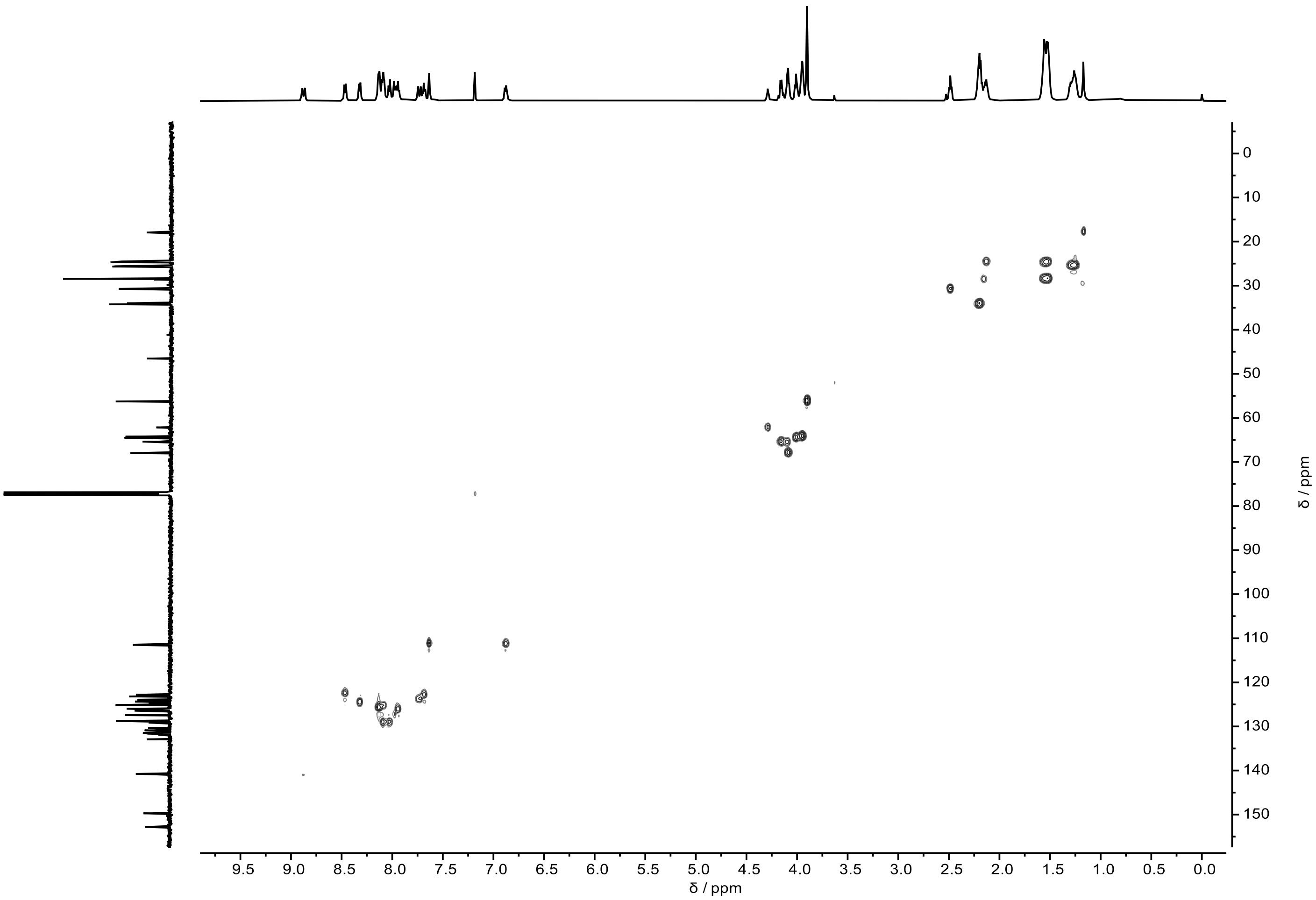

Supplementary Fig. 34. HSQC NMR spectrum of **T3** in $CDCl_3$.

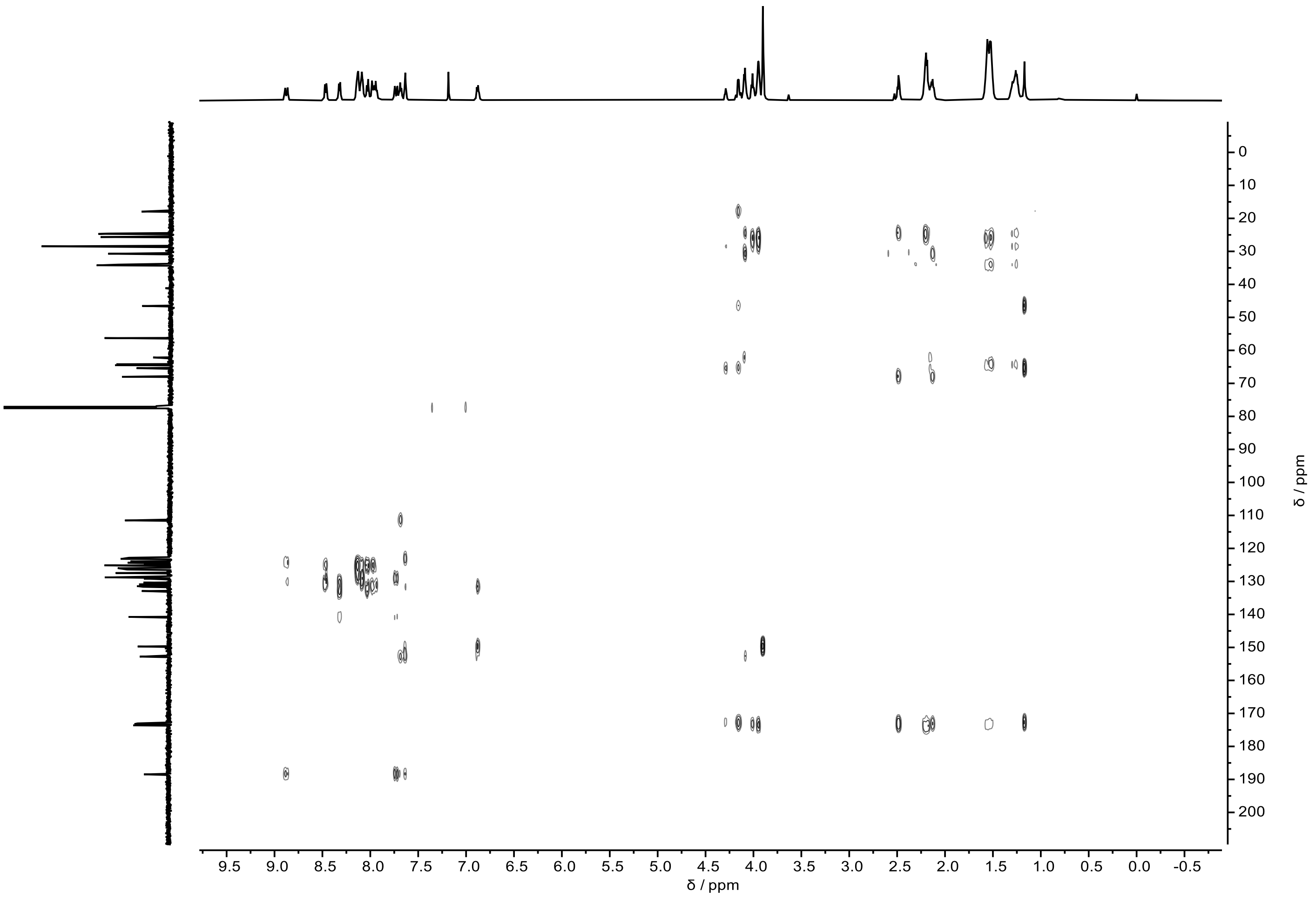

Supplementary Fig. 35. HMBC NMR spectrum of **T3** in $CDCl_3$.

2.6.2.4 T5

^{1}H NMR (400 MHz, $CDCl_3$) δ 8.97 (dd, *J* = 15.3, 3.1 Hz, 3H), 8.61 – 8.52 (m, 3H), 8.43 (dd, *J* = 8.1, 4.3 Hz, 3H), 8.28 – 7.97 (m, 21H), 7.87 – 7.67 (m, 9H), 6.97 (dd, *J* = 8.5, 4.9 Hz, 3H), 4.37 (t, *J* = 6.2 Hz, 2H), 4.27 – 4.21 (m, 4H), 4.18 (td, *J* = 6.6, 3.3 Hz, 6H), 4.12 – 4.00 (m, 20H), 3.98 (d, *J* = 2.0 Hz, 9H), 2.61 – 2.51 (m, 4H), 2.36 – 2.15 (m, 26H), 1.71 – 1.54 (m, 40H), 1.45 – 1.28 (m, 20H), 1.25 (d, *J* = 1.7 Hz, 3H).

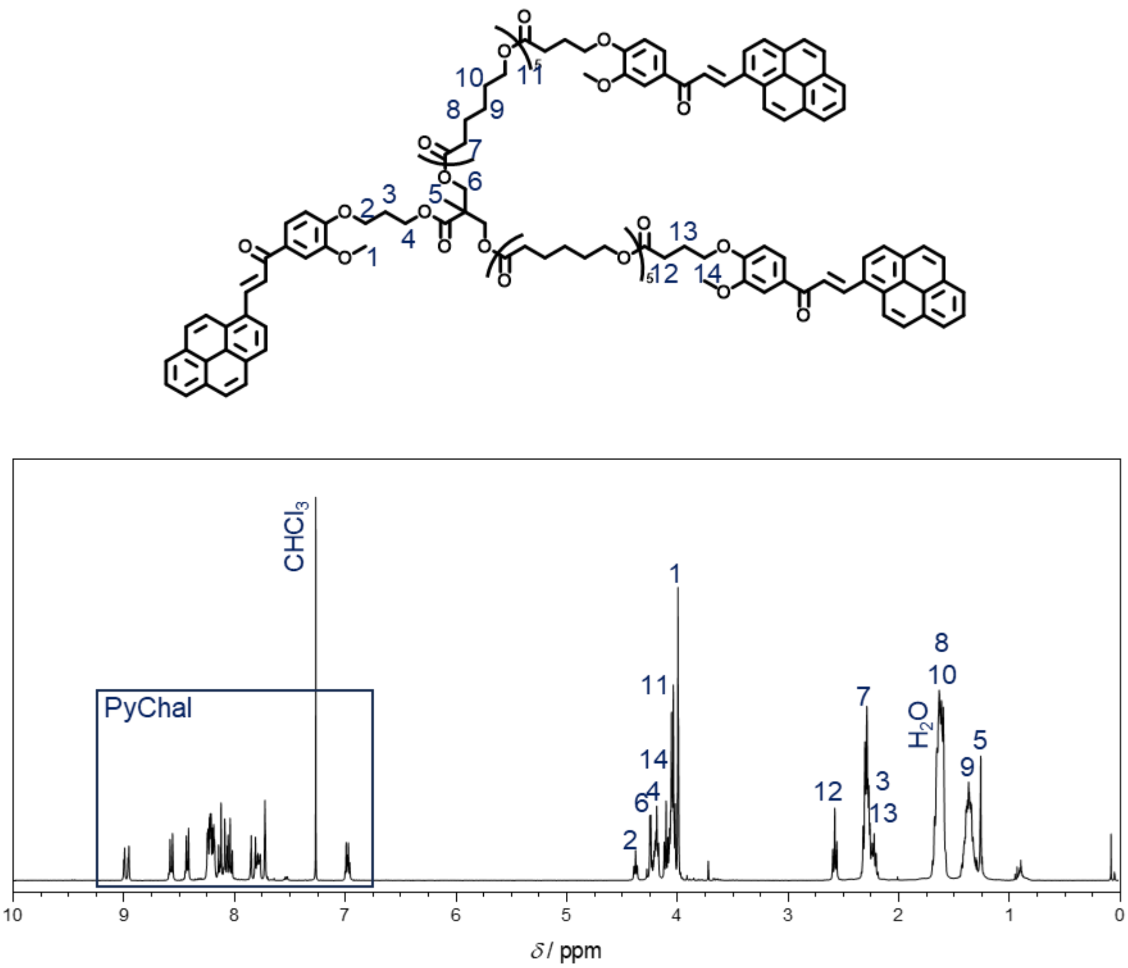

Supplementary Fig. 36. ^{1}H NMR spectrum of **T5** in $CDCl_3$.

^{13}C NMR (101 MHz, $CDCl_3$) δ 188.51, 188.39, 173.67, 173.64, 173.22, 173.02, 172.79, 152.82, 152.62, 149.74, 140.91, 140.80, 132.99, 132.95, 131.93, 131.68, 131.47, 130.90, 130.40, 129.20, 129.12, 128.83, 128.80, 128.78, 127.48, 126.45, 126.19, 126.15, 126.04, 126.01, 125.18, 125.14, 124.78, 124.37, 123.99, 123.81, 123.21, 123.12, 122.85, 122.81, 111.54, 111.49, 111.46, 68.01, 65.56, 65.40, 64.53, 64.29, 64.27, 64.19, 62.16, 56.28, 56.24, 46.55, 34.25, 34.21, 34.00, 30.73, 28.63, 28.48, 28.46, 25.68, 25.66, 25.60, 24.71, 24.69, 24.59, 24.50, 17.94.

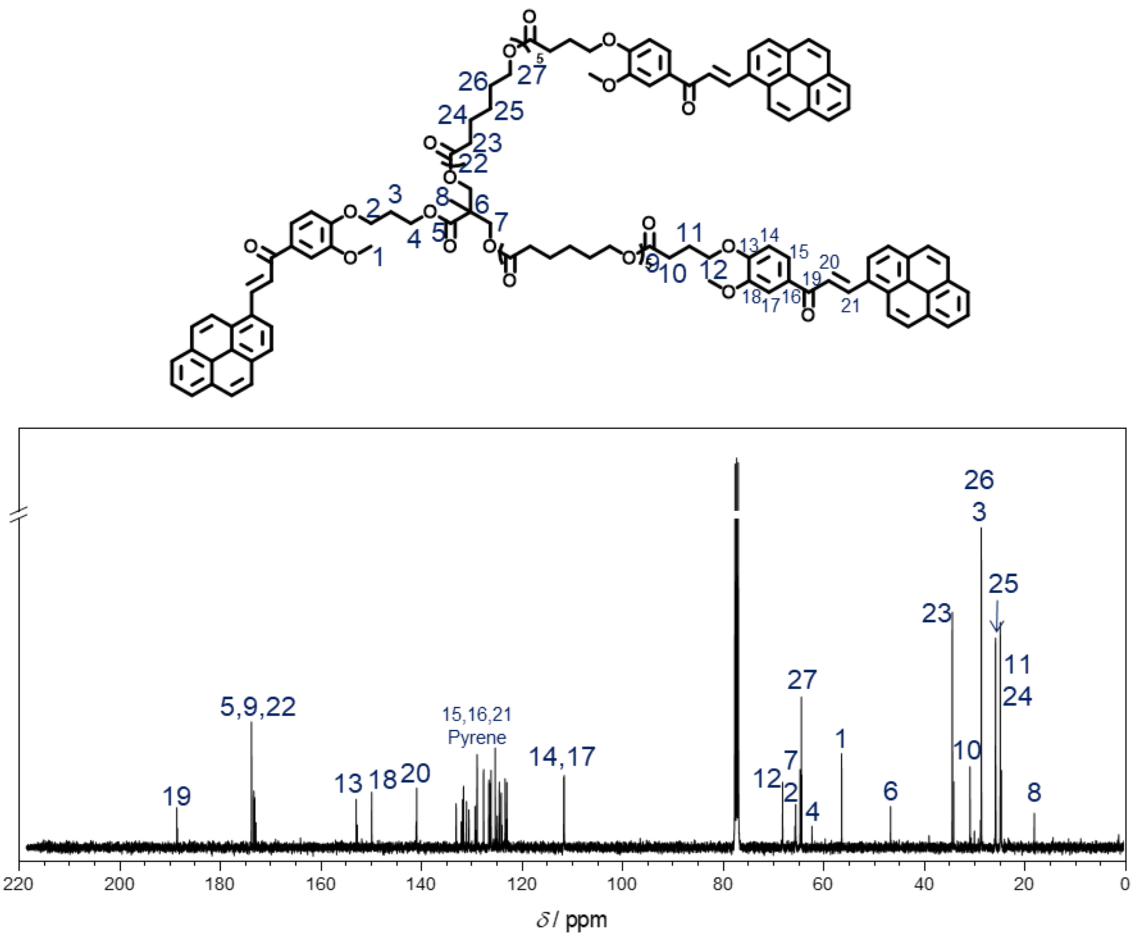

Supplementary Fig. 37. ^{13}C NMR spectrum of **T5** in $CDCl_3$.

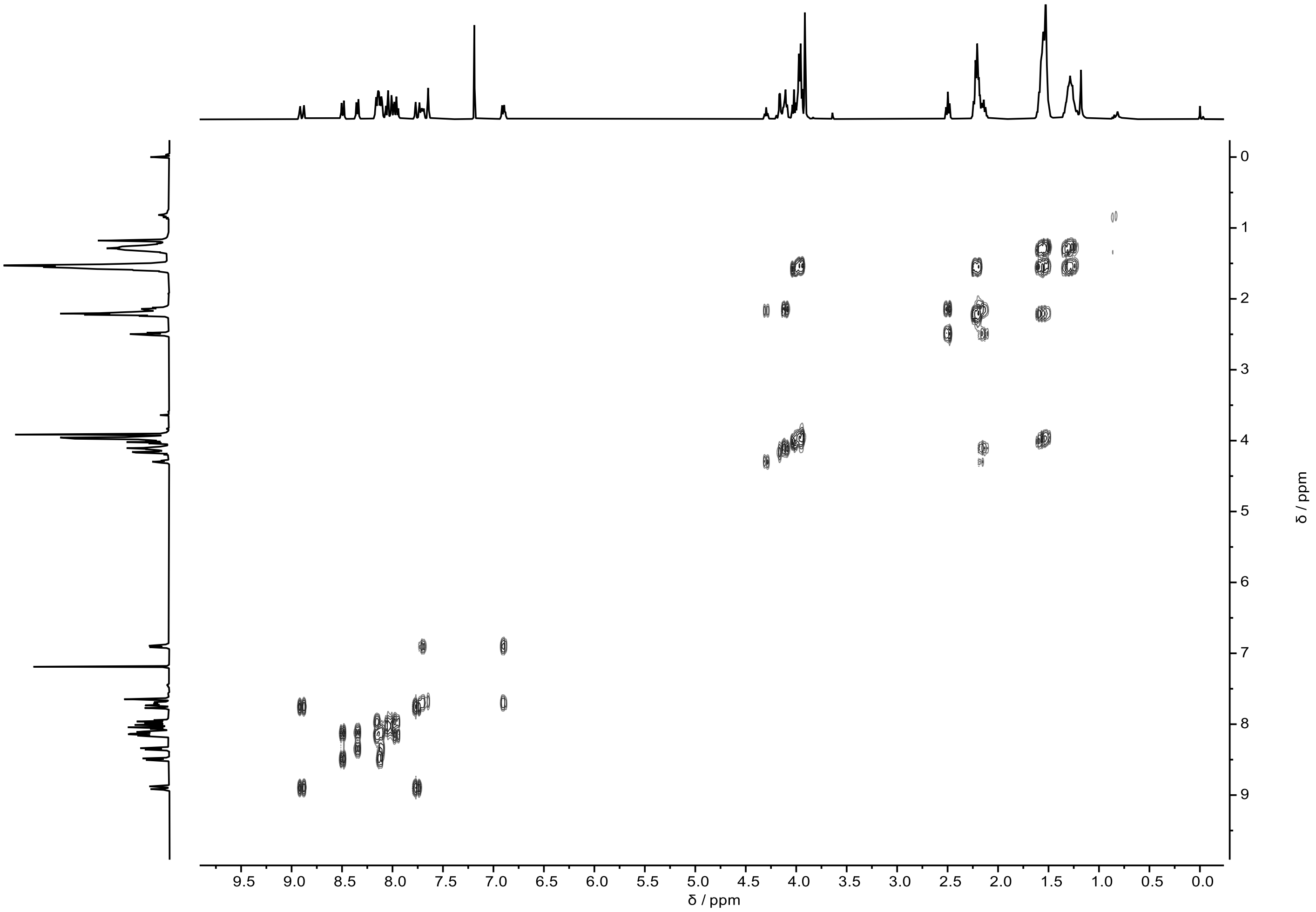

Supplementary Fig. 38. COSY NMR spectrum of **T5** in $CDCl_3$.

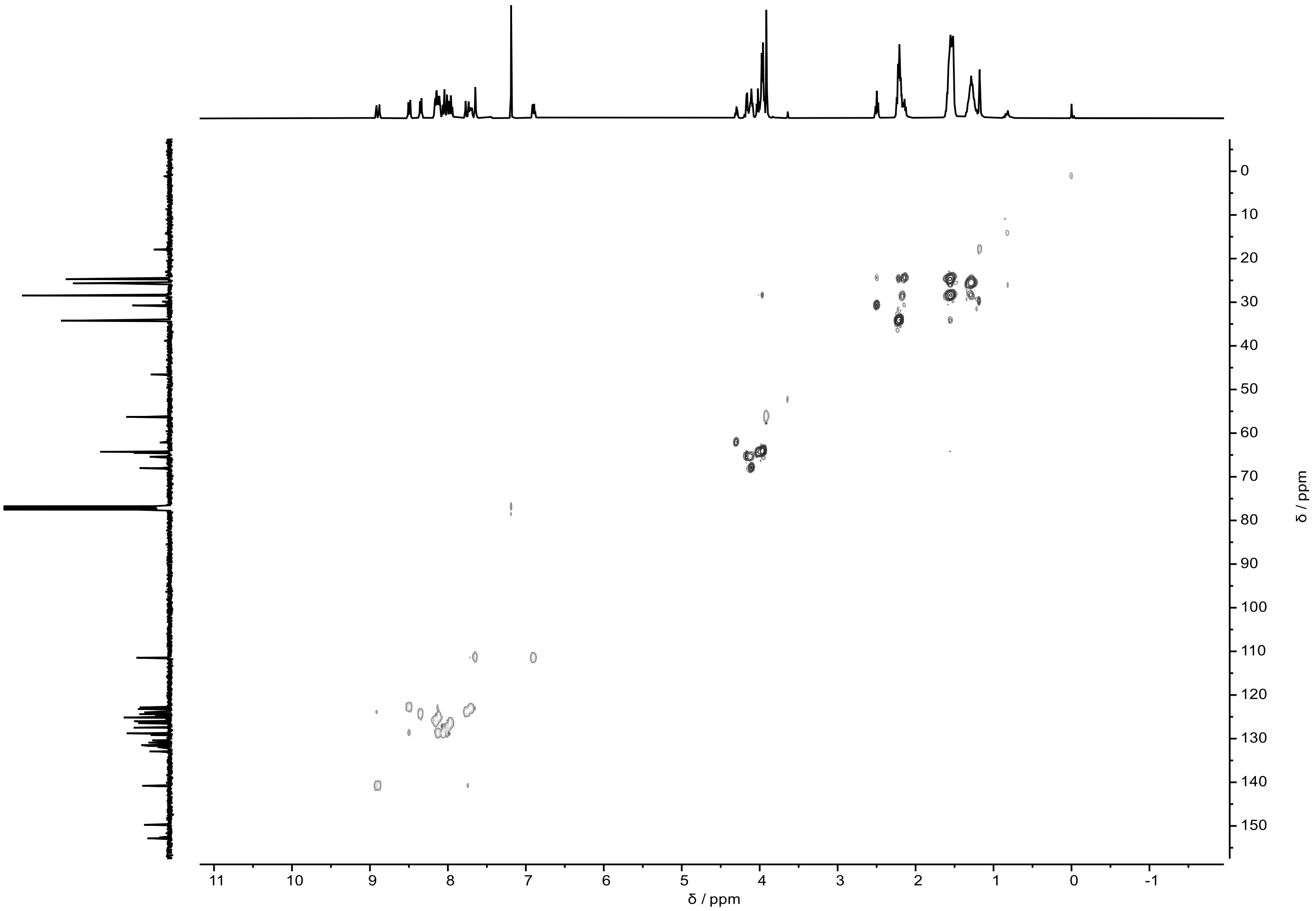

Supplementary Fig. 39. HSQC NMR spectrum of **T5** in $CDCl_3$.

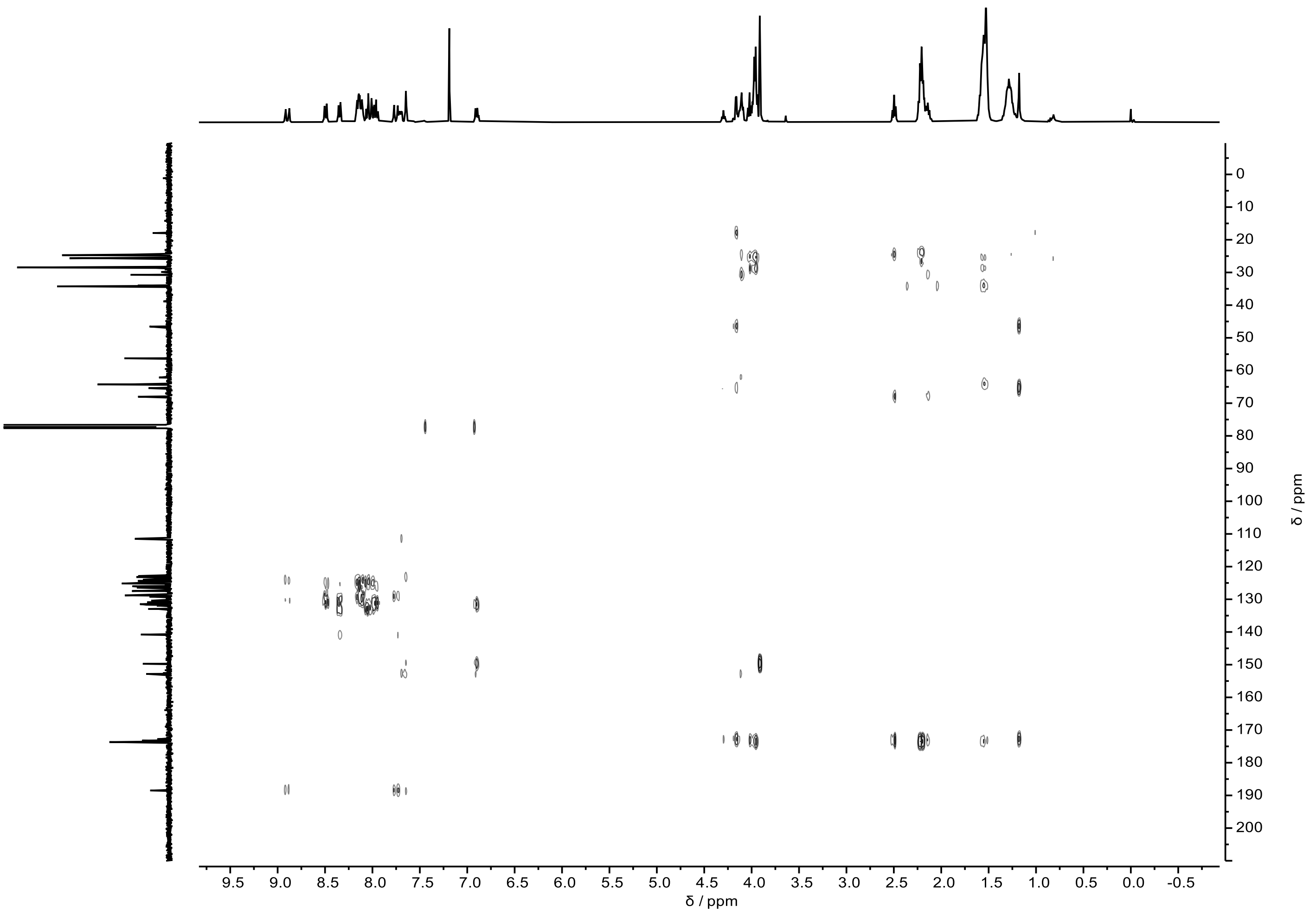

Supplementary Fig. 40. HMBC NMR spectrum of **T5** in $CDCl_3$.

2.6.3 HRMS Characterisation of each macromolecule

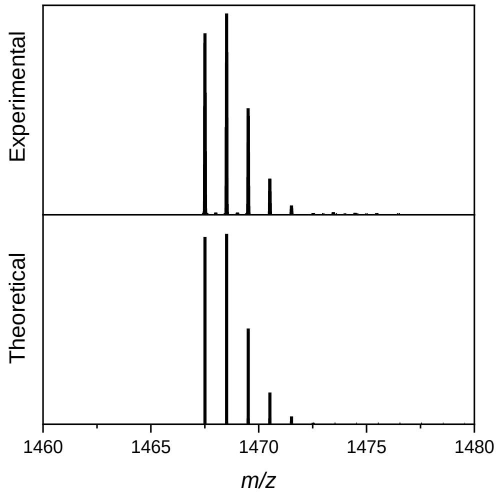

Supplementary Fig. 41. HRMS spectrum of **T0** showing the experimental (top) and calculated (bottom) spectra.

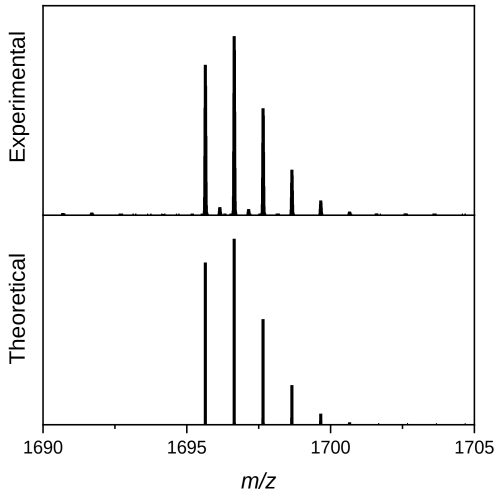

Supplementary Fig. 42. HRMS spectrum of **T1** showing the experimental (top) and calculated (bottom) spectra.

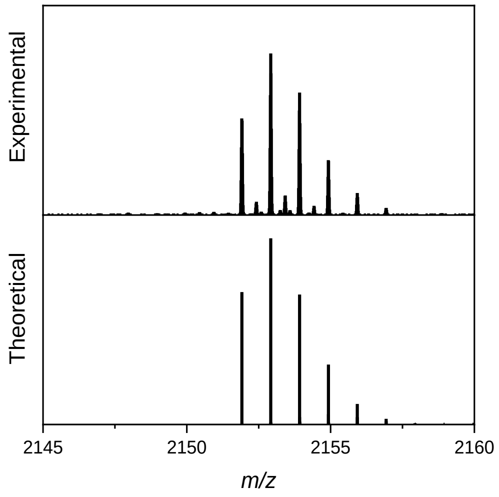

Supplementary Fig. 43. HRMS spectrum of **T3** showing the experimental (top) and calculated (bottom) spectra.

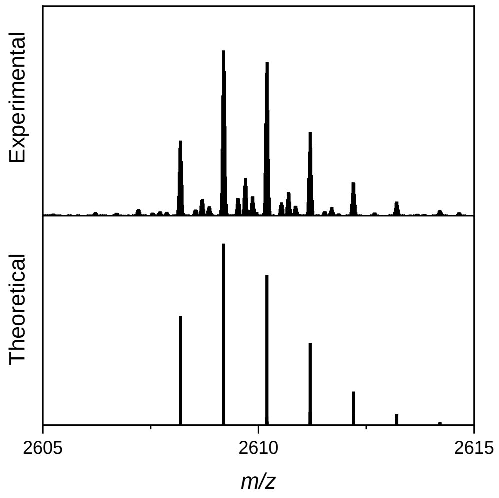

Supplementary Fig. 44. HRMS spectrum of **T5** showing the experimental (top) and calculated (bottom) spectra.

2.6.4 Tabulated HRMS data for each molecule

Supplementary Table 1. High resolution mass spectrometry data for the characterisation of each macromolecule **T0-5**.

Macromolecule	*Ion*	*Theoretical m/z*	*Experimental m/z*	*Δ / ppm*
**T0**	$[C_{94}H_{76}O_{15}+Na]^{+}$	1467.5076	1467.5076	±0.00
**T1**	$[C_{106}H_{96}O_{19}+Na]^{+}$	1695.6438	2695.6455	+1.00
**T3**	$[C_{130}H_{136}O_{27}+Na]^{+}$	2152.9195	2152.9186	-0.42
**T5**	$[C_{154}H_{176}O_{35}+Na]^{+}$	2609.1918	2609.1943	+0.96

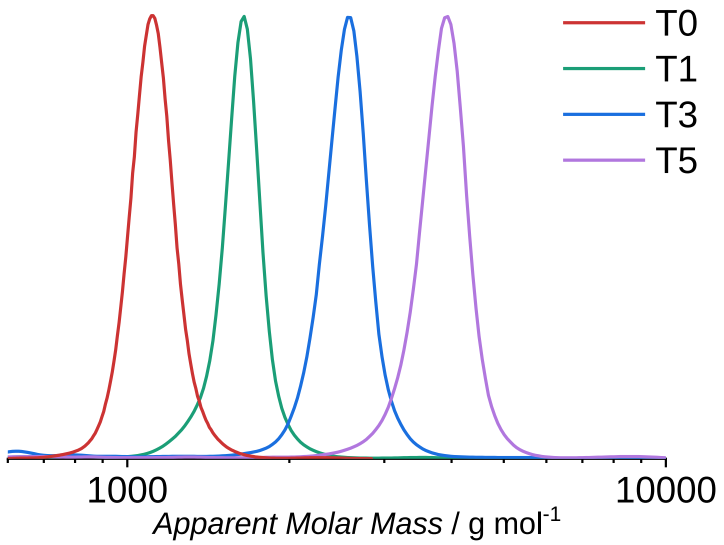

Supplementary Fig. 45. Size Exclusion Chromatographs of **T0-5** recorded in THF.

2.7 Quantum yield determination

2.7.1 Experimental setup for quantum yield determination

Quantum yield was determined using a tuneable laser setup that has been described previously by our group.[6] An Opotek Opolette 335 OPO producing 7 ns, 20 Hz pulses with a flattop spatial profile was used as the pulse light source with a monodisperse wavelength of 445 nm. The beam was initially passed through a beam expander to ensure uniform illumination of the sample, and was then columnated before being redirected into the bottom of the sample by a prism. The sample is held in a 3D-printed cuvette holder that has fibre-optic inputs on each side and a window for irradiation from below. On one side of the sample, a broad band light source with low intensity irradiates the sample, and on the other an online-photospectrometer records the real-time absorbance spectrum.

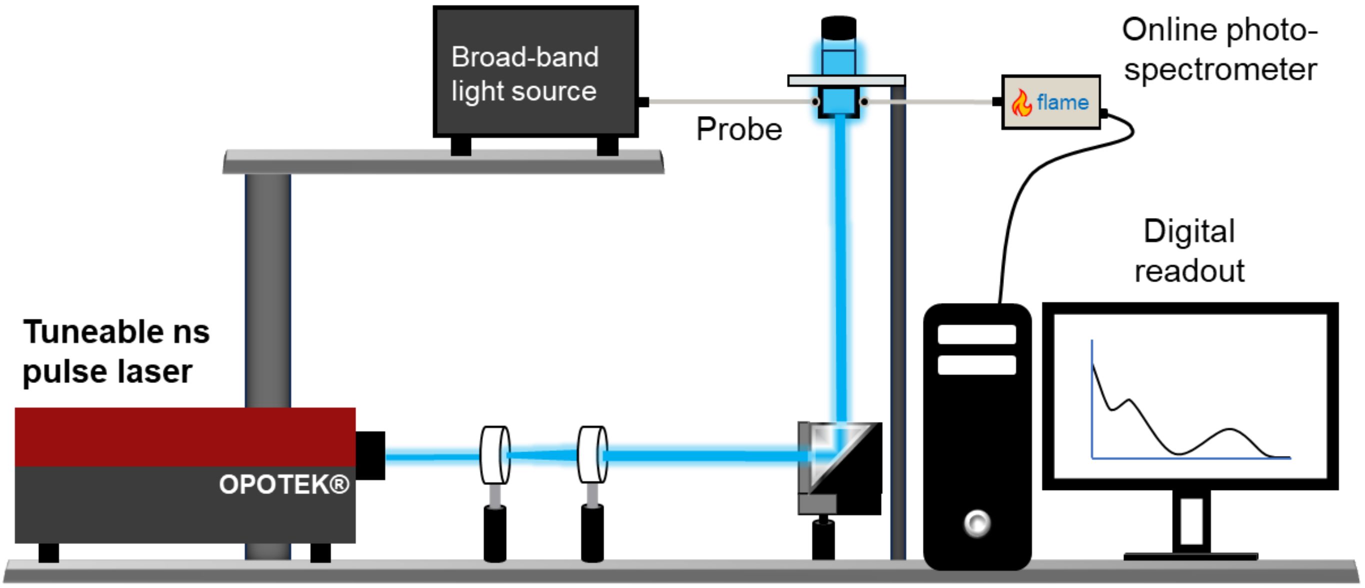

Supplementary Fig. 46. Schematic representation of the laser setup used in this study

All quantum yield experiments were conducted at a concentration of 25 µM in acetonitrile, with each sample containing 2.5 mL. All concentrations were normalised by UV/vis spectroscopy.

2.7.2 Derivation of formula for quantum yield determination

The quantum yield was determined from the experimental data by fitting the linear section of the conversion *vs* number of photons graph for each of the four molecules with **equation (2.5)**. This equation was derived from **equation (2.1)** that states that the quantum yield (Φ_c) is equal to the ratio of half the number of molecules converted (N_c), to the number of photons absorbed ($N_{p,abs}$). N_c can be expressed as the product of the conversion (ρ) and the number of molecules present (N_m) to give **equation (2.2)** that can be further transformed into **equation (2.3)** by using the relationship between number of moles (n) and Avogadro's Number (N_A). n can subsequently be expressed as the product of concentration (c) and volume (V) to give **equation (2.4)** that can subsequently be rearranged to give **equation (2.5)**. Then, $N_{p,Abs}$ is expressed in terms of the product of the incident photons (N_p) and the fraction of photons absorbed, where A is the extinction of the molecule at $\lambda = \lambda_{excitation}$ this gives **equation (2.6)**.

$$\Phi_c = \frac{N_c}{2N_{p,Abs}} \tag{2.1}$$

$$\Phi_c = \frac{\rho \cdot N_m}{2N_{p,Abs}} \tag{2.2}$$

$$\Phi_c = \frac{\rho \cdot n \cdot N_A}{2N_{p,Abs}} \tag{2.3}$$

$$\Phi_c = \frac{\rho \cdot c \cdot V \cdot N_A}{2N_{p,Abs}} \tag{2.4}$$

$$\rho = \frac{2\Phi_c \cdot N_{p,Abs}}{c \cdot V \cdot N_A} \tag{2.5}$$

$$\rho = \frac{2\Phi_c \cdot N_p(1 - 10^{-A})}{c \cdot V \cdot N_A} \tag{2.6}$$

When **equation (2.6)** is used to fit the conversion *vs* number of photon curves, the gradient can be expressed as $\Phi_c \cdot \frac{2(1-10^{-A})}{c \cdot V \cdot N_A}$, thus the quantum yield can be determined by fitting the linear section of the conversion *vs* photons profile.

2.7.3 Extinction spectra of T0-5

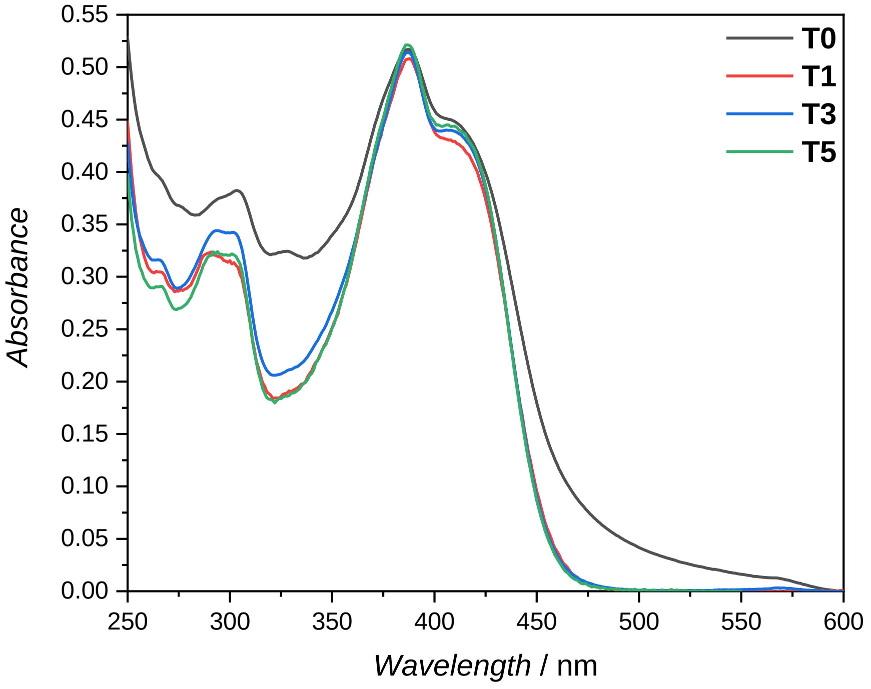

Supplementary Fig. 47. Extinction spectra of **T0-5** at a concentration of 25 µM in acetonitrile.

Supplementary Table 2. Values of *A* used to calculate the quantum yield for each molecule.

Molecule	Extinction at λ = 445 nm
**T0**	0.2228
**T1**	0.1435
**T3**	0.1401
**T5**	0.1355

2.7.4 Quantum yield fits

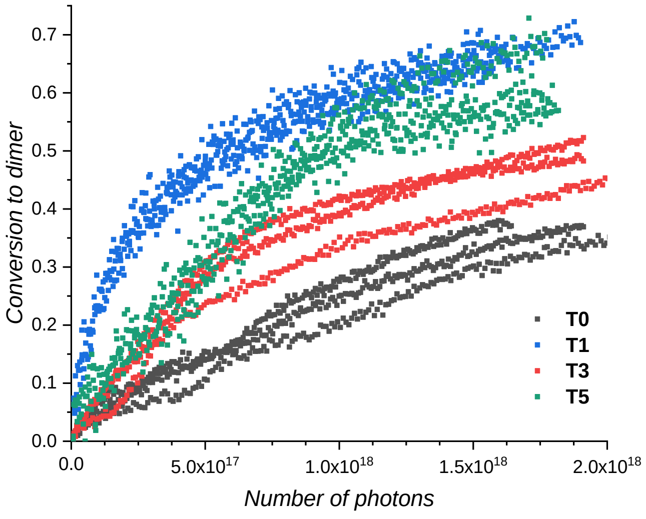

Supplementary Fig. 48. Conversion versus number of photons for molecules **T0-5**.

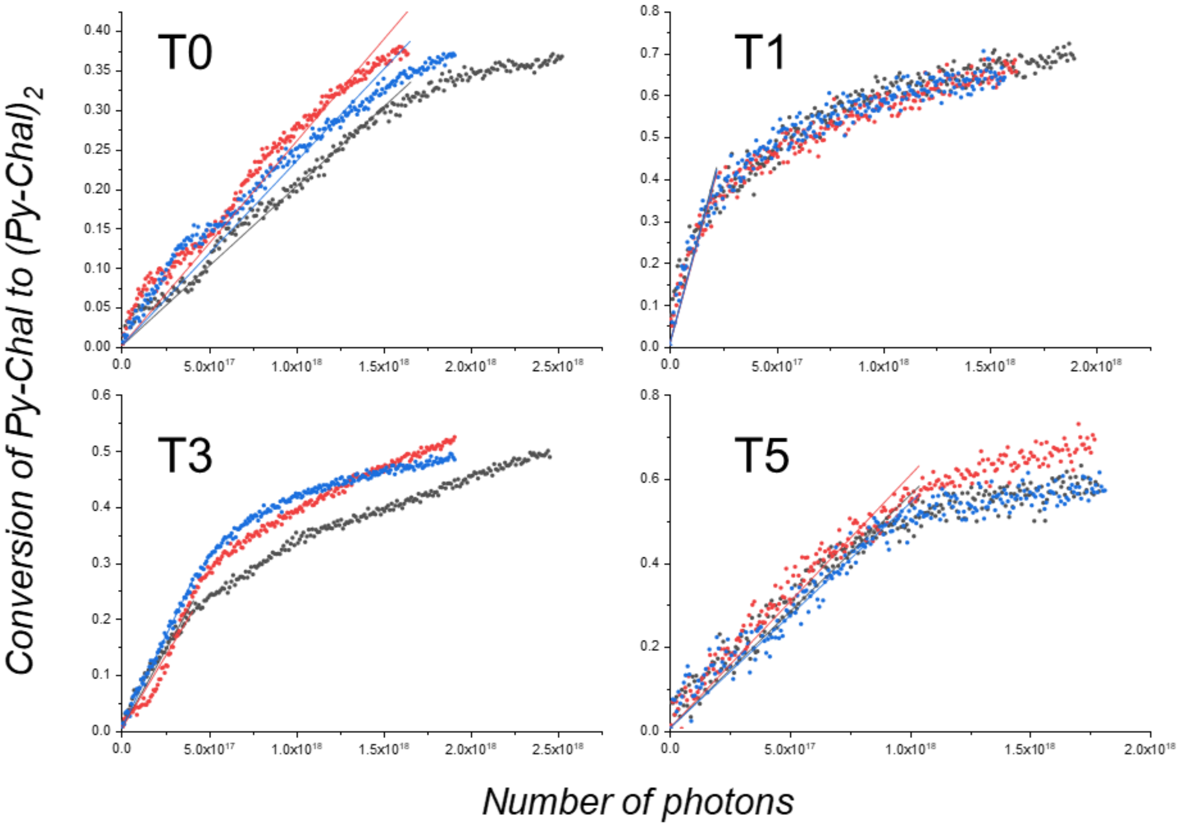

Supplementary Fig. 49. conversion *versus* number of photons plots for each of the three molecules as monitored

Supplementary Table 3. Tabulated quantum yield data for each macromolecule.

Molecule	Slope	Quantum Yield	Mean Quantum Yield	Standard deviation
**T0**	2.019E-19	9.465E-03	1.085E-02	0.00109
	2.588E-19	1.213E-02		
	2.335E-19	1.095E-02		
**T1**	1.915E-18	1.280E-01	1.258E-01	0.001754
	1.850E-18	1.237E-01		
	1.881E-18	1.258E-01		
**T3**	5.565E-19	3.797E-02	3.920E-02	0.003919
	5.149E-19	3.513E-02		
	6.521E-19	4.449E-02		
**T5**	5.557E-19	3.900E-02	3.966E-02	0.001939
	6.025E-19	4.229E-02		
	5.368E-19	3.768E-02		

2.8 Molecular dynamics simulations

Simulation parameters for the central pyrene-chalcone (**PyChal,** ATB molid: 1423694**)**, terminal **PyChal** (ATB molid: 1423693) and methyl 6-hydroxyhexanoate linker (ATB molid: 1423695) subunits of **T0, T1, T3** and **T5** were developed individually using the Automated topology Builder[7] and compatible with the GROMOS 54A7 molecular dynamics force field[8] for each subunit. Each subunit was capped at its functionalization points to incorporate an overlap region of neighboring units, at least 3 bonds deep. Atomic coordinates and molecular topologies for the complete macromolecule were developed in Chimera 1.17.1[9] by computationally ligating the appropriate number of monomer units at their overlapping functionalization points to reproduce each experimental trifunctional molecule. In **T1**, **T3** and **T5**, the two terminal **PyChal** are connected to the central **PyChal** through linker fragments. In contrast, the central **PyChal** of **T0** is directly connected to the two terminal **PyChal** units.

All simulations were prepared and performed using GROMACS 2023[10] in conjunction with the GROMOS 54A7 forcefield.[8] Four different simulations systems were prepared, corresponding to the macromolecules **T0, T1, T3** and **T5**. In each simulation, a single macromolecule was placed in a cubic box and solvated in acetonitrile (ATB molid: 913062). The box was of sufficient size such that the polymer was at least 1.0 nm from any box edge at the start of the simulation. Each simulation system was energy minimized using a steepest descent algorithm[11] and equilibrated under NPT condition using 1 fs and 2 fs timestep for 1 ns in each case. During the equilibration process, the pressure was maintained at 1 bar using a Berendsen barostat[12] with an isothermal compressibility of 4.5×10^{-5} bar; and the temperature was maintained at 300 K using the Bussi-Donadio-Parrinello velocity-rescaling thermostat[13] with a coupling constant of 0.1 ps. Non-covalent interactions were calculated with a 1.0 nm cutoff in all simulations. Following equilibration, 500 ns production simulations were carried out in triplicate using a 2 fs timestep.

Analysis was performed on trajectory frames spaced at 200 ps intervals. The pairwise distance between photoactive groups in each arm were calculated using the first carbon atom of the double bond in each **PyChal** arm (i.e., the double bond carbon atom closest to the carbonyl moiety, shown in **SI Figure 8.2**) and the corresponding carbon atom in the opposing **PyChal** arms.

To determine the relative populations of specific conformations, the replicate trajectories for the **T0, T1, T3** and **T5** simulations, respectively, were clustered using the Gromos clustering algorithm. The Gromos algorithm uses an RMSD cut-off value to count the number of neighbors for the conformation sampled in each frame of the trajectory. The structure with the largest number of neighbors is then chosen as the central conformation of the first cluster, and all of its neighbors are included in this cluster. This first cluster is then removed from the pool of conformations and clustering continues until all conformations have been assigned to a conformational cluster. The most populated cluster contains the conformation with the largest number of neighbors, and is the predominant cluster sampled, representing at least 86 % of the trajectory. Due to the size differences between **T0, T1, T3** and **T5**, the RMSD cut-off values were adjusted to maintain the cluster population size of the predominant conformation for each macromolecule. This corresponded to RMSD cutoff values of 0.70, 0.85, 0.98 and 1.15 nm for the **T0, T1, T3** and **T5** systems, respectively.

Plots of the simulation data were generated using Matplotlib 3.5.2.[14] Trajectory visualization, analysis and image rendering were done using VMD version 1.9.4.[15]

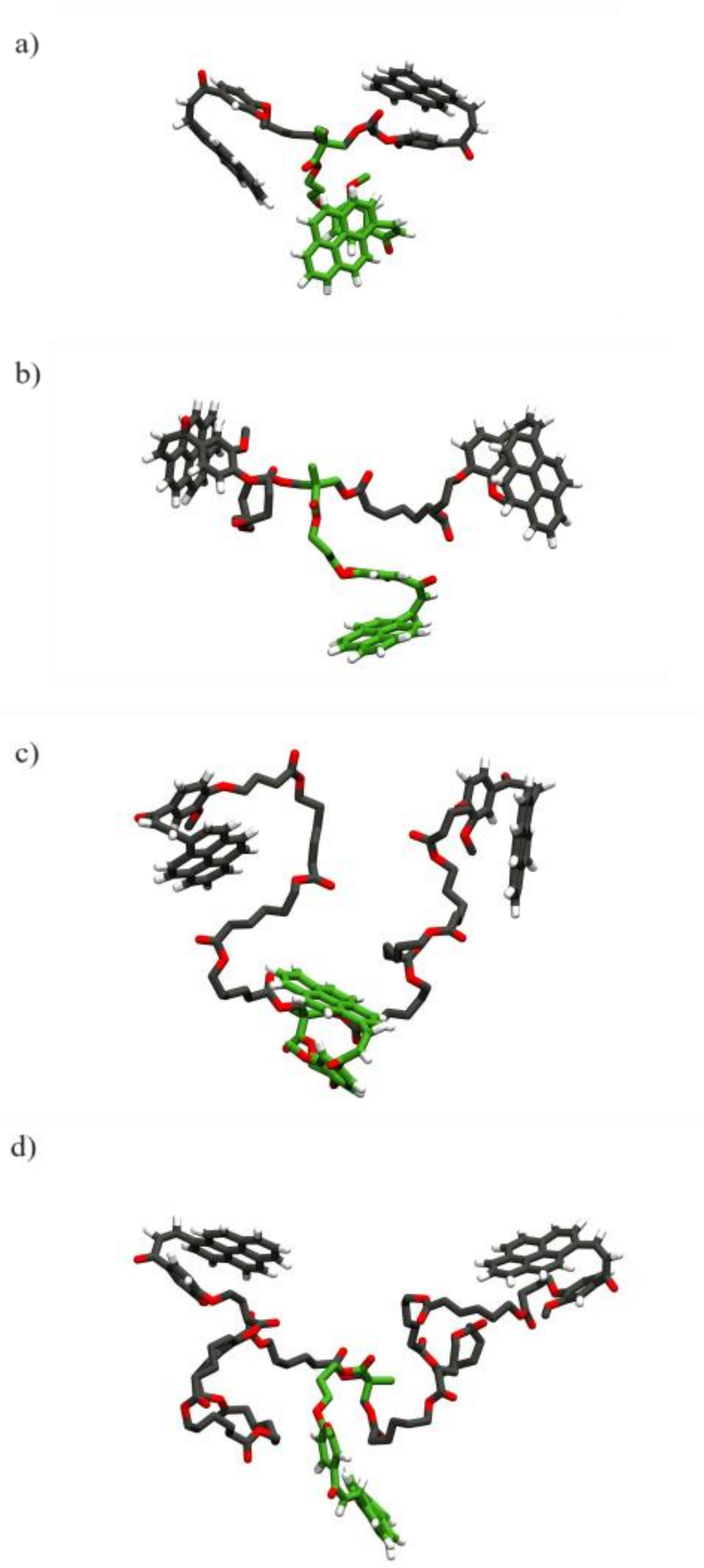

Supplementary Fig. 50. Stable conformations extracted from trajectories with GROMACS version 2023 cluster analysis for **a) T0**, **b) T1**, **c) T3**, and **d) T5** showing the π stacking interaction between chalcone ring system and benzene ring in same arms. Carbons in the terminal **PyChal** arms are shown in black, carbons in the middle **PyChal** arm are shown in green and oxygens are shown in red.

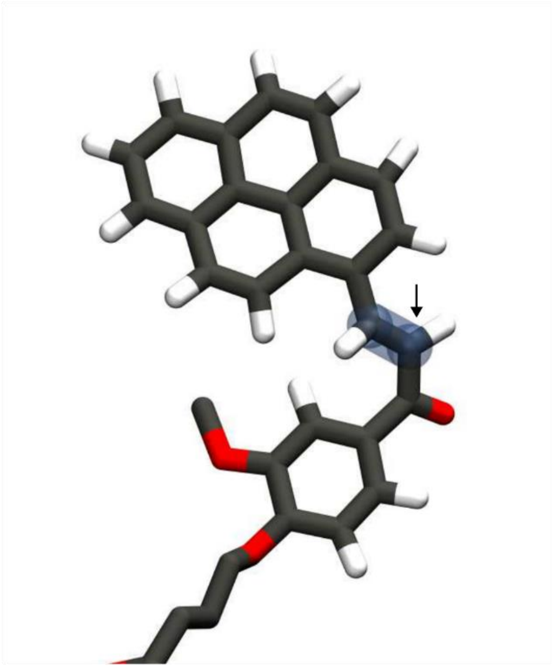

Supplementary Fig. 51 Fragment of one **PyChal** arm showing the carbon atom (shown with an arrow) used for the pair distance calculation between photoactive double bonds (transparent blue) of each **PyChal** arm.

3. Supplementary Discussion

3.1 Confirmation of cycloaddition

1 mg of **T0** was dissolved in acetonitrile at a concentration of 12.5 μM and irradiated with a 10 W LED (λ_{max} = 445 nm) for 3.5 minutes. The acetonitrile was then removed, and the residue submitted to NMR analysis that is presented in **Supplementary Fig. 52**.

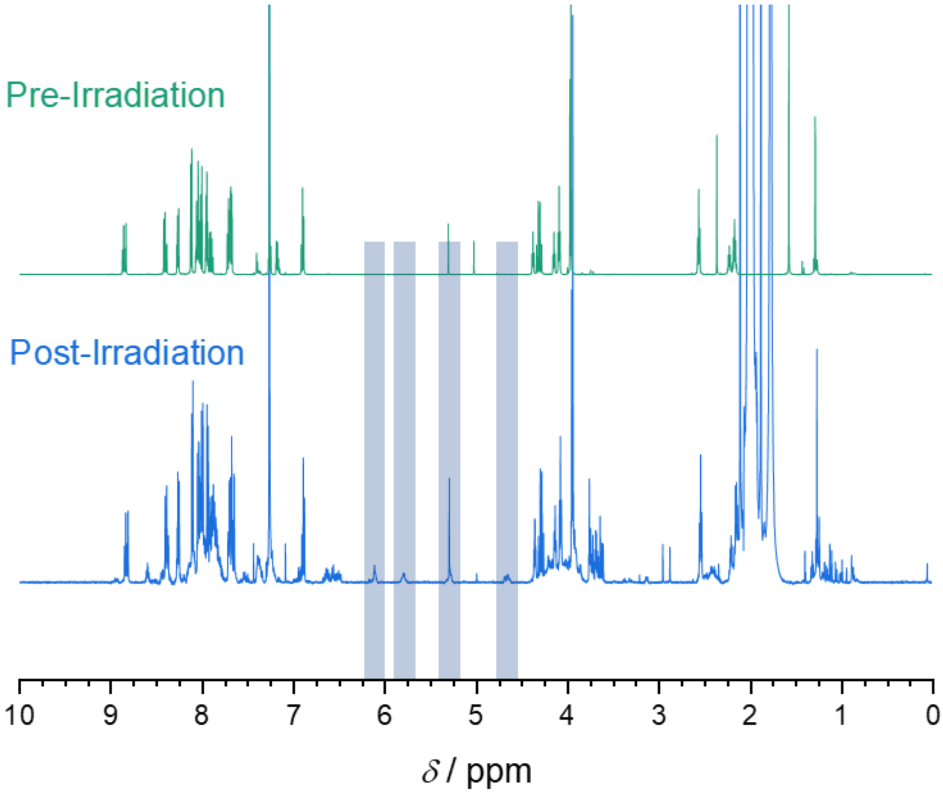

Supplementary Fig. 52. ^{1}H NMR spectra of **T0** before (green) and after (blue) irradiation, with blue highlights showing the formation of cyclobutane resonances.

4. Supplementary Figures

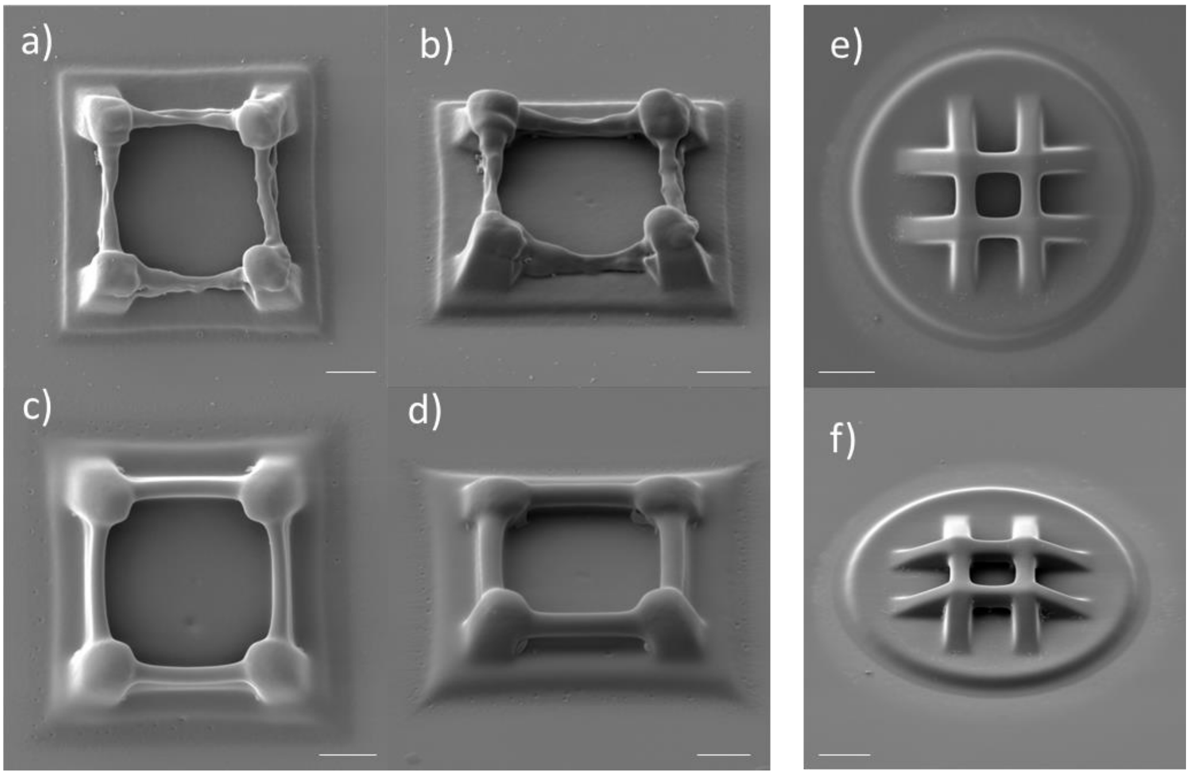

Supplementary Fig. 53. a. SEM image of a boxing ring printed with **T3** using laserpower = 15, scan speed = 1,500 µm s^{-1}, scale bar = 5 µm; **b**. angled SEM image of the same structure, scale bar = 5 µm; **c**. SEM image of a boxing ring printed with **T5** using laser power = 70, scan speed = 12,500 µm s^{-1}, scale bar = 5 µm; **d**. angled SEM image of the same structure, scale bar = 5 µm; **e.** SEM image of a larger boxing ring structure printed with a higher concentration (138 µmol L^{-1}) solution of **T5** at laserpower = 90, scan speed = 25,000 µm s^{-1}, scale bar = 20 µm; **f**. SEM image of the same structure from a 45° angle, scale bar = 20 µm

5. Supplementary References

1 Gruendling, T., Guilhaus, M. & Barner-Kowollik, C. Quantitative LC-MS of polymers: determining accurate molecular weight distributions by combined size exclusion chromatography and electrospray mass spectrometry with maximum entropy data processing. *Anal. Chem.* **80**, 6915-6927, doi:10.1021/ac800591j (2008).

2 Takizawa, K., Tang, C. & Hawker, C. J. Molecularly defined caprolactone oligomers and polymers: synthesis and characterization. *J. Am. Chem. Soc.* **130**, 1718-1726, doi:10.1021/ja077149w (2008).

3 Duan, S. H. *et al.* A Versatile Synthetic Platform for Discrete Oligo- and Polyesters Based on Optimized Protective Groups Iterative Exponential Growth. *Macromolecules* **54**, 10830-10837, doi:10.1021/acs.macromol.1c01498 (2021).

4 Irshadeen, I. M. *et al.* Green light LED activated ligation of a scalable, versatile chalcone chromophore. *Polym. Chem.* **12**, 4903-4909, doi:10.1039/d1py00533b (2021).

5 Yeo, J.-S. & Hwang, S.-H. The effect of dense polymer brush on the microfibrillated cellulose for the mechanical properties of poly(ε-caprolactone) biocomposites. *Int. J. Adhes. Adhes.* **78**, 89-94, doi:10.1016/j.ijadhadh.2017.06.023 (2017).

6 Irshadeen, I. M. *et al.* Action Plots in Action: In-Depth Insights into Photochemical Reactivity. *J. Am. Chem. Soc.* **143**, 21113-21126, doi:10.1021/jacs.1c09419 (2021).

7 Malde, A. K. *et al.* An Automated Force Field Topology Builder (ATB) and Repository: Version 1.0. *J Chem Theory Comput* **7**, 4026-4037, doi:10.1021/ct200196m (2011).

8 Schmid, N. *et al.* Definition and testing of the GROMOS force-field versions 54A7 and 54B7. *Eur Biophys J* **40**, 843-856, doi:10.1007/s00249-011-0700-9 (2011).

9 Pettersen, E. F. *et al.* UCSF Chimera--a visualization system for exploratory research and analysis. *J. Comput. Chem.* **25**, 1605-1612, doi:10.1002/jcc.20084 (2004).

10 Abraham, M. J. *et al.* GROMACS: High performance molecular simulations through multi-level parallelism from laptops to supercomputers. *SoftwareX* **1-2**, 19-25, doi:10.1016/j.softx.2015.06.001 (2015).

11 Meza, J. C. Steepest descent. *WIREs Computational Statistics* **2**, 719-722, doi:10.1002/wics.117 (2010).

12 Berendsen, H. J. C., Postma, J. P. M., van Gunsteren, W. F., DiNola, A. & Haak, J. R. Molecular dynamics with coupling to an external bath. *The Journal of Chemical Physics* **81**, 3684-3690, doi:10.1063/1.448118 (1984).

13 Bussi, G., Donadio, D. & Parrinello, M. Canonical sampling through velocity rescaling. *J. Chem. Phys.* **126**, 014101, doi:10.1063/1.2408420 (2007).

14 Hunter, J. D. Matplotlib: A 2D Graphics Environment. *Computing in Science & Engineering* **9**, 90-95, doi:10.1109/MCSE.2007.55 (2007).

15 William, H., Andrew, D. & Klaus, S. VMD: Visual molecular dynamics. *Journal of Molecular Graphics* **14**, 33-38, doi:10.1016/0263-7855(96)00018-5 (1996).
