# Supplementary material for: How molecular architecture defines quantum yields
Source: Nat Commun. 2024 Jul 17;15:6033. doi: 10.1038/s41467-024-50366-1 (PMC11255304; doi:10.1038/s41467-024-50366-1)
Supplement: Supplementary file 2 — Peer Review File [file 41467_2024_50366_MOESM2_ESM.pdf]

nature portfolio

Peer Review File

How Molecular Architecture Defines Quantum Yields

Open Access This file is licensed under a Creative Commons Attribution 4.0 International License, which permits use, sharing, adaptation, distribution and reproduction in any medium or format, as long as you give appropriate credit to the original author(s) and the source, provide a link to the Creative Commons license, and indicate if changes were made. In the cases where the authors are anonymous, such as is the case for the reports of anonymous peer reviewers, author attribution should be to 'Anonymous Referee' followed by a clear attribution to the source work. The images or other third party material in this file are included in the article's Creative Commons license, unless indicated otherwise in a credit line to the material. If material is not included in the article's Creative Commons license and your intended use is not permitted by statutory regulation or exceeds the permitted use, you will need to obtain permission directly from the copyright holder. To view a copy of this license, visit http://creativecommons.org/licenses/by/4.0/.

Editorial Note: This manuscript has been previously reviewed at another journal that is not operating a transparent peer review scheme. This document only contains reviewer comments and rebuttal letters for versions considered at *Nature Communications*.

REVIEWERS' COMMENTS

Reviewer #2 (Remarks to the Author):

[Note from the Editor: This reviewer was asked to additionally look over the responses to the comments from reviewer 1]

I was impressed with the revisions that the authors have made to the paper relevant to my previous criticisms, and those of others. I have also reviewed the authors' responses to reviewers 1 and 3, and I also believe they have responded appropriately to justify publication. The work is high quality, and is now submitted to a journal of appropriate scope and impact. I recommend publication of the work.

Reviewer #3 (Remarks to the Author):

The changes to the manuscript and the replies to reviewers all seem thoughtful and appropriate. This revised manuscript is suitable for publication in Nature Communications. This reviewer thanks the authors for their careful responses and edits.
